# RSM1, an *Arabidopsis* MYB protein, interacts with HY5/HYH to modulate seed germination and seedling development in response to abscisic acid and salinity

**DOI:** 10.1371/journal.pgen.1007839

**Published:** 2018-12-19

**Authors:** Bencan Yang, Zihao Song, Chaonan Li, Jiahao Jiang, Yangyang Zhou, Ruipu Wang, Qi Wang, Chang Ni, Qing Liang, Haodong Chen, Liu-Min Fan

**Affiliations:** State Key Laboratory of Protein and Plant Gene Research, School of Life Sciences, School of Advanced Agricultural Sciences, Peking University, Beijing, China; Wake Forest University, UNITED STATES

## Abstract

MYB transcription factors are involved in many biological processes, including metabolism, development and responses to biotic and abiotic stresses. RADIALIS-LIKE SANT/MYB 1 (RSM1) belongs to a MYB-related subfamily, and previous transcriptome analysis suggests that *RSM1* may play roles in plant development, stress responses and plant hormone signaling. However, the molecular mechanisms of RSM1 action in response to abiotic stresses remain obscure. We show that down-regulation or up-regulation of *RSM1* expression alters the sensitivity of seed germination and cotyledon greening to abscisic acid (ABA), NaCl and mannitol in *Arabidopsis*. The expression of *RSM1* is dynamically regulated by ABA and NaCl. Transcription factors ELONGATED HYPOCOTYL 5 (HY5) and HY5 HOMOLOG (HYH) regulate *RSM1* expression via binding to the *RSM1* promoter. Genetic analyses reveal that *RSM1* mediates multiple functions of HY5 in responses of seed germination, post-germination development to ABA and abiotic stresses, and seedling tolerance to salinity. Pull-down and BiFC assays show that RSM1 interacts with HY5/HYH *in vitro* and *in vivo*. RSM1 and HY5/HYH may function as a regulatory module in responses to ABA and abiotic stresses. RSM1 binds to the promoter of *ABA INSENSITIVE 5* (*ABI5*), thereby regulating its expression, while RSM1 interaction also stimulates HY5 binding to the *ABI5* promoter. However, no evidence was found in the dual-luciferase transient expression assay to support that RSM enhances the activation of *ABI5* expression by HY. In summary, HY5/HYH and RSM1 may converge on the *ABI5* promoter and independently or somehow dependently regulate *ABI5* expression and ABI5-downstream ABA and abiotic stress-responsive genes, thereby improving the adaption of plants to the environment.

## Author summary

The phytohormone abscisic acid (ABA) regulates multiple developmental processes in plants, including seed dormancy and germination, growth, and abiotic stress responses. The transcription factor ELONGATED HYPOCOTYL 5 (HY5), a core regulator of light signaling, is involved in ABA and abiotic stress responses by directly regulating the expression of *ABA INSENSITIVE 5* (*ABI5*). In this study, we show that a MYB-related transcription factor, RADIALIS-LIKE SANT/MYB 1 (RSM1), plays important roles in ABA and salinity signaling in *Arabidopsis*, and we dissect the relationship between RSM1 and HY5. RSM1 interacts with HY5/HYH *in vitro* and *in vivo* and they may function as a regulatory module in responses to ABA and abiotic stresses. RSM1 binds to the promoter of *ABI5*, thereby regulating its expression; moreover, RSM1 interaction stimulates HY5 binding to the *ABI5* promoter, but RSM1 was not found to enhance the activation of *ABI5* expression by HY5. Genetic analyses reveal that *RSM1* mediates the functions of *HY5* in responses of seed germination, post-germination developmental responses to ABA and abiotic stresses, and seedling tolerance to salinity. In summary, our work demonstrates that HY5/HYH and RSM1 may bind with the *ABI5* promoter to regulate *ABI5* expression independently or somehow dependently, thereby controlling the expression of ABI5-downstream target genes in ABA and salinity signaling.

## Introduction

Plants grow in a continuously changing environment that imposes various stresses. Abiotic stresses such as drought and salinity are amongst these environmental stresses [1, 2]. The plant hormone abscisic acid (ABA) is induced by abiotic stresses, and plays essential roles in plant responses and adaptation to those stresses, in addition to regulating several developmental processes, including seed development and maturation, dormancy and germination, seedling growth, and floral transition [3–6].

A family of novel START domain proteins known as PYR/PYLs/RCARs, are the best characterized ABA receptors, although several others have been documented [7, 8]. In the PYR/PYLs/RCARs-initiated core ABA signaling pathway, PYR/PYLs interact with and inhibit clade-A PP2Cs, including ABI1, ABI2, HAB1 and PP2CA/AHG3 [9, 10]. These PP2Cs negatively regulate ABA responses [7] by de-phosphorylating and inhibiting positive regulators of ABA signaling, e.g. a subfamily of ABA-activated SNF1-related protein kinases 2 (SnRK2s) including SnRK2.2, SnRK2.3 and SnRK2.6 in *Arabidopsis* [11]. SnRK2 kinases phosphorylate and activate a faimly of basic leucine zipper (bZIP) transcription factors called ABFs/AREBs, which include ABA INSENSITIVE 3 (ABI3), ABI4 and ABI5, as well ascertain ion channels and transporter proteins [12–15]. The ABFs bind to ABA-responsive promoter elements (ABRE) to induce the expression of ABA-inducible genes and thereby control seed germination and seedling development [5].

ELONGATED HYPOCOTYL 5 (HY5), a bZIP transcription factor, is the primary regulator of light signaling pathways in plants [16, 17]. HY5 functions downstream of phytochromes, cryptochromes, and UV-B photoreceptors to mediate photomorphogenesis under red, blue, far-red, and UV-B light [18–24]. Recent studies have revealed that HY5 is also involved in ABA signaling ABA and abiotic stress responses [18, 25–29]. HY5 plays regulatory roles in responses to ABA and NaCl during seed germination and seedling growth [27, 28]. ABA- and salinity-promoted *ABI5* expression are both dependent on the presence of *HY5* [27, 28]. Upon salinity stress, HY5-interacting protein COP1 is translocated to the cytosol to avoid destroying nucleus-localized HY5, thereby facilitating *ABI5* expression [28]. In the mechanism underlying HY5 regulation of *ABI5*, HY5 may directly bind to the promoter of *ABI5* to increase the expression of *ABI5* and ABI5 target genes [27]. In addition, ABI5 can bind to its own promoter to promote its expression, while BBX21 negatively regulates *ABI5* expression by interfering with HY5 binding to the *ABI5* promoter [30]. In addition to its involvement in salinity stress responses, HY5 also regulates plant responses to cold stress and promotes the transcription of chilling responsive anthocyanin synthesis genes [29]. In this regard, HY5 can be an integrator of light signaling, ABA signaling and stress signaling [27]. *HY5-HOMOLOG* (*HYH*, *AT3G17609*), the closest homolog of *HY5* in the *Arabidopsis* genome [31], also encodes a bZIP transcription factor, which plays a role in the phyB signaling pathway. HY5 and HYH may act together to regulate the expression of their target genes and thus mediate many important cellular processes [31].

The MYB family, one of the largest families of transcription factors in *Arabidopsis*, includes approximately 200 genes [32] with a highly conserved DNA-binding domain (MYB domain). MYB proteins are involved in many processes, including metabolism, cell fate and identity, developmental processes and responses to biotic and abiotic stresses [33]. *Arabidopsis* RSM1 (RADIALIS-LIKE SANT/MYB 1), encoded by *At2g21650*, belongs to the MYB-related subfamily of the MYB family [34]. RSM1 has other names, MEE3 (MATERNAL EFFECT EMBRYO ARREST 3) [35] or AtRL2 (ARABIDOPSIS RAD-LIKE 2) [36]. Mutation of the *RSM1* gene affects female gametophyte development and embryogenesis in *Arabidopsis* [37]. RSM1, containing a SANT/MYB DNA-binding domain, is highly homologous to RADIALIS of *Antirrhinum majus* [38, 39]. Thus, RSM1 and its homologs in *Arabidopsis* were also designated the RAD-like family, which consists of four members: RADIALIS-LIKE SANT/MYB 1 (RSM1) (At2g21650), RSM2 (At4g39250), RSM3 (At1g75250) and RSM4 (At1g19510) [34]. *RSM1*-overexpressing seedlings are hookless and defective in gravitropism in the dark, while they display short hypocotyls under red light [34], indicating that *RSM1* is involved in seedling photomorphogenesis. A previous work from our laboratory demonstrated that MEE3/RSM1 is a novel repressor of the floral transition by activating transcription of *Flowering Locus C* (*FLC*), a key flowering repressor [40]. Additionally, a transcriptome analysis suggested that *RSM1* is highly expressed in guard cells and regulated by ABA and cold stress [41–44]. Moreover, transcription of *RSM1* was found to be up-regulated 2 hours after treatment with cytokinin BA [45]. Therefore, RSM1 may play roles in plant development, stress responses and plant hormone signaling, but the molecular mechanisms underlying the roles of *RSM1* in these processes remain obscure.

In our initial observations, *RSM1*-overexpressing plants exhibited opposite phenotypes to those of the mutants for *HY5/HYH* and shared similar phenotypes with those of *HY5*-overexpressing plants, with regard to seed germination, abiotic stress and ABA responses, seedling photomorphogenesis and the floral transition. These finding inspired us to speculate that the functions of RSM1 may be closely related to HY5/HYH. Questions arose regarding whether and how RSM1 and HY5/HYH are functionally associated in the biological processes listed above, but especially with regard to stress, ABA and light signaling pathways. In this work, we aimed to characterize the roles of RSM1 and homologs in plant responses to ABA and salinity during seed germination and seedling development, as well as to elucidate the relationships of RSM1 and homologs with HY5/HYH and ABA signaling components ABI5, ABI3, and ABI4. Our data suggest that RSM1 interacts with HY5/HYH to regulate responses of seed germination and seedling development to ABA and salinity in *Arabidopsis*.

## Results

### *RSM1* expression is regulated by ABA and NaCl

**Fig 1:**
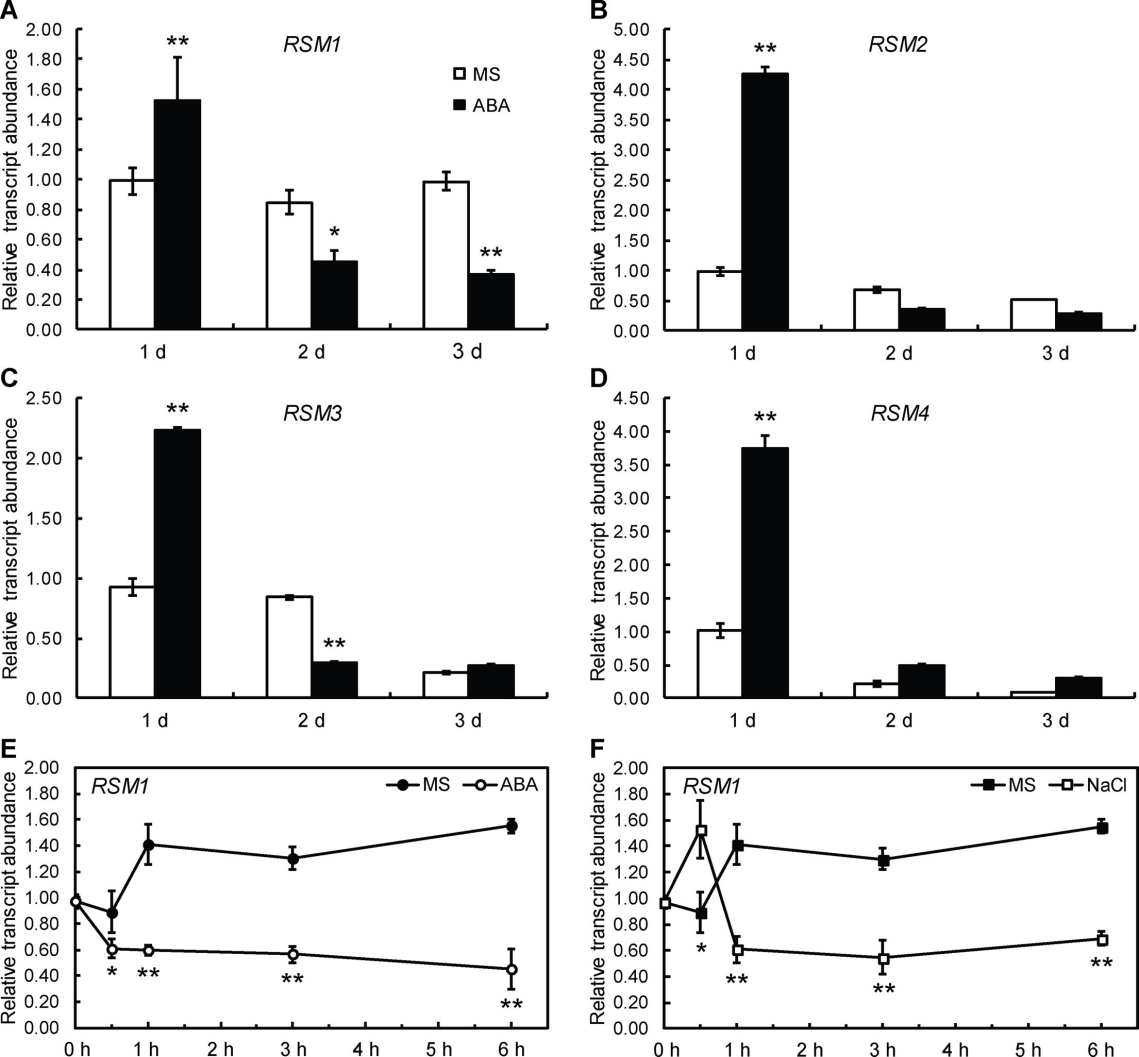
Expression of *RSM1* is regulated by ABA and NaCl during seed germination and seedling development. (A) to (D) Expression of *RSM1* (A), *RSM2* (B), *RSM3* (C) and *RSM4* (D) in germinating Col-0 wild-type seeds treated with or without ABA. Surface-sterilized WT seeds were incubated at 4°C in darkness for 3 days, and grown on MS medium supplemented with 0.2 μM ABA for another 1 day, 2 days or 3 days before harvested for RNA extraction and qRT-PCR analyses. *p<0.05 and **p<0.01 represent the significance of differences between each genotype and Col-0 at different times. (E), (F) Expression of *RSM1* in WT seedlings treated with 100 μM ABA or 200 mM NaCl at different time points. Total RNA was isolated from 7-day-old seedlings after the stress treatments. *ACTIN2* (*ACT2*) was used as a control for data normalization. Three independent replicates of measurements were performed for each time point, and the data are shown as the mean ± standard deviation (SD) (n = 3).

To explore the effect of ABA and salt stress, quantitative RT-PCR (qRT-PCR) was conducted with imbibed seeds and seedlings. As shown in Fig 1A, *RSM1* expression in imbibed seeds exposed to light was induced at one day of ABA treatment and subsequently repressed. *RSM1* has three homologs: *RSM2* (*At4g39250*), *RSM3* (*At1g75250*) and *RSM4* (*At1g19510*) [34]. The amino acid sequences of RSM2, RSM3, and RSM4 are highly similar to that of RSM1, with identities of 73.96%, 70.13% and 68.83%, respectively (S1 Fig). The expression levels of *RSM2*, *RSM3* and *RSM4* were also regulated by ABA during seed germination and seedling development (Fig 1B–1D). Similarly, *RSM1* expression was also regulated by ABA and high salinity in 7-day-old seedlings of Col-0 wild-type (WT). As shown in Fig 1E, *RSM1* expression was inhibited by ABA treatment at multiple time points (Fig 1E). The level of *RSM1* expression was moderately induced at the beginning of treatment and then repressed compared to the initial level, when challenged with 200 mM NaCl (Fig 1F).

Taken together, the regulation patterns of *RSM1* expression shown above suggest that RSM1 is involved in ABA and salinity stress responses during seed germination and seedling development.

### Down-regulation or up-regulation of *RSM1* expression alters the sensitivity of seed germination and cotyledon greening to ABA, high salinity and osmotic stresses in *Arabidopsis*

Previous transcriptome analyses [42, 43] and our assays of *RSM1* expression (Fig 1) suggest that RSM1 may be involved in ABA and abiotic stress signaling.

Therefore, seed germination and root elongation were measured for *RSM1*-related genetic materials treated with or without ABA, NaCl or mannitol at different concentrations. T-DNA insertion mutants of *RSM1* (*CS876657*), *RSM2* (*CS371942*) and *RSM3* (*Salk_069941C*) were obtained from ABRC, whereas *rsm1 rsm2* double mutants and *rsm1 rsm2 rsm3* triple mutants were generated in our laboratory (S1 Fig), and qRT-PCR was used to measure the *RSM1* expression level of each mutant using technique. As shown in S1E Fig, *RSM1* expression was reduced in the *rsm1* mutant, *rsm1 rsm2* double mutant and *rsm1 rsm2 rsm3* triple mutant in comparison with that of the WT plants. Moreover, transgenic *RSM1*-overexpressing plants *OX-9* and *OX-12* [40] were also assessed in this study.

**Fig 2:**
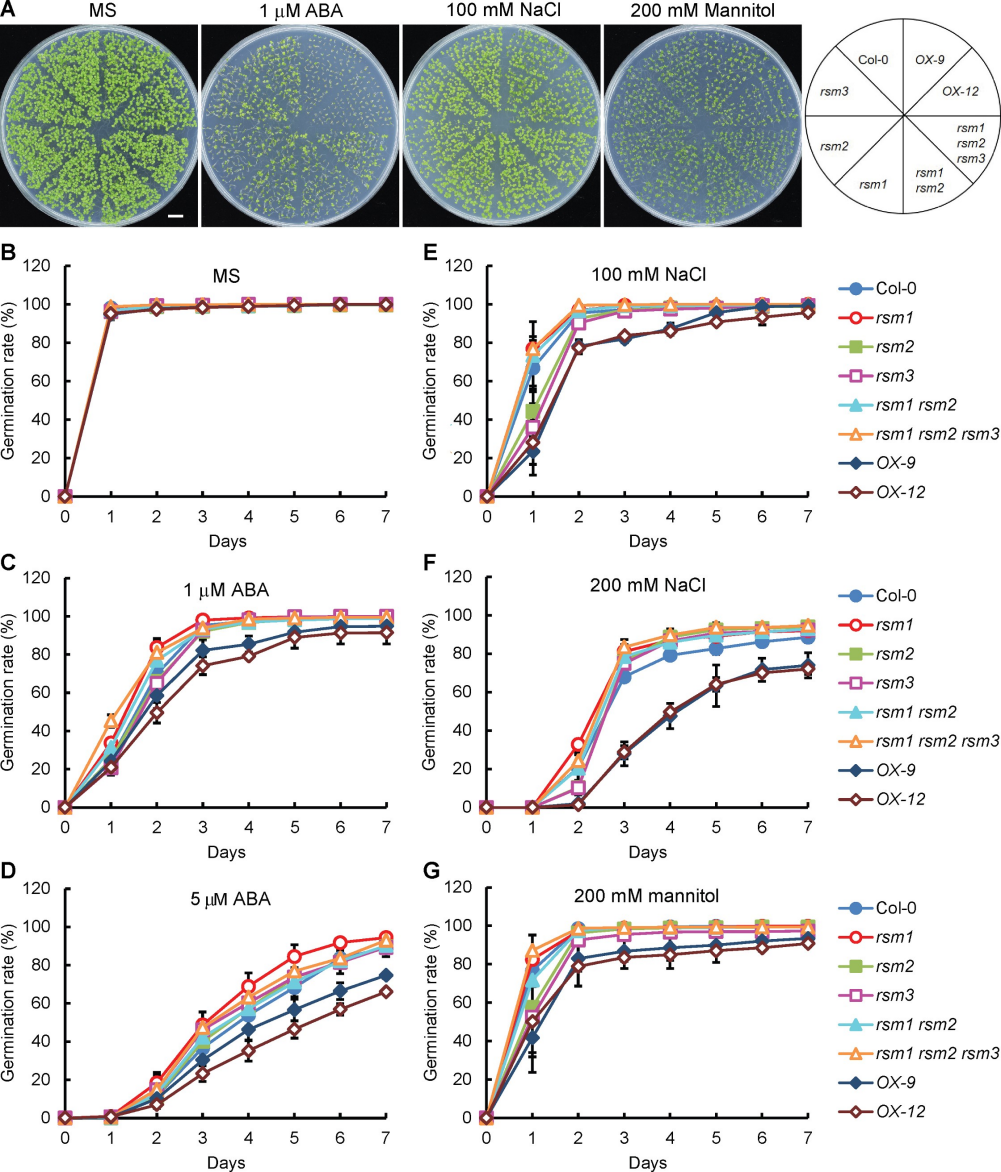
*RSM1* mutation or overexpression alters plant responses to ABA, NaCl and mannitol during seed germination. (A) Morphology of 7-d-old seedlings of Col-0, *rsm1*, *rsm2*, *rsm3*, *rsm1 rsm2*, *rsm1 rsm2 rsm3*, *OX-9* and *OX-12* grown on MS medium with or without 1 μM ABA, 100 mM NaCl or 200 mM mannitol. *rsm1* is a *RSM1* T-DNA insertion mutant, with an insertion at the 5 ′-UTR region of *At2g21650*. *OX-9* and *OX-12* are two independent lines of *RSM1-*overexpressing plants, in which transgene expression is driven by the *CaMV 35S promoter*. The scale bar indicates 1 cm. (B) to (D) Germination rates of *RSM1*-related genetic materials grown on MS medium supplemented with or without ABA at different concentrations (0, 1 and 5 μM). (E), (F) Germination rates of *RSM1*-related materials grown on MS medium supplemented with or without NaCl at different concentrations (100 or 200 mM). (G) Germination rates of *RSM1*-related materials grown on MS medium supplemented with or without 200 mM mannitol. Surface-sterilized seeds were sown on the respective plates and incubated at 4°C in darkness for 3 days before placed at 22°C under long-day condition (16 h day/ 8 h night) for germination. Germination rates were determined at the indicated time. The data are shown as the mean ± SD from three independent replicates (n = 3), where approximately 100 seeds were used per replicate per genotype.

In the absence of treatment, the germination rates of *RSM1*-related materials (including both mutants and overexpressing plants) showed no clear differences from that of the WT plants (Fig 2A and 2B). In contrast, overexpression of *RSM1* gave rise to increased sensitivity to ABA, NaCl and mannitol treatments, whereas the single mutant for *RSM1*, *rsm1*, exhibited reduced sensitivity of seed germination to the ABA and NaCl treatments to different extents (Fig 2C–2G). Interestingly, the single mutants *rsm2* and *rsm3* displayed no clear differences from the WT plants while *rsm3* was more sensitive to ABA, NaCl and mannitol earlier in the germination process (Fig 2C and 2E–2G). Additional information regarding the statistical analysis is included in S1 Table. With regard to cotyledon greening, there was no difference among the *RSM1*-related materials and the WT plants without treatment (S2A Fig). However, *RSM1*-overexpressing seeds were hypersensitive to ABA, NaCl and mannitol with regard to cotyledon greening, while the *rsm1 rsm2 rsm3* triple mutant displayed reduced sensitivity to 1 μM ABA, and the *rsm2* and *rsm3* single mutants were more sensitive to 100 mM NaCl earlier in the germination process, as compared to control plants (S2B–S2D Fig). Additional information regarding the statistical analysis is included in S2 Table.

To evaluate the responses of early seedling development and growth to stress treatments, we also measured the fresh weight of 7-day-old *RSM1*-related materials after stratification and treatment with or without 1 μM ABA, 100 mM NaCl or 200 mM mannitol. The fresh weights of *OX-9* and *OX-12* seedlings were significantly lower than those of other genotypes following treatments with 1 μM ABA or 200 mM mannitol, but they were moderately lower than those of other genotypes following treatment with 100 mM NaCl. However, only the fresh weight of the *rsm1* mutant seedlings displayed reduced sensitivity to treatment with 200 mM mannitol in comparison with that of the WT plants (S2E Fig).

In addition, we also determined whether *RSM1* is involved in inhibition of root elongation by ABA. Five-day-old seedlings were transferred to plates supplied with or without ABA, NaCl and mannitol, and the root length was measured 5 d after the transfer. In the absence of treatment, the primary root lengths of the *RSM1* mutants, *RSM1*-overexpressing plants, and control plants were similar, whereas the root length of the *RSM1-*overexpressing plants shorter than that of the WT plants under ABA treatment (S3 Fig). However, there were no clear differences among the the root lengths of any of the tested plants treated with NaCl or mannitol. These results suggest that RSM1 may be weakly involved in the regulation of primary root elongation by ABA.

### *RSM1* expression alters seedling tolerance to high salinity during seedling development

**Fig 3:**
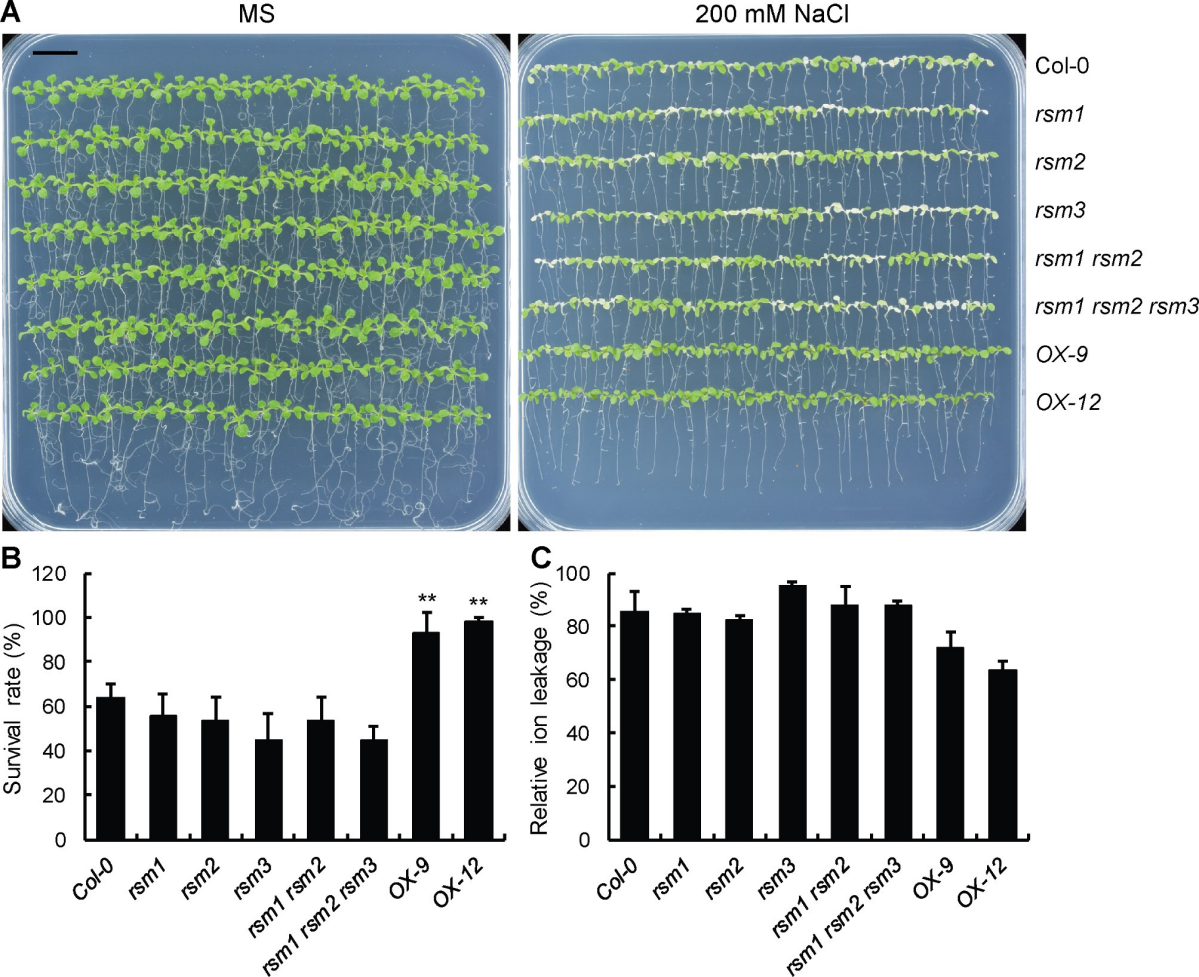
*RSM1* overexpression causes hyposensitivity to salinity during seedling development. (A) Overexpression of *RSM1* enhances salt tolerance. Plants were grown on MS medium for 7 days and transferred to MS medium with or without 200 mM NaCl for 3–4 days, after which they were photographed. The images are representative of at least three experimental replicates. The scale bar indicates 1 cm. (B) Survival rates of *RSM1*-related materials as illustrated in (A). The survival rates were determined by scoring and calculating the ratio of the number of bleached seedlings to the total number of seedlings. The data are shown as the mean ± SD of three independent replicates of measurements (n = 3), where each genotype replicate included approximately 25 seedlings. ** indicates p<0.01 for the significance of the difference between each genotype and Col-0. (C) Relative ion leakage of *RSM1*-related seedlings as shown in (A). The data are shown as the mean ± SD from three replicates, each of which included approximately 50 seedlings (n = 3).

The potential role of RSM1 in seedling responses to salinity stress was also assessed by examining the survival rates of various genotypes under high-salinity conditions. Seven-day-old seedlings were transferred to plates with or without 200 mM NaCl supplementation before measurements were collected. All seedlings grew well under the control conditions (Fig 3A), and only *RSM1-*overexpressing plants (*OX-9* and *OX-12* lines) exhibited a higher rate of survival than that of the WT plants, which suggests that *RSM1* overexpression enhances the tolerance of seedlings to high salinity (Fig 3A and 3B). In addition, single mutant *rsm3* and the triple mutant displayed reduced survival rates in comparison with that of the WT plants (Fig 3A and 3B). In concordance with the survival rate assay, *RSM1-*overexpression resulted in reduced relative electrolyte leakage in comparison with that of the WT plants, as revealed in electrolyte leakage assay (Fig 3C). However, the single, double, and triple mutants displayed no obvious differences in ion leakage in comparison with the WT plants (Fig 3C). Intracellular K^+^/Na^+^ homeostasis is important for plants responding to salt stress and adaption. K^+^/Na^+^ assays showed that salt-stressed *RSM1*-overexpressing seedlings accumulated more K^+^ and less Na^+^, whereas the mutants showed no evident differences in comparison with the WT plants (S4 Fig). These results suggest that RSM1 may enhance the tolerance of plants to high salinity during seedling development.

### Organ specificity and cellular localization of *RSM1*

To analyze the organ specificity of the expression patterns of *RSM1*, we generated a *proRSM1*:*GUS* construct consisting of a 2.4-kb fragment of the *RSM1* promoter to drive the *GUS* reporter gene. The *proRSM1*:*GUS* construct was transformed into the Col-0 wild-type background. *RSM1* promoter activity was not detected in dry seeds (S5A Fig), but it was detected in all other tested organs, including the cotyledons, hypocotyls, radicles, true leaves and roots of young seedlings (S5B–S5H Fig), as well as rosette leaves (S5I Fig), floral organs (S5J and S5K Fig) and developing siliques (S5L Fig).*RSM1* promoter activity was mainly observed in the vascular tissues of several organs (S5E–S5H Fig).

*proRSM1*:*GFP-RSM1* plants were initially used to determine the cellular localization of RSM1, but possibly due to the low expression level of RSM1, no apparent signal was detected. Therefore, *35S*:*GFP-RSM1* lines were used to determine the cellular localization of RSM1. As shown in S5M Fig, GFP-RSM1 protein was localized in the nucleus and in the vicinity of the plasma membrane of epidermal cells in the cotyledons, hypocotyls, and roots of 5-day-old seedlings. GFP-RSM1 protein also localized in guard cells and the vascular tissues of the true leaves of seedlings (S6A Fig). These findings were in agreement with previous results from our laboratory [40]. In addition, we performed transactivation assay for RSM1 in yeast cells. As illustrated in S6B Fig, RSM1 did not exhibit transactivation activity despite confirmed expression in yeast (S6C Fig). However, the results of the cellular localization assays and bioinformatics analysis suggest that RSM1 is likely to function as a possible transcription factor.

### RSM1 regulates ABA signaling and binds to the promoter of *ABI5* to enhance its expression

**Fig 4:**
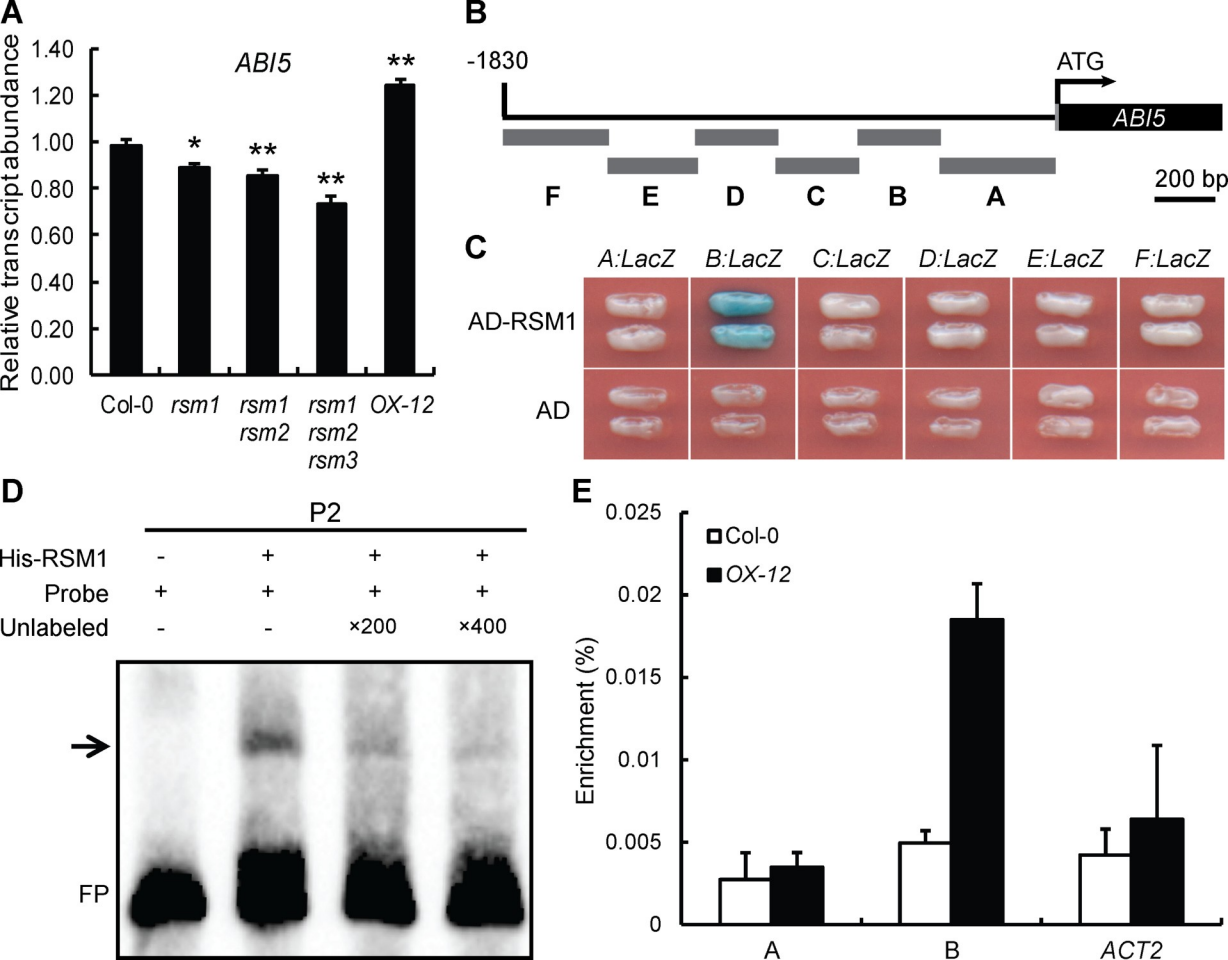
RSM1 binds to the promoter of *ABI5* to regulate its expression. (A) Expression of *ABI5* in 1-day-old germinating seeds (Col-0, *rsm1*, *rsm1 rsm2*, *rsm1 rsm2 rsm3* and *OX-12*) grown on MS medium as determined by qRT-PCR. The relative transcript levels were normalized to the abundance of reference gene *ACT2* and shown as the means ± SD of three replicates (n = 3). The data are shown as the mean ± SD of three replicates per experiment (* p<0.05 and ** p<0.01). (B) Diagram of the *ABI5* promoter fragments used to drive *LacZ* reporter gene expression in the yeast one-hybrid assays shown in (C). (C) Yeast one-hybrid assay, showing that RSM1 binds to fragment B of the *ABI5* promoter. *EGY48* cells were co-transformed with *pB42AD-RSM1* and the *pLacZ2U-ABI5* promoter. *pB42AD* was used as a negative control. (D) EMSAs showing that RSM1 binds to subfragment P2 of the *ABI5* promoter. The symbols “+” and “−” indicate presence or absence respectively, of the reagent shown at the top left of the panel. FP, free probe. (E) ChIP-qPCR to assess RSM1 binding to the *ABI5* promoter. Twelve-day-old WT and *OX-12* seedlings were harvested for ChIP-qPCR assays using anti-RSM1 polyclonal antibody to immunoprecipitate RSM1 and its associated genomic DNA segments. The enrichment (%) was normalized to the level of input DNA. *ACT2* and fragment A of the *ABI5* promoter were used as negative controls. The data are shown as the mean ± SD from three independent replicate measurements (n = 3).

To determine the mechanism by which RSM1 mediates ABA signaling, the expression levels of essential and marker genes in ABA signaling were analyzed in various *RSM1*-related genotypes during seed germination with or without ABA treatment. *ABI5*, *RD29A*, *RD29B*, *AtEM1*, *AtEM6*, *RAB18*, *ABF3* and *ABF4* were overall down-regulated in *rsm1*, *rsm1 rsm2* and *rsm1 rsm2 rsm3* germinating seeds in the absence or presence of ABA (Fig 4A and S7 Fig). Most of the tested genes were responsive to ABA (S7 Fig) [12, 13, 46–48]. The expression levels of genes upstream of the ABA signaling pathway, including *ABI1*, *ABI2*, *SnRK2*.*2* and *SnRK2*.*3* were not found to be apparently regulated by *RSM1* (S7 Fig). Based on these observations, we speculate that RSM1 may function by regulating ABA and stress signaling at a particular node such as *ABI5*, while RSM1 does not seem to regulate major upstream ABA signaling components such as PP2Cs and SnRK2.2s.

To test whether RSM1 regulates *ABI5* by binding to its promoter, yeast one-hybrid assays were performed using the 1800 bp genomic sequence before the start codon of the *ABI5* gene. The *ABI5* promoter was divided into six fragments (A, B, C, D, E and F), which were cloned into yeast one-hybrid reporter constructs to drive expression of the *LacZ* reporter gene (Fig 4B). As shown in Fig 4C, RSM1 bound only to fragment B, which contained the sequence extending from -703 to -375 bp before the start codon of the *ABI5* gene. To map the specific RSM1 binding sites on the *ABI5* promoter, fragment B was divided into six short fragments (approximately 60 bp) for electrophoretic mobility shift assays (EMSA). As shown in Fig 4D, the His-RSM1 protein was able to associate with fragment P2 (-648 to -593 bp) of the *ABI5* promoter (Fig 4D). Chromatin immunoprecipitation (ChIP) assays were performed with the material from 12-day-old *RSM1*-overexpressing plants and the WT plants to determine whether RSM1 binds to the *ABI5* promoter *in vivo*. The ChIP assays confirmed that RSM1 associated with fragment B, but not with the control sequence (*ACTIN2*) or fragment A, *in vivo* (Fig 4E). Taken together, these findings demonstrate that RSM1 may function as a transcription factor by binding to the *ABI5* promoter to regulate *ABI5* expression and thus influence the ABA signaling pathway.

### *ABI5* is downstream of *RSM1* in the processes of ABA-regulated seed germination and post-germination

**Fig 5:**
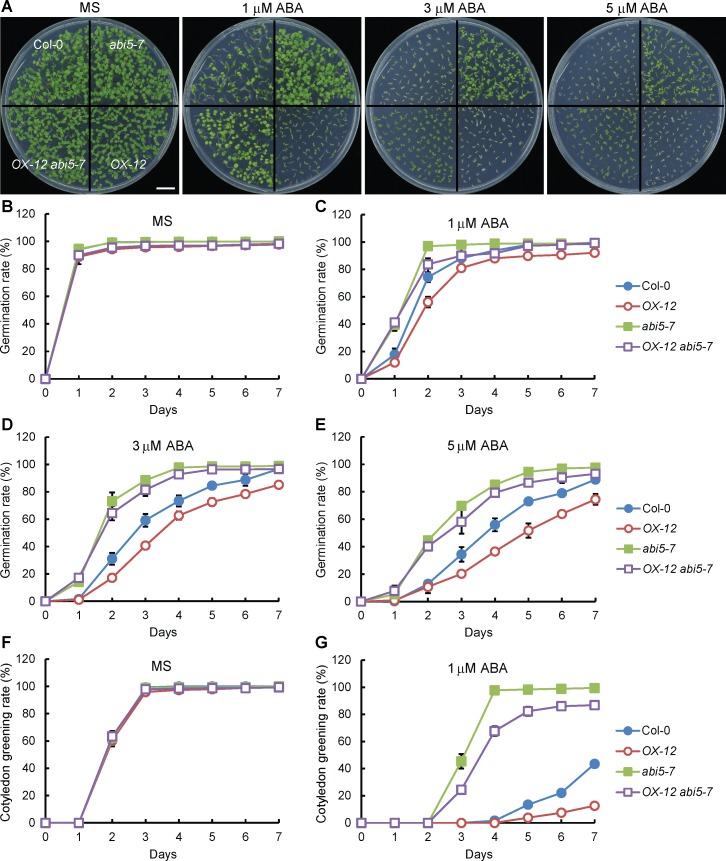
*ABI5* is epistatic to *RSM1* in ABA regulation of seed germination and post-germination. (A) Morphology of 7-d-old seedlings (Col-0, *abi5-7*, *OX-12* and *OX-12 abi5-7*) grown on plates with or without 1, 3 or 5 μM ABA. The scale bar indicates 1 cm. (B) to (E) Germination rates of Col-0, *abi5-7*, *OX-12* and *OX-12 abi5-7* seeds grown under treatment with different concentrations of ABA (0, 1, 3 and 5 μM). (F), (G) Cotyledon greening rates of Col-0, *abi5-7*, *OX-12* and *OX-12 abi5-7* seedlings grown under treatment with different concentrations of ABA (0 and 1 μM). The germination rates and cotyledon greening rates were scored and calculated at the indicated time. The data are shown as the mean ± SD from three independent experimental replicates (n = 3). Approximately 100 seeds were used per genotype replicate.

The findings described above prompted to address whether *RSM1* and *ABI5* interact genetically in ABA signaling. To this end, an *RSM1* overexpressing line (*OX-12*) was crossed with the *abi5-7* mutant. The germination rates of *abi5-7*, *OX-12* and *OX-12 abi5-7* were similar to that of the WT plants in the absence of ABA (Fig 5A and 5B). However, in the presence of ABA, the germination rate of *OX-12* was lower than that of the WT plants, whereas that of *abi5-7* was higher than that of the WT plants. The differential ABA responses of *OX-12* and *abi5-7* seeds were more evident at higher concentrations of ABA. *OX-12 abi5-7* was similarly less sensitive to ABA (Fig 5C–5E). The cotyledon greening rates of *OX-12 abi5-7*, *OX-12*, and *abi5-7* were similar to that of the WT plants without ABA (Fig 5F). In the presence of ABA, *OX-12* was more sensitive and *abi5-7* was less sensitive to ABA regarding cotyledon greening, whereas *OX-12 abi5-7* was similarly less sensitive to ABA as compared to *abi5-7* (Fig 5G). Additional information regarding the statistical analysis is included in S3 Table. These findings suggest that *ABI5* is downstream of *RSM1* in the processes of seed germination and seedling development.

In addition to ABI5, ABI3 and ABI4 are also vital positive regulators of ABA signaling during seed germination and seedling development [14, 15, 49]. To analyze the genetic relationship of *RSM1* with *ABI3* and *ABI4*, *OX-12* was crossed with *abi3-8* or *abi4-1*. The seed germination rates and cotyledon greening rates of various genotypes were examined under varying concentrations of ABA (0, 1 μM and 5 μM). In the absence of ABA, no clear differences in the seed germination rate or cotyledon greening rate were observed among the tested genotypes (S8A, S8B and S8F Fig). However, in the presence of ABA, the germination rate and cotyledon greening rate of *OX-12* were lower than those of the WT plants, whereas those of *abi3-8* were higher than those of the WT plants. The responses of *OX-12 abi3-8* and *abi3-8* to the presence of ABA were similar (S8C, S8D, S8G and S8H Fig). Additional information regarding the statistical analysis is included in S4 Table.

The fresh weights of 7-day-old seedlings were measured after stratification and treatment with or without ABA (S8E Fig). The fresh weight of *OX-12* seedlings was significantly lower than those of the seedlings of other genotypes under 1 μM ABA treatment, but *OX-12 abi3-8* and *abi3-8* both showed greater fresh weight under ABA treatment, displaying reduced sensitivity to ABA treatments (S8E Fig). These results suggest that *ABI3* is downstream of *RSM1* during seed germination and seedling development. Similarly, without ABA, the seed germination rates and cotyledon greening rates of the control, *abi4-1*, *OX-12* and *OX-12 abi4-1* plants were similar (S9A, S9B and S9F Fig). However, in the presence of ABA, the germination rate and cotyledon greening rate were lower in *OX-12* but higher in *abi4-1* as compared to those of the WT plants, while *OX-12 abi4-1* and *abi4-1* were similarly less sensitive to ABA (S9C–S9E and S9G Fig). Additional information regarding the statistical analysis is included in S5 Table.

The results described above suggest that *ABI3*, *ABI4* and *ABI5* are downstream of *RSM1* to mediate the function of RSM1 in ABA-regulation of seed germination and post-germination.

### *RSM1* is regulated by HY5/HYH

Based on initial experiments as discussed earlier, we postulated that RSM1 and HY5/HYH may have a close relationship at the transcriptional regulation and/or protein interaction level.

**Fig 6:**
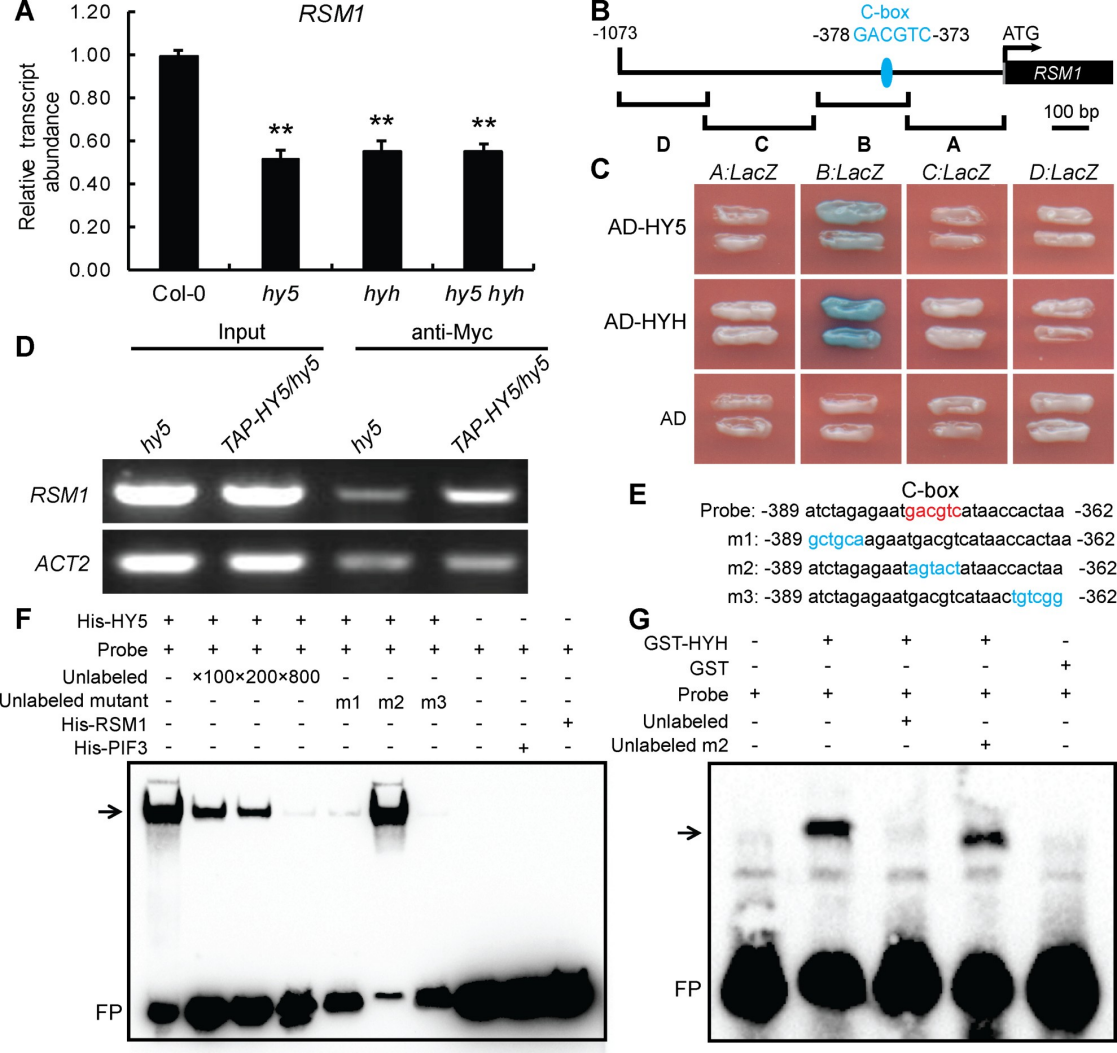
HY5 and HYH regulate *RSM1* expression by binding to the *RSM1* promoter. (A) Comparison of the expression levels of *RSM1* in 7-day-old Col-0, *hy5*, *hyh* and *hy5 hyh* seedlings. *PP2A* was used as a control for data normalization. The data are shown as the mean ± SD from three independent experimental replicates (n = 3). **p < 0.01 indicates the significance of the differences as compared to Col-0. (B) Diagram of the *RSM1* promoter fragments used to drive *LacZ* reporter gene expression in the yeast one-hybrid assay shown in (C). (C) Yeast-one hybrid assay showing that HY5 and HYH bind to fragment B of the *RSM1* promoter. *EGY48* cells were co-transformed with pB42AD-HY5/HYH and the *pLacZ2U-RSM1* promoter. (D) ChIP-qPCR to assess HY5 binding to the *RSM1* promoter *in vivo*. Twelve-day-old *hy5* and *35S*:*TAP-HY5/hy5* seedlings were harvested for ChIP-qPCR assays using anti-Myc antibody to immunoprecipitate genomic DNA segments. The input was genomic DNA that was not subjected to immunoprecipitation. *ACT2* was used as a negative control. (E) Diagram of the WT version of the C-box-contained biotin-labeled DNA probe and various biotin-unlabeled mutant versions of the *RSM1* promoter subfragments used in the EMSAs shown in (F) and (G). The C-box element in the WT DNA probe is shown in red, whereas nucleotide substitutions in the mutated subfragments are shown in blue. (F) EMSAs of HY5 binding to the *RSM1* promoter *in vitro*. The biotin-labeled DNA probe was incubated with His-HY5 protein. His-PIF3 and His-RSM1 were used as negative protein controls. (G) EMSAs of HYH binding to the *RSM1* promoter *in vitro*. The biotin-labeled DNA probe was incubated with GST-HYH protein. GST was used as the negative protein control. In (F) and (G), the symbols “+” and “−” indicate the presence or absence, respectively, of the reagent that is indicated at the the top left of the panel. FP, free probe.

To test whether HY5/HYH regulate the expression of *RSM1*, qRT-PCR was employed to measure the transcript levels of *RSM1* in HY5/HYH-related genetic materials. As shown in Fig 6A, the transcript level of *RSM1* was significantly down-regulated in *hy5*, *hyh* and *hy5 hyh* mutants as compared to that of the WT plants. Although transcription of *RSM1* is regulated by HY5/HYH, it was unclear whether HY5 and HYH regulate transcription of *RSM1* by binding to its promoter. By analyzing the promoter of *RSM1* using an online tool (http://www.softberry.com), we identified a C-box sequence (GACGTC) located between -378 bp and -373 bp in the promoter region of *RSM1*, which has been predicted and experimentally demonstrated to be a HY5-binding site [50]. To determine how HY5/HYH binds to the *RSM1* promoter, an approximately 1100 bp genomic sequence before the start codon of the *RSM1* gene was divided it into four fragments, which were utilized in yeast one-hybrid assays (Fig 6B). HY5 and HYH were found to bind specifically to the sequence located between -523 bp and -268 bp in fragment B of the *RSM1* promoter, which included the C-box mentioned above (Fig 6C). ChIP and EMSA were performed to obtain further confirmation of this interaction. As shown in Fig 6D, ChIP-qPCR revealed that HY5 could bind to the *RSM1* promoter *in vivo*. In the EMSA assay, three mutated probe sequences, designated as m1, m2 and m3, were designed as unlabeled competitive probes to test the specificity of binding sites on the *RSM1* promoter. As shown in Fig 6E–6G, the binding of HY5 and HYH to the C-box-containing sequence was effectively competed by unlabeled probes m1 and m3, which contained a wild-type C-box but not by probe m2, in which the C-box was mutated. Therefore, three different approaches demonstrate that HY5 and HYH specifically bind to the *RSM1* promoter, which may facilitate their regulation of the activity of the *RSM1* promoter.

Next, the effect of HY5 on *RSM1* promoter activity was visualized using GUS reporter gene analyses. *RSM1* promoter activity was detected in the cotyledons and hypocotyls of 3-day-old seedlings (S10A Fig) In cotyledons, *RSM1* promoter activity was mainly detected in vascular tissues. Visually, *proRSM1*:*GUS* activity was notably decreased in the *hy5* mutant background (S10B and S10C Fig) as compared to that of the wild-type background (S10A Fig), which suggested that the *hy5* mutation reduced *RSM1* promoter activity. These findings support the notion that regulation of *RSM1* transcription may require the presence of HY5.

### *RSM1* mediates the functions of *HY5*/*HYH* in ABA-regulated seed germination and the response to high salinity during seedling development

**Fig 7:**
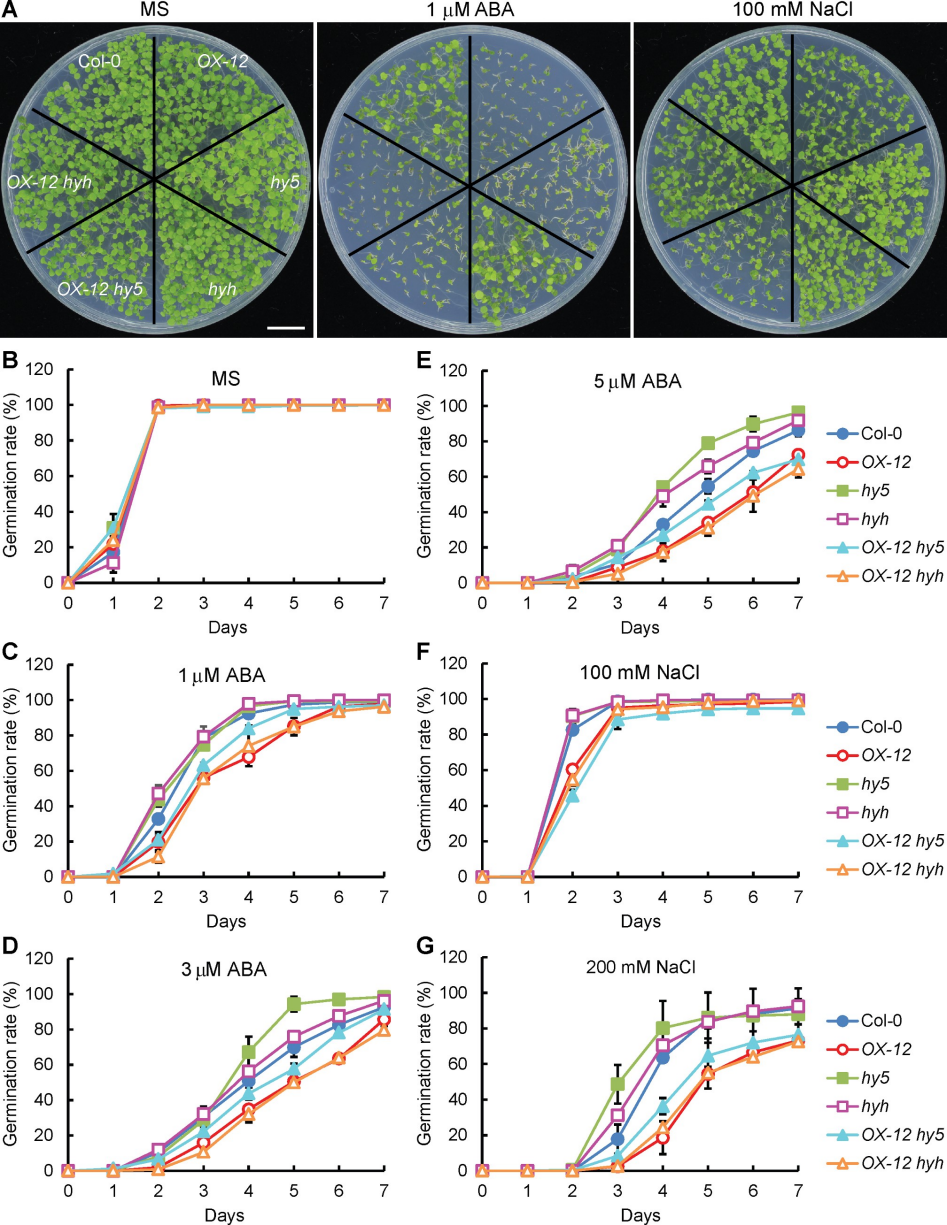
*RSM1* is downstream of *HY5* and *HYH* responses of seed germination to ABA or NaCl. (A) Morphology of 7-day-old seedlings (Col-0, *hy5*, *hyh*, *OX-12*, *OX-12 hy5* and *OX-12 hyh*) grown on plates without or with 1 μM ABA and 100 mM NaCl. The scale bar indicates 1 cm. (B) to (E) Germination rates of Col-0, *hy5*, *hyh*, *OX-12*, *OX-12 hy5* and *OX-12 hyh* seeds grown under treatment with different concentrations of ABA (0, 1, 3 and 5 μM). (F) to (G) Germination rates of Col-0, *hy5*, *hyh*, *OX-12*, *OX-12 hy5* and *OX-12 hyh* seeds grown under treatment with different concentrations of NaCl (100 and 200 mM). Germination rates were scored and calculated at the indicated time. The data are shown as the mean ± SD from three independent experimental replicates. Approximately 100 seeds were used per genotype replicate.

As a key positive regulator, HY5 plays an essential role in light signaling [25] in addition to its roles in the regulation of plant development and stress responses [27, 29, 30]. *hy5* mutant plants exhibit hyposensitivity to ABA and salt treatments during seed germination and seedling growth [27]. As stated earlier, *RSM1*-related genetic materials displayed phenotypes relevant to those of *hy5* mutants (Figs 2 and 3), which suggested that HY5 and RSM1 might be functionally related in the contexts of seed germination and seedling growth. To address this question, *OX-12 was* crossed with *hy5*, *hyh* or *hy5 hyh*, and phenotypical analyses were performed on the resulting plants. Wild-type, *hy5*, *hyh*, *OX-12*, *OX-12 hy5* and *OX-12 hyh* exhibited similar germination rates on MS medium (Fig 7A and 7B). With ABA or NaCl supplementation, the germination rates of *hy5* and *hyh* were much higher than those of the WT plants and the other tested genotypes. However, the germination rate of *OX-12 hy5* was remarkably repressed relative to that of the WT plants, which mimicked the phenotype of *OX-12* (Fig 7C–7G). Additional information regarding the statistical analysis is available in S6 Table. These results suggest that *RSM1* is downstream of *HY5*/*HYH* and thus mediates the functions of both genes in the regulation of seed germination by ABA and salt stress.

**Fig 8:**
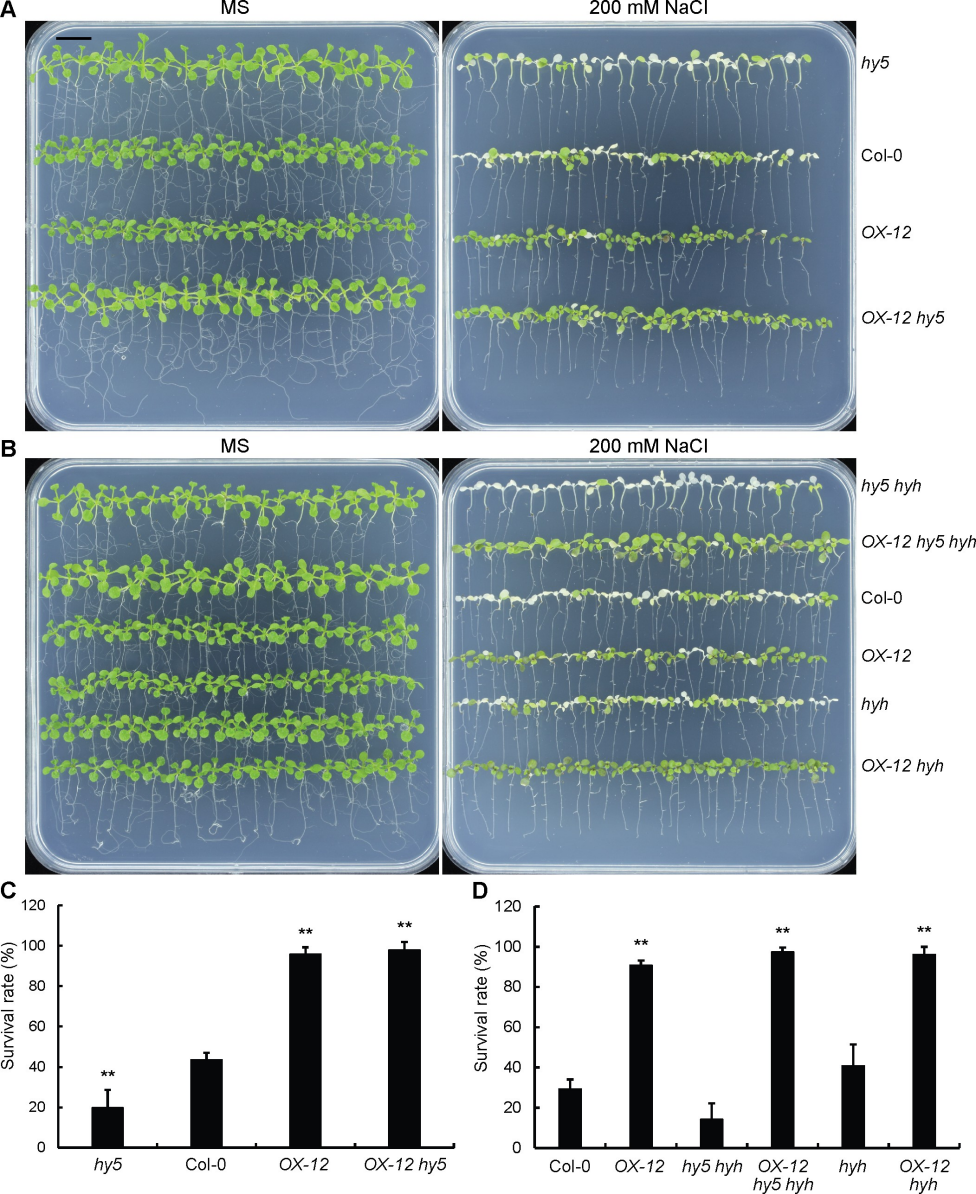
*RSM1* is downstream of *HY5*/*HYH* in tolerance to high salinity during seedling development. (A) Morphology of Col-0, *OX-12*, *hyh*, *hy5 hyh*, *OX-12 hyh* and *OX-12 hy5 hyh* seedlings grown on plates with or without 200 mM NaCl. (B) Morphology of WT, *OX-12*, *hyh*, *hy5 hyh*, *OX-12 hyh* and *OX-12 hy5 hyh* seedlings grown on MS plates with or without 200 mM NaCl. Seven-day-old seedlings grown on MS medium were transferred to MS media supplemented with or without 200 mM NaCl. The images were taken 3–4 days after the transfer. The scale bar indicates 1 cm for (A) and (B). (C) Survival rates of Col-0, *OX-12*, *hy5* and *OX-12 hy5* seedlings as shown in (A). (D) Survival rates of Col-0, *OX-12*, *hyh*, *hy5 hyh*, *OX-12 hyh* and *OX-12 hy5 hyh* seedlings as shown in (B). Survival rates were determined by calculating the ratio of the number of bleached seedlings to the total number of seedlings. The data are shown as the mean ± SD of three independent experimental replicates (n = 3), each of which included approximately 25 seedlings. ** indicates p<0.01 for the significance of difference between each genotype and Col-0.

In addition to seed germination, we also tested the tolerance of different genotypes to salt treatment to determine whether *RSM1* and *HY5/HYH* had the same relationship as that revealed for the effects of NaCl on seed germination. To this end, we calculated the survival rates (rates of non-bleached seedlings) of different genotypes after 7-day-old seedlings were transferred to MS medium supplemented with 200 mM NaCl for 3 days. Surprisingly, *hy5*, *hyh* and *hy5 hyh* were sensitive to the NaCl treatment, whereas *OX-12* was tolerant to the NaCl treatment in this assay (Fig 8A and 8B), although *hy5* was less sensitive whereas *OX-12* was sensitive to NaCl treatment in the germination assay. Apparently, *OX-12 hy5*, *OX-12 hyh* and *OX-12 hy5 hyh* mimicked *OX-12* with regard to survival rate (Fig 8C and 8D), which indicated that *RSM1* is also downstream of *HY5*/*HYH* in seedling responses to salinity stress.

These findings suggest that *RSM1* is downstream of *HY5*/*HYH* in the responses of plants to high salinity in the germination and seedling developmental stages, although it seems that these genes/proteins play opposite roles in each stage.

### RSM1 interacts with HY5 and HYH both *in vitro* and *in vivo*, and enhances HY5 binding to the *ABI5* promoter

**Fig 9:**
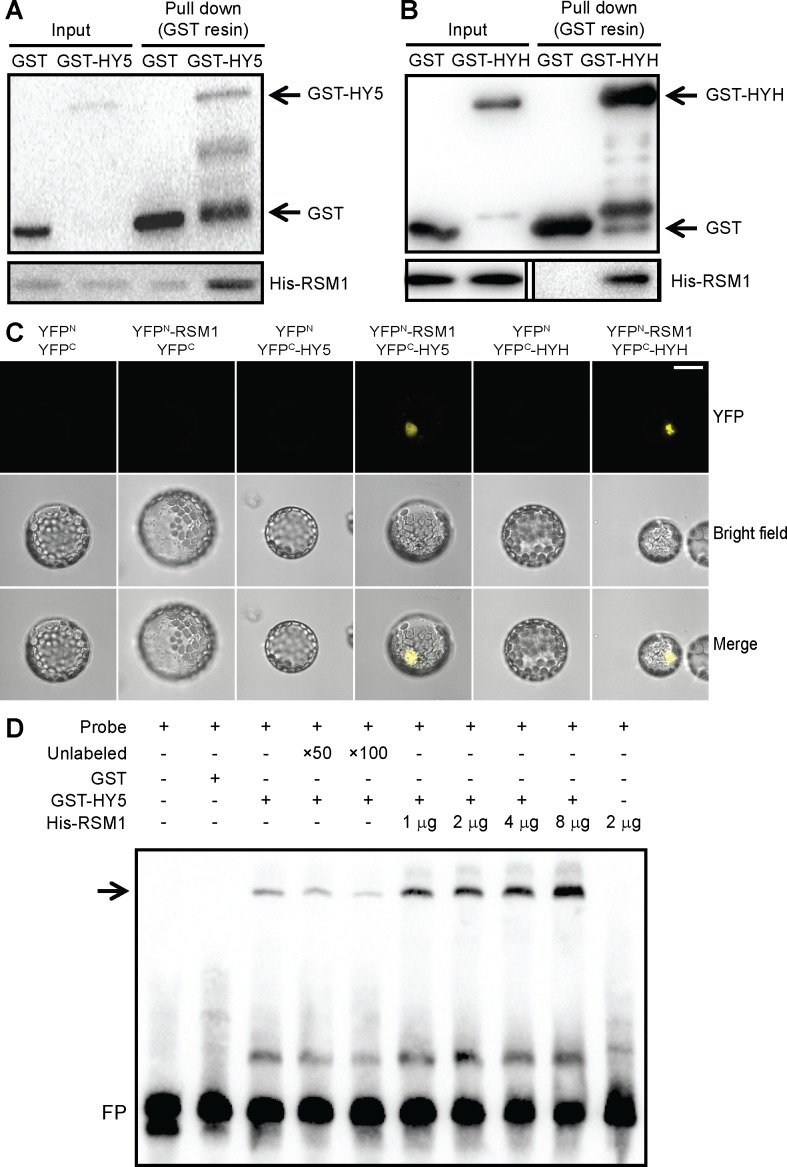
RSM1 interacts with HY5 and HYH *in vitro* and in *vivo*. (A) GST-HY5 and (B) GST-HYH interacted with His-RSM1 *in vitro* in the pull-down assay. His-RSM1 was incubated with GST or GST-HY5 (or GST-HYH) and glutathione beads at 4°C for 2 hours, after which the precipitates were subjected to SDS PAGE and immunoblotting with anti-GST or anti-His antibody as the primary antibody. (C) RSM1 interacted with HY5 and HYH *in vivo* in BiFC assays in protoplasts. YFP^N^-RSM1 and YFP^C^-HY5/HYH were transiently co-transformed into *Arabidopsis* mesophyll cell protoplasts. After overnight incubation in the dark, the YFP signal was detected by confocal microscopy. Pairs of empty vectors (YFP^N^ and YFP^C^) and a protein fusion vector were used as negative controls. The scale bar indicates 10 μM. (D) RSM1 affected HY5 binding to the *ABI5* promoter in EMSAs. The biotin-labeled DNA probe was incubated with GST-HY5 or His-RSM1 protein. GST served as the negative protein control. Two grams of either GST or GST-HY5 protein were added to the mixture, while His-RSM1 protein was added as indicated in the figure. The symbosl “+” and “−” indicate the presence or absence, respectively, of the reagent indicated atg the the top left of the panel. FP, free probe.

Considering that RSM1 interacts with HY5 and HYH genetically, we questioned whether they also physically interact. To address this question, we carried out *in vitro* pull-down assays, in which HY5 or HYH was tagged with glutathione S-transferase (GST) and RSM1 was tagged with His. As shown in Fig 9A and 9B, the *in vitro* pull-down assays illustrated a direct physical interaction between RSM1 and HY5 or HYH. *In vivo* bimolecular fluorescence complementation (BiFC) assays were performed to confirm the results of the pull-down assays. YFP^N^-RSM1 and YFP^C^-HY5 or YFP^C^-HYH were transiently co-transformed into and expressed in *Arabidopsis* mesophyll protoplasts. After overnight incubation in the dark, a YFP signal resulting from complementation between YFP^N^-RSM1 and YFP^C^-HY5 or YFP^C^-HYH was successfully detected in some of the cells by confocal microscopy. These *in vivo* data confirmed the interaction between RSM1 and HY5 or HYH (Fig 9C). Moreover, BiFC assays with particle bombardment in onion epidermal cells confirmed the interactions described above and clearly showed that the interaction of RSM1 with HY5 or HYH takes place in the nucleus (S11 Fig). In summary, different assays firmly establish that RSM1 can physically interact with HY5 or HYH.

According to previous reports, HY5 directly binds to the promoter of *ABI5* to regulate its expression [27, 30]. We have found that RSM1 binds to the *ABI5* promoter (-703 bp to -374 bp) (Fig 4), which is different from the fragment (-1754 bp to -1294 bp) bound by HY5. Given that RSM1 interacts with HY5/HYH, we speculated that RSM1 may function as a transcriptional regulator instead of as a transcription factor to regulate HY5/HYH binding to the promoter of its target gene *ABI5*, so EMSA assays were performed to test this speculation. As shown in Fig 9D, GST-HY5 indeed binds to the fragment (-1754 bp to -1294 bp) of the *ABI5* promoter; this fragment was reported previously [27]. His-RSM1 protein was unable to bind to this fragment of the *ABI5* promoter (Fig 9D). However, increasing the amounts of His-RSM1 apparently enhanced the binding of GST-HY5 to the *ABI5* promoter (Fig 9D). These findings support that RSM1 may function as a partner to enhance HY5 binding to the *ABI5* promoter, and it likely does so via direct physical interaction with HY5.

**Fig 10:**
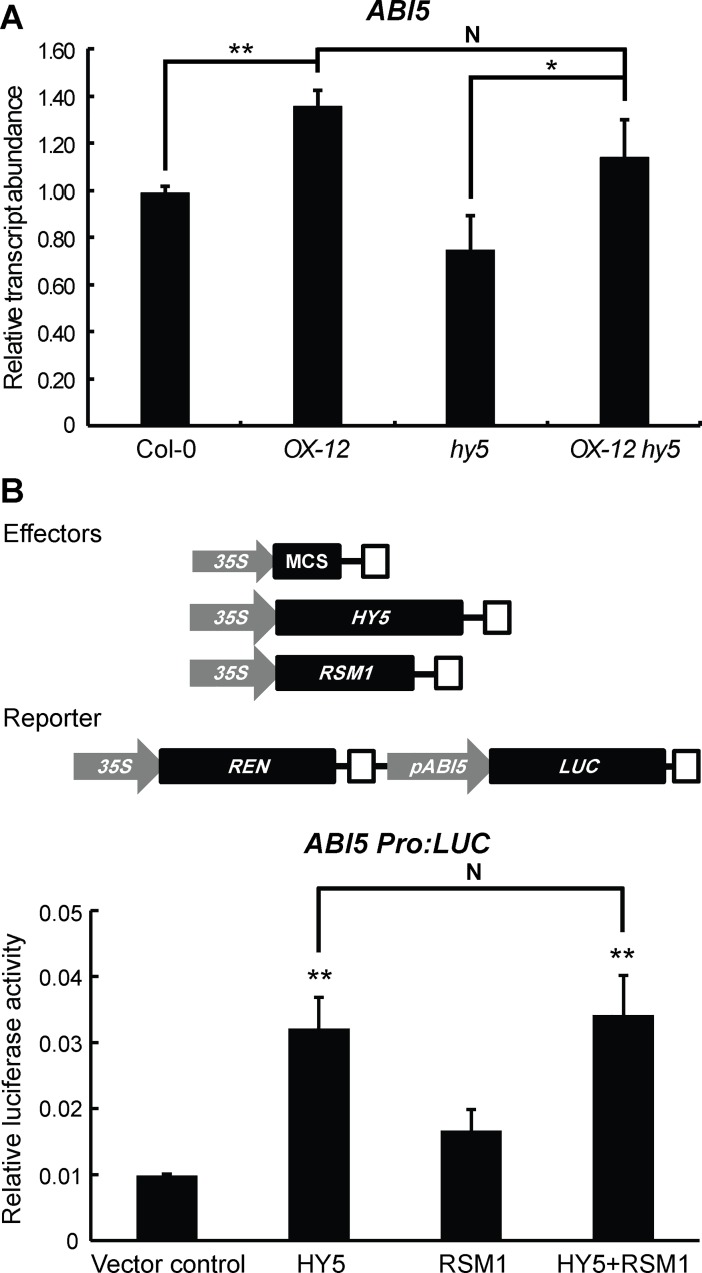
HY5 and RSM1 in regulating *ABI5* trascription. (A) HY5 and RSM1 in regulating *ABI5* trascript level in qRT-PCR assays. Col-0 WT seeds were germinated on MS medium for 3 h before harvested for RNA extraction and qRT-PCR. Information of the primers used in this assay is available in S7 Table. *ACT2* was used as a control for data normalization. Three independent replicates of measurements were performed for each genotype, and the data are shown as the mean ± standard deviation (SD) (n = 3). *p<0.05 and **p<0.01 represent significance of the differences between each pair of genotypes, N indicates that no significant difference was found between this pair of genotypes. (B) HY5 and RSM1 in regulating *ABI5* transcription in dual-luciferase transient expression assays. Upper panel: Schematic diagram of effector and reporter constructs. The rectangles filled with white stand for CaMV terminator. MCS (multiple clonal site) as the empty vector control, HY5, and RSM1 were driven by a full-length *cauliflower mosaic virus* (*CaMV*) *35S* promoter in effector constructs. The firefly luciferase in reporter construct was driven by *ABI5* promoter. Renilla luciferase (REN) driven by *35S* promoter in the reporter construct was used as an internal control. Lower panel: Dual-luciferase transient expression assays. Col-0 WT mesophyl cell protoplasts were isolated and transformed with the reporter and the effector constructs. The Y axis represents the ratios of LUC reporter activities to REN reporter activities (LUC/REN). The data are shown as the mean ± standard error (SE) (n = 4). *p<0.05 and **p<0.01 represent significance of the difference between each treatment and vector control, N indicates that no significant difference was found between this pair of treatments.

As described above, several independent assays have established that RSM1 interacts with HY5/HYH, while the EMSA assays further indicated that RSM1 enhances HY5 binding to the *ABI5* promoter. In this context, the functional implication of the interaction between RSM1 and HY5 remained unclear. To address this issue, qRT-PCR and dual-luciferase transient expression assays were conducted. As shown in Fig 10A, qRT-PCR assay revealed that overexpression of *RSM1* (in *OX-12* or *OX-12 hy5*) increased *ABI5* transcript level in comparison with that of Col-0 or *hy5*, in the presence of wild-type HY5 (in Col-0 and *OX-12*), or in the presence of non-functional mutated *hy5* (*OX-12* and *OX-12 hy5*). These results can be explained that the functional HY5 is not required for the activation of *ABI5* transcription by RSM1. In another word, RSM1 may have its own transcriptional activation activity for *ABI5* transcription. This result is also consistent with the conclusion that HY5 is upstream of RSM1 in ABA and salinity signaling based on the epistasis genetic analyses. When a comparison was made between Col-0 and *hy5*, the functional HY5 (in Col-0) was still seen to promote *ABI5* transcription. These results suggest that both HY5 and RSM1 activate *ABI5* transcription, and RSM1 may possibly not enhance the function of HY5. In dual-luciferase transient expression assay, HY5 activated the *ABI5* promoter-driven luciferase transcription, whereas RSM1 only had mild stimulation to *ABI5* promoter activity. Surprisingly, no apparent additive effect was observed when both HY5 and RSM1 constructs were supplemented (Fig 10B and 10C). This result also supports that RSM1 may not enhance the function of HY5 in activating *ABI5* promoter activity.

### RSM1 binds to its own promoter to regulate its own transcription

Transcriptional auto-regulation has been previously reported for *ABI5* [30]. Unexpectedly, yeast one-hybrid assays showed that RSM1 may bind directly to its own promoter at the site located between -523 bp to -268 bp in fragment B of the *RSM1* promoter (S12A and S12B Fig). To determine the specific binding motif, *RSM1* promoter fragment B was divided into four sub-fragments of approximately 50 bp in length for the EMSA assay. As shown in S12C Fig, RSM1 was found to bind specifically to sub-fragments *P1* and *P2*.

*GUS* reporter gene analysis was performed to evaluate the biological relevance of RSM1 to *RSM1* promoter self-binding in the context of *RSM1* expression. Five-day-old seedlings of *proRSM1*:*GUS* in the Col-0 or *rsm1* background were subjected to GUS staining. In comparison with the Col-0 background, plants of the *rsm1* background showed dramatically decreased *RSM1* promoter activity (S12D–S12F Fig). These results reveal a new regulatory mechanism for *RSM1* transcriptional regulation and its role in regulating *RSM1* responses to stresses.

## Discussion

### RSM1 plays important roles in multiple biological processes

As one of the largest plant transcription factor families, MYB transcription factors play important roles in plant growth and abiotic stress responses [33, 35]. Genetic analyses with loss-of-function mutants have shown that *RSM1* is possibly required for female gametophyte development [37]. In addition, overexpression analyses suggest that RSM1 is also possibly involved in seedling morphogenesis [34, 42, 43]. In a previous study we demonstrated that RSM1 may act as a novel repressor of the floral transition by activating *FLC* via direct binding to its promoter [40]. Additionally, several other sources of evidence hinted at possible roles of RSM1 in ABA and abiotic stress signaling. Transcriptome analyses revealed that *RSM1* is transcriptionally regulated by ABA and exposure to cold temperatures [34, 35]. *RSM1* is down-regulated in *XERICO-*overexpressing plants which have increased tolerance to drought stress [44]. In addition, *RSM1* expression is induced by cytokinins and up-regulated in *esk1*, a mutant with strong tolerance to freezing [41, 45]. The information described above implicates that RSM1 may be versatile in plant development, plant hormone signaling, and stress responses.

In the present study, the expression patterns of *RSM1* revealed by qRT-PCR and *GUS* reporter gene analyses (Figs 1 and S5) provide important information regarding the functions of RSM1 in relevant developmental processes and responses to environmental stresses. RSM1 is localized in the nuclei of stomatal guard cells and vascular tissues (S5 and S6A Figs), suggesting that it may play a role in guard cell function and vascular transport.

Genetic analyses have systematically revealed the versatility of RSM1 in many aspects. As shown in the present study, RSM1 acts as a positive regulator of ABA signaling, but it has a negative effect on tolerance to salinity and dehydration during seed germination (Fig 2), which may endow seeds with the appropriate level of sensitivity to ABA and stressful environments. In contrast, RSM1 acts as a positive regulator of salt tolerance during seedling development (Fig 3), which facilitates seedling survival under salt stress. The differential roles of RSM1 in salinity tolerance at different developmental stages may reflect different regulatory mechanisms for different biological processes. Our observations also support the previous finding [34] that RSM1 plays a positive role in seedling photomorphogenesis under red light. Furthermore, RSM1 also plays a negative role in the floral transition, as previously reported [40]. These findings show conclusively that RSM1 plays important roles in the regulation of seed germination and seedling development by ABA or abiotic stresses, in addition to several other biological processes.

Note that the loss-of-function mutant *rsm1* used in this study only exhibits clear phenotypes for particular biological processes, whereas *RSM1*-ovexpressing plants display much stronger phenotypes. This phenomenon may be ascribed to the fact that RSM1 is in low abundance *in planta*, and that the *rsm1* mutant is not a null allelic mutant. Unfortunately, the null allelic mutant for *RSM1* is arrested at the one-cell zygotic stage, as reported by Pagnussat et al. (2005) [37], thereby rendering it impractical for many functional analyses. From another point of view, the phenomenon described above may also be accounted for by the redundancy of *RSM1* and its homologous genes in terms of the biological processes assessed in this study. As described earlier”, *RSM1* has three homologous genes: *RSM2*, *RSM3* and *RSM4*. We have made *rsm1 rsm2* double and *rsm1 rsm2 rsm3* triple mutants, but these mutants do not show clear phenotypes in some assays. We are currently using the CRISPR/Cas9 approach to construct an *rsm1 rsm2 rsm3 rsm4* quadruple mutant.

### Differential interactive levels between RSM1 and HY5/HYH

We have established the involvement of RSM1 in multiple biological processes including ABA and abiotic stress responses during seed germination and post-germination seedling development. Intriguingly, HY5 and HYH are also involved in these processes [17, 25, 27, 29]. In the present study, we provide several independent lines of evidence to support our hypothesis that RSM1 is closely linked to HY5/HYH in these biological processes.

First, *RSM1* expression is regulated by HY5/HYH at the transcriptional level. As revealed by qRT-PCR analyses, *RSM1* expression is down-regulated in *hy5*, *hyh* and *hy5 hyh* mutants (Fig 6A). We provide another line of evidence from *GUS* reporter gene analyses to support the notion that *RSM1* expression is regulated by HY5 at the transcriptional level. *proRSM1*:*GUS* activity is dependent on the presence of HY5 under normal conditions (S10 Fig). Furthermore, our *in vitro* and *in vivo* assays, including yeast one-hybrid, EMSA, and ChIP-qPCR assays, revealed the biochemical mechanism underlying regulation of *RSM1* by HY5 at the transcriptional level, in which HY5 and HYH bind specifically to the C-box of the *RSM1* promoter (Fig 6B–6G). We thus conclude that HY5 and HYH may regulate *RSM1* expression at the transcriptional level, by specific binding to the *RSM1* promoter. This regulatory mechanism (e.g., promoter binding, and expression regulation) is a common means by which target genes are regulated by transcription factors [51, 52]. Indeed, many genes including *RSM1* of the HY5 regulon have been revealed by ChIP-chip assays [53]. Our data described above confirm the observation for HY5 binding to the *RSM1* promoter.

Second, RSM1 and HY5/HYH interact directly. Our *in vitro* pull-down assays (Fig 9A and 9B) showed a direct physical interaction between RSM1 and HY5 or HYH. *In vivo* bimolecular fluorescence complementation (BiFC) assays in mesophyll protoplasts confirmed the interaction between RSM1 and HY5 or HYH (Fig 9C). In addition, BiFC assay with particle bombardment in onion epidermal cells further confirmed the interactions described above, and showed that the interaction of RSM1 with HY5 or HYH takes place in the nucleus (S11 Fig). As a transcription factor, HY5 regulates *ABI5* [27], *RSM1* (the present study), and many other target genes [53]. In addition, other factors influence the manner in which HY5 regulates its target genes. As shown previously, transcriptional regulator BBX21 interferes with the binding of HY5 to the *ABI5* promoter [30]. In our assay, the binding of HY5 to the *ABI5* promoter is stimulated by RSM1 (Fig 9D). Therefore, RSM1 may possibly act as a positive transcriptional regulator in this case. With regard to influencing regulation of *ABI5* by HY5, the function of RSM1 may be fulfilled via its direct physical interaction with HY5. However, our dual-luciferase transient expression assay suggests that RSM1 does not enhance the activation of the *ABI5* promoter by HY5.

Third, *RSM1* genetically interacts with *HY5* and *HYH*. Our genetic analyses uncovered the existence of this genetic relationship. *RSM1* resides downstream of *HY5* and *HYH* during seed germination and post-germination seedling development and stress tolerance (Figs 7 and 8), no matter whether RSM1 plays a positive or negative role. The relationships between HY5/HYH and RSM1 at the transcriptional and protein levels may provide the molecular basis for their genetic relationship.

### *RSM1* is upstream of *ABI3*, *ABI4* and *ABI5* in the ABA signaling pathway

Although RSM1 may be involved in ABA responses, it is unclear whether RSM1 regulates ABA biosynthesis or signaling. No effect of RSM1 on ABA content was observed in this study, raising the possibility that RSM1 likely regulates ABA signaling rather than ABA biosynthesis. Indeed, qRT-PCR assays revealed that RSM1 regulates the transcript levels of many ABA-responsive or stress-responsive genes, such as *ABI5*, *RD29A*, *RD29B*, *AtEM1*, *AtEM6*, *RAB18*, *ABF2*, *ABF3* and *ABF4*, during seed germination (Fig 4A and S7 Fig). Considering that ABI5 is a crucial positive regulator of ABA signaling [13, 46, 47], our results confirm the role of RSM1 in ABA signaling. Given that RSM1 up-regulates *ABI5* expression (Fig 4A), our yeast one-hybrid and ChIP assays show that RSM1 can bind to the promoter of *ABI5* to induce transcriptional activation of *ABI5* (Fig 4). The question of whether RSM1 acts as a transcription factor to control *ABI5* expression is clearly prompted by our findings. Although no transactivation activity was detected for RSM1 in yeast cells (S6 Fig), our data suggest that RSM1 can function as a transcription factor to regulate *ABI5* expression, as well as act as a regulator to interact with HY5/HYH.

Our genetic analyses establish the genetic relationship between *RSM1* and *ABI5* in ABA signaling. We found that *ABI5* is downstream of *RSM1* in the ABA signaling pathways governing seed germination and seedling development (Fig 5). ABI3 is a B3-domain-containing transcription factor that physically interacts with ABI5 [54], while also functioning as an essential upstream regulator and activator of *ABI5* expression in the context of ABA signaling [49]. ABI4, an AP2/ERF transcription factor, is also important for ABA signaling during seed development and germination [14]. Like ABI3, ABI4 acts as a transcription activator to induce *ABI5* expression, by binding directly to its promoter [55]. Our genetic analyses demonstrate that similar to *ABI5*, both *ABI3* and *ABI4* are downstream of *RSM1* to mediate the functions of RSM1 in the regulation of seed germination and post-germination by ABA (S8 and S9 Figs). These results establish that RSM1 plays an important role in ABA signaling during seed germination and early seedling development.

### ABI5 is a convergence node for the functions of RSM1 and HY5/HYH in ABA signaling

In our assays, RSM1 directly binds to the *ABI5* promoter and regulates *ABI5* expression (Fig 4). HY5 binds directly to the *ABI5* promoter [27]. Interestingly, ABI5 binds to its own promoter [30]. Both HY5 and ABI5 belong to the same bZIP transcription factor family, and preferentially bind to the G-box motif. However, they bind to different G-box motifs; HY5 binds to a typical G-box motif [27] located 500 bp upstream of the ABI5-binding site within the fragment located 1127–1231 bp upstream of the start codon [30]. Several other transcription factors also bind directly to the *ABI5* promoter and regulate *ABI5*expression. FHY3/FAR1 bind to the FHY3/FAR1-binding site (FBS) [56] located 130 bp downstream of the G-box motif to which ABI5 binds [30]. ABI4 binds to a CE1-like element in the 5′-untranslated region of *ABI5* and activates its expression [55]. In addition, ABI3, a B3-domain containing transcription factor, functions as an essential regulator upstream of *ABI5* [31]. Determining whether ABI3 is also a direct regulator of *ABI5* will require further investigation. Therefore, HY5, RSM1, FHY3/FAR1, ABI4 and ABI5 bind directly to the *ABI5* promoter, but they seem to occupy different regions. Future studies should assess how these factors are coordinated and whether they could regulate the activity of one another on the *ABI5* promoter. Interestingly, BBX21 was recently reported to interfere with HY5 binding to and thereby repressing the *ABI5* promoter [30]. BBX21 is the only known negative transcriptional regulator for the *ABI5* promoter. Although our dual-luciferase transient expression assay does not show that RSM1 strengthens the activation of *ABI5* expression by HY5, our EMSA and protein-protein interaction data suggest that RSM1 may work as a partner to enhance binding of HY5 to the *ABI5* promoter, possibly via direct physical interaction with HY5. Apart from regulators upstream of *ABI5*, many ABA-responsive and stress-responsive genes are present downstream of *ABI5* and are directly regulated by ABI5. Thus, the *ABI5* promoter may represent a convergence point at which transcriptional regulators of the ABA and abiotic stress signaling pathways integrate environmental stimuli by fine-tuning the expression of *ABI5* and ABI5 target genes.

### Conclusions

**Fig 11:**
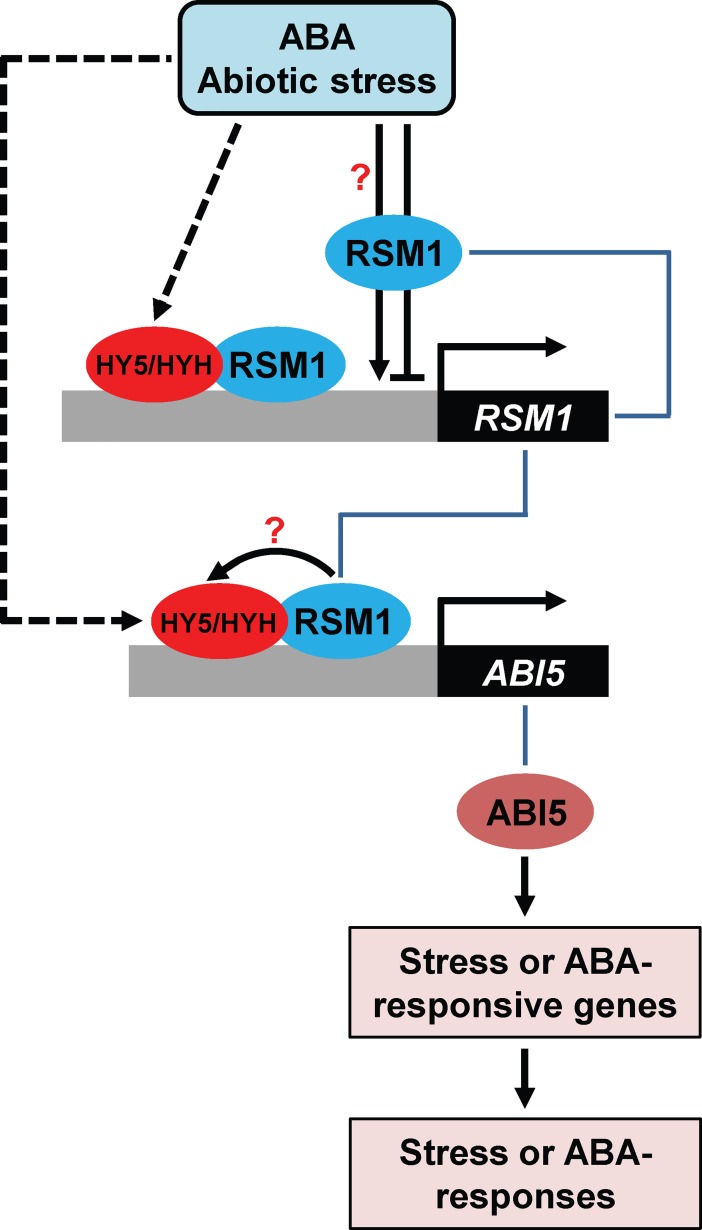
A working model of RSM1 interacting with HY5/HYH and binding with the *ABI5* promoter in ABA and abiotic stress signaling. ABA or abiotic stress such as salinity promotes *HY5/HYH* expression, while activating or suppressing *RSM1* expression depending on the duration of exposure to ABA or abiotic stress. *RSM1* expression is fine-tuned by auto-regulation by RSM1 and a regulatory loop composed of HY5/HYH. HY5/HYH and RSM1 subsequently regulate *ABI5* expression via direct binding to the *ABI5* promoter. In this process, RSM1, as a likely partner, interacts with HY5/HYH to enhance their association with the *ABI5* promoter. HY5/HYH and RSM1 independently and somehow dependently induce expression of *ABI5* and ABI5-targeted genes, thereby modulating ABA and abiotic stress responses.

When subjected to abiotic stress or ABA, plants up-regulate expression of *HY5*/*HYH*. *RSM1* may be up-regulated or down-regulated depending on the duration of exposure to ABA or abiotic stresses. There exists a regulatory mechanism in which HY5/HYH up-regulate *RSM1* expression by binding to the *RSM1* promoter. The protein RSM1 is also involved in the process of regulation of *RSM1* expression via direct binding to its own promoter. Thus, fine-tuning of *RSM1* expression may be achieved via the regulatory loop formed by both HY5/HYH and RSM1. As a direct target, *ABI5* is up-regulated by HY5 via binding to the *ABI5* promoter [27]. RSM1 may also regulate *ABI5*, and this regulatory mode may be complex. RSM1 may function as a transcription factor by directly binding to the *ABI5* promoter to accomplish up-regulation of *ABI5* expression. On the other hand, RSM1 may also function as a possible partner, interacting with HY5/HYH, although no clear evidence supports that RSM1 enhances the HY5 activation of *ABI5* expression. In summary, through the mechanisms described above, RSM1 and HY5/HYH may converge on the *ABI5* promotor, and independently or possibly dependently regulate *ABI5* expression and ABI5-targeted ABA-responsive genes, and thereby modulate ABA and abiotic stress responses (Fig 11).

## Materials and methods

### Plant materials and growth conditions

All *Arabidopsis* plants used in this study were of the Columbia-0 (Col-0) ecotype. The following mutants were used in this work: *rsm1* (*CS876657*) [34], *rsm2* (*CS371942*), *rsm2* (*Salk_069941C*), *rsm1 rsm2*, *rsm1 rsm2 rsm3*, *hy5-215* (denoted as *hy5* in the text and figures) [16], *hyh* (*CS849765*) [57], *hy5 hyh* [58], *abi5-7* [59], *abi3-8* [59], and *abi4-1* [14]. T-DNA insertion mutants *rsm1* (*CS876657*) [34], *rsm2* (*CS371942*), and *rsm2* (*Salk_069941C*) were obtained from the Arabidopsis Biological Resource Center. T-DNA insertions were confirmed by PCR on genomic DNA and sequencing of the left and right borders. *rsm1 rsm2* and *rsm1 rsm2 rsm3* mutants were generated by genetic crosses and confirmed by genomic PCR. *35S*:*TAP*-*HY5/hy5* was obtained from the Xing-Wang Deng laboratory at Peking University. Transgenic *RSM1*-overexpressing plants *OX-9* and *OX-12* [40] were also used in this study.

Seeds were surface-sterilized and stratified at 4°C for 3 days, sown onto MS media (pH 5.7–5.9) containing 1.0% sucrose and 0.8% agar, and grown at 22°C under long-day condition (16-h day/ 8-h night) for one week. Ten-day-old seedlings were then transferred to soil and grown at 22°C under long-day condition (16-h day/ 8-h night).

### Plasmid construction and plant transformation

For the GUS reporter gene essays, the ~2.4-kb long promoter of the *RSM1* gene was amplified and cloned into the pBI121 vector to generate *proRSM1*:*GUS*. The construct was then transformed into *Agrobacterium tumefaciens* GV3101 and subsequently introduced into *Arabidopsis* Col-0 by using the floral dip method [60].

### Seed germination assay

Seed germination assays were conducted as described previously [61]. Briefly, the same batches of seeds for all genotypes were surfaced sterilized, stratified at 4°C for 3 days, and plated on MS media (pH 5.7–5.9) containing 1.0% sucrose and 0.8% agar at 22°C under long-day conditions (16 h/8 h light/dark).Seed germination rates and cotyledon greening rates were typically scored and calculated every day for seven days after stratification. See the figure legends for details regarding specific days of counting and treatment with ABA, NaCl or mannitol.

### Measurements of root length

To analyze the root length of seedlings, 5-day-old seedlings grown on MS plates were transferred onto MS plates supplemented with 10 μM ABA, 20 μM ABA (Sigma), 50 mM NaCl, 100 mM NaCl, 100 mM mannitol, or 200 mM mannitol. The plates were vertically placed at 22°C in under 16-h/8-h light/dark long-day conditions for 5 days before the lengths of the primary roots were measured.

### Survival rate assay and measurement of relative electrolyte leakage under salt stress conditions

Seven-day-old seedlings (n ≥ 25) grown under normal conditions were transferred to MS media supplemented with 0 or 200 mM NaCl and grown for 3–4 days. No less than 25 seedlings were counted for the assessment of the survival rate of each genotype.

For the relative electrolyte leakage assay, salt-treated seedlings were washed with ddH_2_O and placed into 15 mL BD tubes containing 8 mL ddH_2_O. The tubes were shaken at 180–220 rpm at 22°C for 1 h, after which measurement S1 (μS/cm) was acquired using a conductivity meter (Mettler Toledo, Columbus, OH, USA). Next, the tubes were boiled for 30 min, cooled and shaken for 1–2 h at 180–220 rpm at 22°C, after which measurement S2 (μS/cm) was acquired. The reading S0 (μS/cm) was acquired from the ddH_2_O control. The relative electrolyte leakage was calculated as follows: EL (%) = (S1-S0) / (S2-S0).

### GUS staining and activity measurement

GUS staining assays were performed as described previously [62] unless stated otherwise. In brief, plant material samples were fixed with iced 90% (v/v) acetone at room temperature for 20 min and washed with iced staining buffer (50 mM sodium phosphate, 0.1% (v/v) Triton X-100, 1 mM Na_2_EDTA, 1 mM potassium ferricyanide and 1 mM potassium ferrocyanide, pH 7.0) for twice on ice. The washed materials were then incubated in GUS staining solution (staining buffer with 1 mg/mL 5-bromo-4-chloro-3-indolyl-β-glucuronic acid) at 37°C overnight. The tissue samples were cleared of chlorophyll in 7:3 (v/v) ethanol and acetic acid, after which they were twice washed with 70% (v/v) ethanol. Images were taken using a stereomicroscope (Leica, Wetzlar, Germany). The measurement of GUS activity was performed using 4-methylumbelliferyl glucuronide as described by Jefferson [62, 63].

### Subcellular localization

To visualize the subcellular localization of the GFP-RSM1 fusion protein, *35S*:*GFP-RSM1* seedlings were mounted on slides and examined under a Zeiss LSM 710 confocal microscope. GFP fluorescence was detected at 488 nm (excitation) and 490–550 nm (emission). DAPI was used to mark the nuclei.

### RNA extraction and qRT-PCR

Total RNA was extracted from one-day germinated seeds or 7-day-old *Arabidopsis* seedlings using the EasyPure Plants RNA Kit (TransGen, Beijing, China). After DNA depletion by DNase I (TransGen, Beijing, China), 1 μg total RNA was used to synthesize cDNA using ReverTra Ace qPCR RT Master Kit (Toyobo Co., Ltd., Osaka, Japan). Quantitative real-time PCR analysis was performed using SYBR Premix Ex Taq (Takara, Tokyo, Japan) in an ABI 7500 fast real-time instrument (Thermo Fisher Scientific, Waltham, MA, USA) according to the manufacturer’s instructions. The relative expression levels were normalized to internal control *ACTIN2*. The qRT-PCR assays were performed with three biological replicates, and three technical replicates were performed in each biological replicates. Information regarding the primers used in this assay is available in S7 Table.

### *In vitro* protein-DNA binding assays

Yeast one-hybrid assays were performed as described previously [64]. The *ABI5* promoter, an approximately 1200 bp sequence located upstream of the ATG start codon, was divided into six fragments, which were designated A-F. The *ABI5* promoter fragments were constructed into the pLACZ2U plasmid which has a *lacZ* reporter gene. The *RSM1* promoter (1100 bp) was divided into four fragments, which were designated A-D. The *RSM1* promoter fragments were constructed into the pLACZ2U plasmid. RSM1 CDS, HY5 CDS and HYH CDS were each constructed separately into the pB42AD plasmid. Both plasmids were introduced into yeast strain EGY48 grown on SD/gal/raf-trp-ura medium containing 5-bromo-4-chloro-3-indolyl-β-D-galactopyranoside (X-GAL) and BU salts. The yeast transformation and liquid assays were performed as described in the Yeast Protocols Handbook (Clontech, Mountain View, CA, USA). Images were taken using a digital camera (Nikon).

Electrophoretic mobility shift assays (EMSAs) were performed according to the result from the yeast one-hybrid assay. *Escherichia coli* strain *BL21* transformed with pET28a-RSM1 was induced to express His-RSM1 with 1 mM isopropyl β-D-1-thiogalactopyranoside (IPTG). The His-RSM1 fusion protein was purified with Ni-NTA beads (Qiagen, Hilden, Germany). Labeled and unlabeled probes were synthesized by Invitrogen. Protein-DNA binding assays were performed using the Scientific Light-Shift kit (Thermo Fisher Scientific). Briefly, 2 μg of His fusion proteins or GST fusion proteins were incubated together with biotin-labeled probes in a 20 μL reaction mixture containing 10 mM Tris-HCl pH 7.5, 50 mM KCl, 1 mM DTT and 50 ng/μL poly (dI∙dC). The reactions were incubated at 25°C for 20 min and separated on 4–6% native polyacrylamide gels in 0.5×TBE buffer. The gels were electroblotted to Hybond N+ nylon membranes (Millipore, Burlington, MA, USA) in 0.5×TBE for 40 min, after which the labeled probes were detected according to the instructions provided with the EMSA kit.

### ChIP assay

ChIP assays were performed as previously described [65]. Twelve-day-old Col and OX-12 seedlings grown under long-day conditions (16-h day/8-h night) were harvested and subjected to ChIP-qPCR assays using a rabbit polyclonal antibody against RSM1 to immunoprecipitate genomic DNA segments. The enrichment of DNA was analyzed by qRT-PCR. Information regarding the primers used in this assay is available in S7 Table. The enrichment value (%) was normalized to the amount of input DNA.

### Dual-luciferase transient expression assays in *Arabidopsis* mesophyll cell protoplasts

The full-length HY5 and RSM1 CDSs were cloned into the pGreen II 62-SK vector to generate the effector vectors, which were driven by the cauliflower mosaic virus 35S promoter. The 2-kb *ABI5* promoter sequence was cloned into the pGreen II 0800 vector driving firefly luciferase to generate the pro*ABI5* reporter vector. Renilla luciferase driven by a full-length cauliflower mosaic virus 35S promoter was used as an internal control. Vectors were transformed into *Arabidopsis* Col-0 WT mesophyll cell protoplasts for transient expression as described previously [66]. The transfected protoplasts were cultured at 22°C in the dark for 12 h, and firefly luciferase and *Renilla* luciferase activities were measured using the Dual-Luciferase Reporter Assay System according to the instruction manual (Promega, Madison, WI, USA).

### *In vitro* pull-down assays

Expression constructs for His-RSM1 were generated by cloning the CDS of *RSM1* into the *BamHI* and *SacI* enzyme sites of vector pET28a (Novagen, Millipore, Burlington, MA, USA). The expression constructs for GST-HY5 and GST-HYH were generated by cloning the corresponding CDSs into the *EcoRI* and *XhoI* sites of vector pGEX-4T-1 (Amersham, Little Chalfont, UK). Two micrograms of GST or GST fusion proteins were mixed with 2 μg of His-RSM1 in 500 μL GST binding buffer (50 mM Tris-HCl, pH 7.5, 100 mM NaCl, and 0.1% Nonidet P-40), after which the mixture was rotated at 4°C for 2 h. Before the proteins were mixed, the Glutathione Sepharose 4B beads were washed with GST binding buffer, which was then kept rotating at 4°C for 1 h. After four washes with GST binding buffer, the GST resin was boiled with 1×SDS loading buffer and subjected to SDS-PAGE and western blotting.

### BiFC assay

The full-length CDSs of *HYH* and *HY5* were amplified and cloned into the *SacI* and *SpeI* sites of the pSY735 (C terminus of yellow fluorescent protein [YFP^C^]) vector to generate plasmids YFP^C^-HYH and YFP^C^-HY5. Meanwhile, the full-length CDS of *RSM1* was amplified and cloned into the *SpeI* and *BamHI* sites of the pSY736 (YFP^N^) vector [67], resulting in plasmid YFP^N^-RSM1. The plasmids were extracted and concentrated to 2 mg/mL. The *in vivo* interactions were assayed by transformation using *Arabidopsis* protoplasts [66] or particle-mediated transformation using onion epidermal cells [68]. After overnight incubation in the dark, the YFP signal was detected using a Zeiss LSM 710 confocal microscope. DAPI was used to mark the nuclei.

### Western blot analysis

For immunoblotting, seedlings of *Arabidopsis* (Col-0 and other genotypes) were harvested in protein extraction buffer containing 50 mM Tris-HCl (pH 7.5), 150 mM NaCl, 10 mM MgCl_2_, 1 mM EDTA, 10 mM NaF, 2 mM Na_3_VO_4_, 25 mM β-glycerol phosphate, 10% (vol/vol) glycerol, 0.1% (vol/vol) Nonidet P-40, 1 mM PMSF and 1× cOmplete Protease Inhibitor Mixture. In brief, protein samples were separated by SDS-PAGE, after which the separate proteins were transferred to a polyvinylidene fluoride film. The film was blocked with 5% milk, incubated with the selected primary antibody overnight at 4°C, washed three times with 1× PBST (5 min each), and incubated with the selected secondary antibody for 1 h at room temperature. After three washes with 1×PBST (5 min each), the film was illuminated and photographed under a Bio-Rad illumination detection device.

### Statistical analyses

For most experiments, Student’s *t* test was employed to analyze the significance of differences between each treatment group and the appropriate control group. However, for the analyses of seed germination and cotyledon greening, one-way ANOVA and Fisher’s least significant difference (LSD) test were conducted using IBM SPSS Statistics version 20.0 (IBM Corporation, Armonk, NY, USA).

### Accession numbers

Sequence data from this article can be found in the Genome Initiative or GenBank/EMBL databases under the following accession numbers: *RSM1/MEE3* (At2g21650), *RSM2* (At4g39250), *RSM3* (At1g75250), *RSM4* (At1g19510), *HY5* (At5g11260), *HYH* (At3g17609), *ABI5* (At2g36270), *ABI3* (At3g24650), *ABI4* (At2g40220), *RD29A* (At5g52310), *RD29B* (At5g52300), *SnRK2*.*2* (At3g50500), *SnRK2*.*3* (At5g66880), *ABI1* (At4g26080), *ABI2* (At5g57050), *AtEM1* (At3g51810), *AtEM6* (At2g40170), *RAB18* (At5g66400), *ABF2* (At1g45249), *ABF3* (At4g34000), *ABF4* (At3g19290), *ACTIN2* (At3g18780) and *PP2A* (At1g69960).

## Supporting information

S1 Fig Schematic of the protein domains of RSM1 and mutation information for the *rsm1*, *rsm2* and *rsm3* mutants. (A) Diagram of *RSM* homologous genes and positions of T-DNA insertions in related mutants. Black boxes represent exons, grey lines represent introns, white boxes represent 5′ untranslated regions (UTRs) and 3 ′ UTRs, and black lines indicate parts of promoter regions. *rsm1*, *rsm1* and *rsm3* are T-DNA insertion mutants, with insertions at the 5 ′-UTR region, exon and promoter region of *At2g21650*, *At4g39250* and *At1g75250*, respectively. (B) Amplification of *RSM1*, *RSM2* and *RSM3* in genomic DNA from Col-0 and *rsm1 rsm2 rsm3* triple mutant plants. LP and RP are gene-specific T-DNA left and right border primers, respectively. (C) Schematic of the protein domains of RSM1. The black box depicts the SANT/MYB domain. (D) Phylogenetic analysis of four RSM homologs from *Arabidopsis*. The scale bar indicates branch length. (E) qRT-PCR analyses of *RSM1* transcript levels in Col-0, *rsm1*, *rsm1 rsm2* and *rsm1 rsm2 rsm3* plants. Imbibed seeds were sown on MS media and placed at 22°C under long-day condition (16 h day/8 h night) for 7 days before collected for RNA extraction and qRT-PCR analyses. *ACT2* transcript level was used as a control for data normalization. The data are shown as the mean ± SD from three independent replicate measurements (n = 3). ** indicates p<0.01 for the significance of the difference between each genotype and Col-0.
(TIF)

S2 Fig Cotyledon greening rates and relative fresh weights of *RSM1*-related genetic materials treated with ABA, NaCl or mannitol. (A) to (D) Cotyledon greening rates of *RSM1*-related materials grown on MS medium supplemented without or with 1 μM ABA, 100 mM NaCl or 200 mM mannitol. Cotyledon greening rates were determined at the indicated time from three independent replicate measurements. Approximately 100 seeds were used per genotype replicate. The data are shown as the mean ± SD (n = 3). (E) Relative fresh weights of 7-day-old seedlings of *RSM1*-related materials under treatment with ABA, NaCl or mannitol. Relative fresh weights were determined relative to the corresponding MS condition for three independent experimental replicates, and 25 seedlings were weighed per genotype replicate. The data are shown as the mean ± SD (n = 3). ** indicates p<0.01 for the significance of the difference between each genotype and Col-0.
(TIF)

S3 Fig Primary root growth of seedlings of *RSM1*-related genetic materials grown on MS medium supplemented with ABA, NaCl or mannitol. (A) Morphology of seedlings of *RSM1*-related genetic materials grown on MS medium supplemented with ABA (10 and 20 μM), NaCl (50 and 100 mM) or mannitol (100 and 200 mM). Five-day-old seedlings were transferred to different types of media and grown for 5 days before they were photographed. (B) Measurements of relative root growth. Primary root length was measured 5 days after transfer to medium supplemented with ABA, NaCl or mannitol at different concentrations. The data are normalized to the corresponding value for the MS condition, and shown as the mean ± SD (n = 10).
(TIF)

S4 Fig Na^+^ and K^+^ contents of salt-treated Col-0, *rsm1*, *rsm1 rsm2* or *OX-12* seedlings. Seven-day-old seedlings grown on MS medium were transferred to MS medium supplemented with or without 200 mM NaCl for one day before measurements of Na^+^ content (A) and K^+^ content (B), after which K^+^/Na^+^ ratios were calculated (C). The data are shown as the mean ± SD from three independent replicate measurements (n = 3). dw, dry weight.
(TIF)

S5 Fig Expression pattern and localization of RSM1. (A-L) Histochemical localization of *proRSM1*:*GUS* activity in dry seeds (A), germinating seeds (B and C), 3-day-old seedlings (D), 4-day-old seedlings (E), 5-day-old seedlings (F), 7-day-old seedlings (G), 14-day-old seedlings (H), rosette leaves (I), inflorescences (J), flowers (K) and siliques (L). The images were taken under a stereomicroscope. (M) RSM1 localization in cotyledons, hypocotyls and roots of 5-day-old *35S*:*GFP-RSM1* seedlings. The images were taken under a confocal microscope. DAPI was used to label the nuclei. The scale bar indicates 0.5 mm (A-C, K), 1 mm (J, L), 2 mm (D), 4 mm (E-H), 1 cm (I), or 20 μm (M).
(TIF)

S6 Fig RSM1 localization in the leaves of plants and RSM1 transactivation assay in yeast. (A) RSM1 localization in the leaves of plants. RSM1 localization assays were performed with 1-month-old *35S*:*GFP-RSM1* plants under a confocal microscope. The sale bar indicates 20 μm. (B) Assay of RSM1 transactivation activity in yeast cells. pLexA-PIF3 was used as a positive control and pLexA was used as a negative control. The RSM1 CDS and PIF3 CDS were constructed separately into the pLexA plasmid. The plasmids were introduced into yeast strain EGY48[p8op-lacZ]. RSM1 transactivation activity was assessed on SD/gal/raf-trp-ura medium containing 5-bromo-4-chloro-3-indolyl-β-D-galactopyranoside (X-GAL) and BU salts. Images were taken using a digital camera (Nikon). (C) RSM1 expression in yeast cells. Yeast cells were cultured for 16–18 h and harvested for isolation of total proteins for immunoblot analysis. Anti-RSM1 was used to immunoblot RSM1.
(TIF)

S7 Fig Expression of ABA responsive genes in *RSM1*-related genetic materials treated with or without ABA during seed germination. Total RNA was isolated from 1-day-old germinating seeds grown on MS medium supplemented with or without 0.2 μM ABA before qRT-PCR analyses. The data are normalized to the reference gene *ACT2*. The data are shown as the mean ± SD from three replicate measurements (n = 3). Different letters on the top of each column represent significant differences (p<0.01) between any pair of data according to Student’s *t* test.
(TIF)

S8 Fig Genetic relationship between *RSM1* and *ABI3*. (A) Morphology of 7-day-old seedlings (Col-0, *abi3-8*, *OX-12* and *OX-12 abi3-8*) grown on MS medium with or without 1 or 5 μM ABA. The scale bar indicates 0.5 cm. (B) to (D) Germination rates of Col-0, *abi3-8*, *OX-12* and *OX-12 abi3-8* seeds grown on MS medium with or without different concentrations of ABA (0, 1 and 5 μM). Germination rates were determined at the indicated time. (E) Relative fresh weights of 7-day-old seedlings as illustrated in (A). Relative fresh weights were determined at the indicated time. The data are shown as the mean ± SD (n = 3) from three independent replicate experiments. The fresh weights of 25 seedlings were weighed for genotype replicate. ** indicates p<0.01 for the significance of the difference between each genotype and Col-0. (F) to (H) Cotyledon greening rates of Col-0, *abi3-8*, *OX-12* and *OX-12 abi3-8* seedlings. Cotyledon greening rates were scored and calculated at the indicated time. The data are shown as the mean ± SD from three independent replicate experiments (n = 3). Approximately 100 seeds were used per genotype replicate.
(TIF)

S9 Fig Genetic relationship between *RSM1* and *ABI4* in ABA regulation of seed germination and cotyledon greening. (A) Morphology of 7-d-old seedlings (Col-0, *abi4-1*, *OX-12* and *OX-12 abi4-1*) grown on MS medium with or without 1, 3 or 5 μM ABA. The scale bar indicates 1 cm. (B) to (E) Germination rates of Col-0, *abi4-1*, *OX-12* and *OX-12 abi4-1* seedlings grown on MS medium with or without different concentrations of ABA (0, 1, 3 and 5 μM). (F), (G) Cotyledon greening rates of Col-0, *abi4-1*, *OX-12* and *OX-12 abi4-1* seedlings grown on MS medium with or without different concentrations of ABA (0, 1 μM). Germination rates and cotyledon greening rates were scored and calculated at the indicated time. The data are shown as the mean ± SD from three independent replicate experiments (n = 3). Approximately 100 seeds were used per genotype replicate.
(TIF)

S10 Fig Reduced *proRSM1*:*GUS* activity in the *hy5* mutant. (A), (B) Histochemical analysis of *proRSM1*:*GUS* in Col-0 (A) and *hy5* plants (B). Seedlings grown on MS medium for 3 days in constant white light before GUS staining and photographing. The scale bar indicates 1 mm. (C) GUS activity measurement of 3-day-old Col-0 and *hy5* seedlings. The data are shown as the mean ± SD (n = 3). ** indicates p<0.01 for the significance of the difference between *hy5* and Col-0.
(TIF)

S11 Fig RSM1 interacts with HY5/HYH in BiFC assays with onion epidermal cells. YFP^N^-RSM1 and YFP^C^-HY5/HYH were transiently co-transformed into onion epidermal cells by particle bombardment. After overnight incubation in the dark, the YFP signal was detected by confocal microscopy. DAPI was used to label nuclei. Pairs of empty vectors (YFP^N^ and YFP^C^) or pairs of either YFP^N^ or YFP^C^ and another YFP^C^ or YFP^N^-fused vector were used as negative controls. The scale bar stands for 10 μm.
(TIF)

S12 Fig RSM1 binds its own promoter to regulate *RSM1* expression. (A) Diagram of the *RSM1* promoter fragments used to drive *LacZ* reporter gene expression in yeast one-hybrid assays (B). (B) Yeast one-hybrid assays to RSM1 binding to the *RSM1* promoter. *EGY48* cells were co-transformed with pB42AD-RSM1 or pB42AD and the *pLacZ2U-RSM1* promoter. pB42AD was used as a control. (C) EMSAs to assess RSM1 binding to the *RSM1* promoter. (D), (E) Histochemical analysis of *proRSM1*:*GUS* in Col-0 (D) and *rsm1* (E). Seedlings were grown on MS medium for 5 days in constant white light before GUS staining and photographing. Scale bar indicates 1 mm. (F) *proRSM1*:*GUS* activity in Col-0 and *rsm1* seedlings. The data are shown as the mean ± SD (n = 3). ** indicates p<0.01 for the significance of the difference between *rsm1* and Col-0.
(TIF)

S1 Table Statistical analysis for the data shown in Fig 2B–2G. (DOCX)

S2 Table Statistical analysis for the data shown in S2A–S2D Fig. (DOCX)

S3 Table Statistical analysis for the data shown in Fig 5B–5G. (DOCX)

S4 Table Statistical analysis for the data shown in S8B–S8D and S8F–S8H Fig. (DOCX)

S5 Table Statistical analysis for the data shown in S9B–S9G Fig. (DOCX)

S6 Table Statistical analysis for data shown in Fig 7B–7G. (DOCX)

S7 Table List of primers used in this study. (DOCX)
