# Supplementary figures and images for: RSM1, an Arabidopsis MYB protein, interacts with HY5/HYH to modulate seed germination and seedling development in response to abscisic acid and salinity
Source: PLoS Genet. 2018 Dec 19;14(12):e1007839. doi: 10.1371/journal.pgen.1007839 (PMC6317822; doi:10.1371/journal.pgen.1007839)

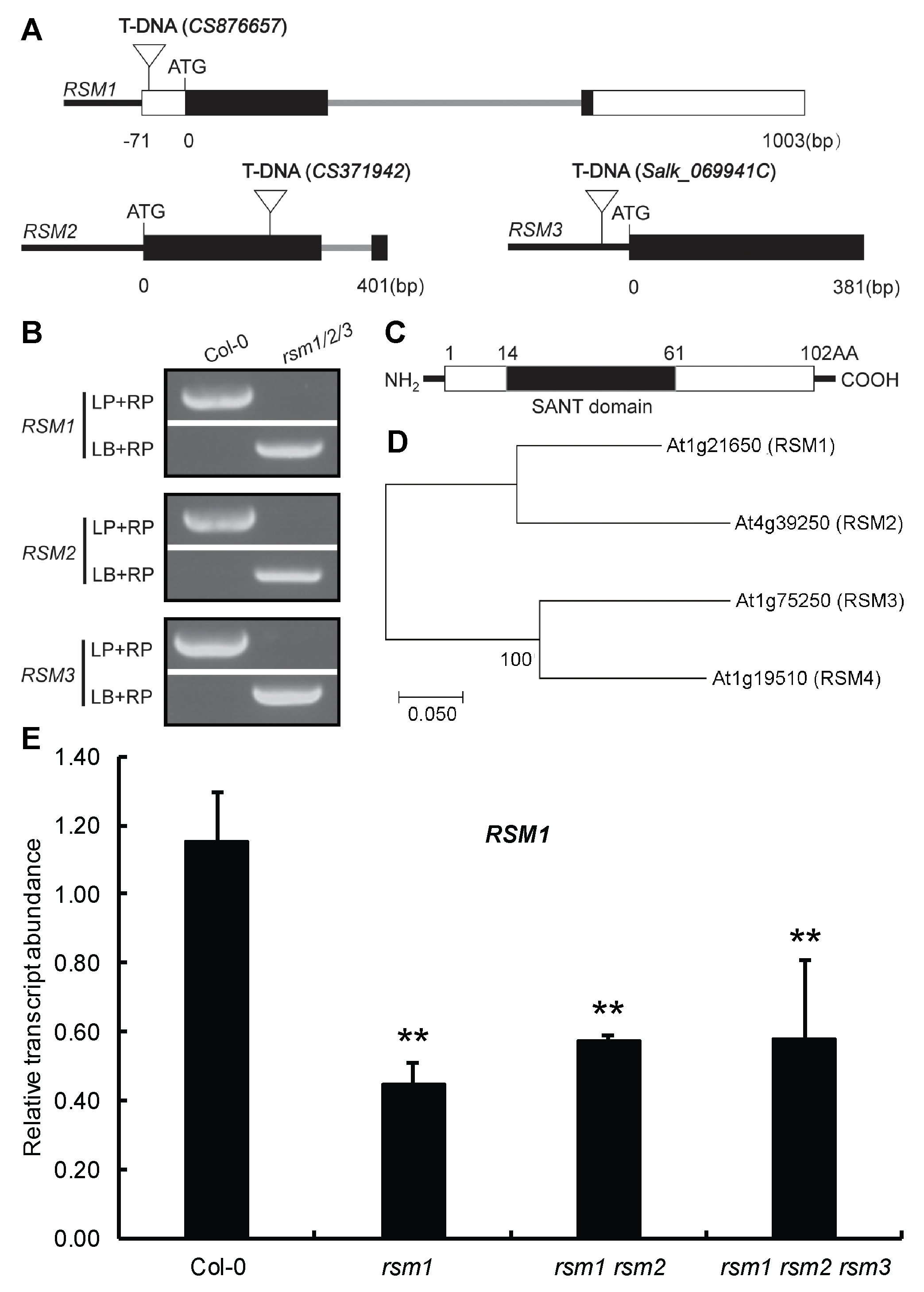

Supplement: S1 Fig — (A) Diagram of RSM homologous genes and positions of T-DNA insertions in related mutants. Black boxes represent exons, grey lines represent introns, white boxes represent 5′ untranslated regions (UTRs) and 3 ′ UTRs, and black lines indicate parts of promoter regions. rsm1, rsm1 and rsm3 are T-DNA insertion mutants, with insertions at the 5 ′-UTR region, exon and promoter region of At2g21650, At4g39250 and At1g75250, respectively. (B) Amplification of RSM1, RSM2 and RSM3 in genomic DNA from Col-0 and rsm1 rsm2 rsm3 triple mutant plants. LP and RP are gene-specific T-DNA left and right border primers, respectively. (C) Schematic of the protein domains of RSM1. The black box depicts the SANT/MYB domain. (D) Phylogenetic analysis of four RSM homologs from Arabidopsis. The scale bar indicates branch length. (E) qRT-PCR analyses of RSM1 transcript levels in Col-0, rsm1, rsm1 rsm2 and rsm1 rsm2 rsm3 plants. Imbibed seeds were sown on MS media and placed at 22°C under long-day condition (16 h day/8 h night) for 7 days before collected for RNA extraction and qRT-PCR analyses. ACT2 transcript level was used as a control for data normalization. The data are shown as the mean ± SD from three independent replicate measurements (n = 3). ** indicates p<0.01 for the significance of the difference between each genotype and Col-0. (TIF) [file pgen.1007839.s001.tif]

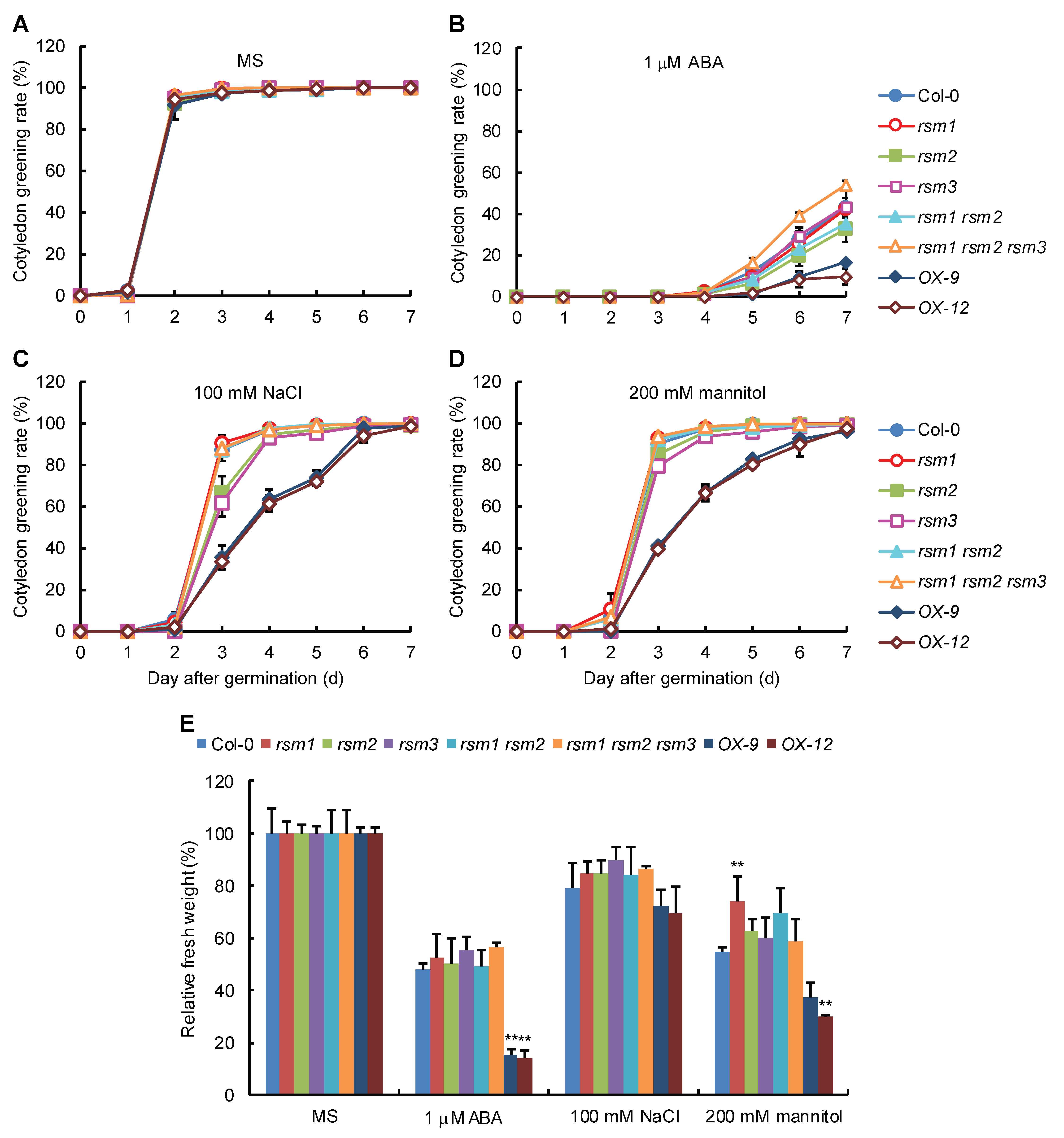

Supplement: S2 Fig — (A) to (D) Cotyledon greening rates of RSM1-related materials grown on MS medium supplemented without or with 1 μM ABA, 100 mM NaCl or 200 mM mannitol. Cotyledon greening rates were determined at the indicated time from three independent replicate measurements. Approximately 100 seeds were used per genotype replicate. The data are shown as the mean ± SD (n = 3). (E) Relative fresh weights of 7-day-old seedlings of RSM1-related materials under treatment with ABA, NaCl or mannitol. Relative fresh weights were determined relative to the corresponding MS condition for three independent experimental replicates, and 25 seedlings were weighed per genotype replicate. The data are shown as the mean ± SD (n = 3). ** indicates p<0.01 for the significance of the difference between each genotype and Col-0. (TIF) [file pgen.1007839.s002.tif]

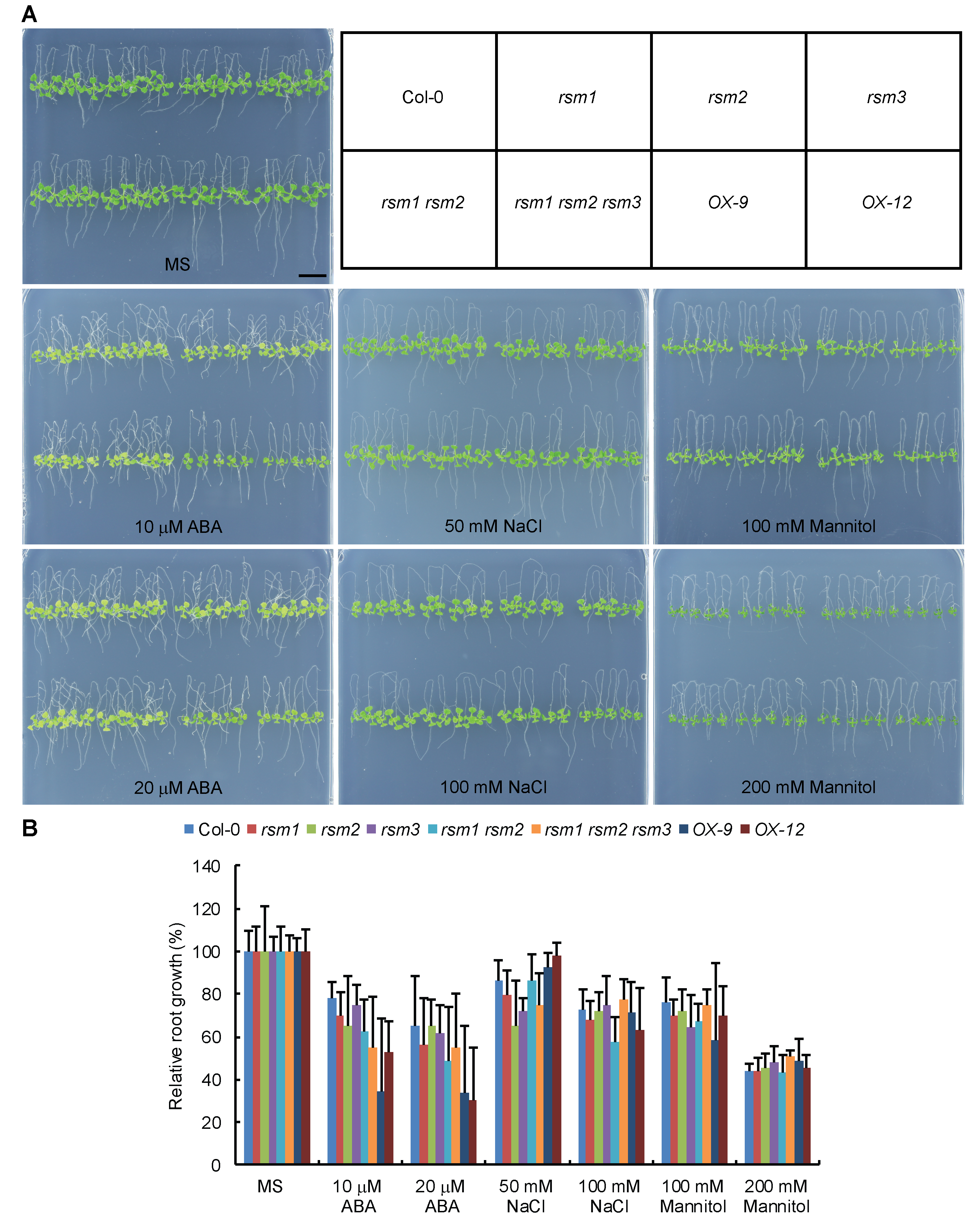

Supplement: S3 Fig — (A) Morphology of seedlings of RSM1-related genetic materials grown on MS medium supplemented with ABA (10 and 20 μM), NaCl (50 and 100 mM) or mannitol (100 and 200 mM). Five-day-old seedlings were transferred to different types of media and grown for 5 days before they were photographed. (B) Measurements of relative root growth. Primary root length was measured 5 days after transfer to medium supplemented with ABA, NaCl or mannitol at different concentrations. The data are normalized to the corresponding value for the MS condition, and shown as the mean ± SD (n = 10). (TIF) [file pgen.1007839.s003.tif]

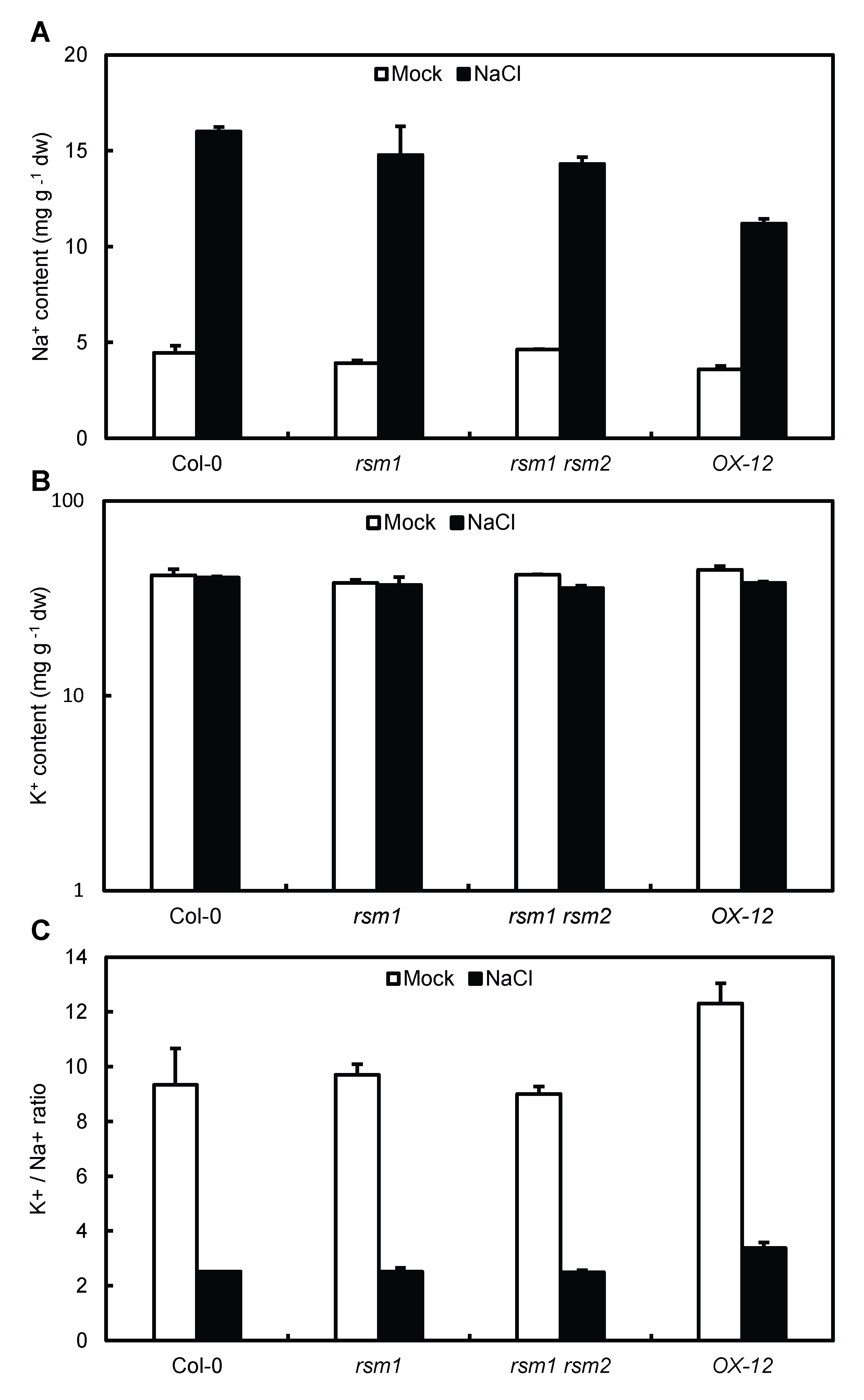

Supplement: S4 Fig — Seven-day-old seedlings grown on MS medium were transferred to MS medium supplemented with or without 200 mM NaCl for one day before measurements of Na+ content (A) and K+ content (B), after which K+/Na+ ratios were calculated (C). The data are shown as the mean ± SD from three independent replicate measurements (n = 3). dw, dry weight. (TIF) [file pgen.1007839.s004.tif]

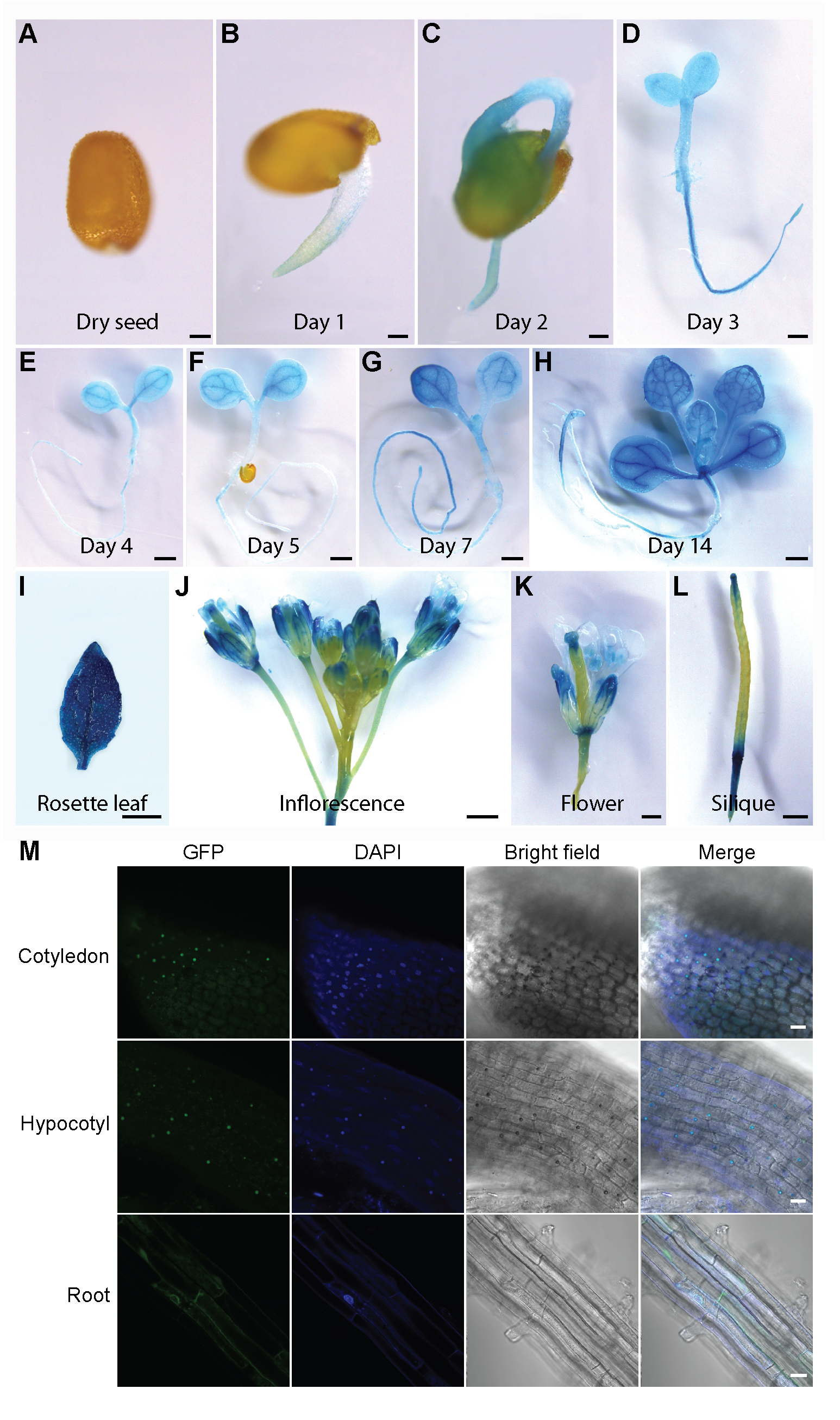

Supplement: S5 Fig — (A-L) Histochemical localization of proRSM1:GUS activity in dry seeds (A), germinating seeds (B and C), 3-day-old seedlings (D), 4-day-old seedlings (E), 5-day-old seedlings (F), 7-day-old seedlings (G), 14-day-old seedlings (H), rosette leaves (I), inflorescences (J), flowers (K) and siliques (L). The images were taken under a stereomicroscope. (M) RSM1 localization in cotyledons, hypocotyls and roots of 5-day-old 35S:GFP-RSM1 seedlings. The images were taken under a confocal microscope. DAPI was used to label the nuclei. The scale bar indicates 0.5 mm (A-C, K), 1 mm (J, L), 2 mm (D), 4 mm (E-H), 1 cm (I), or 20 μm (M). (TIF) [file pgen.1007839.s005.tif]

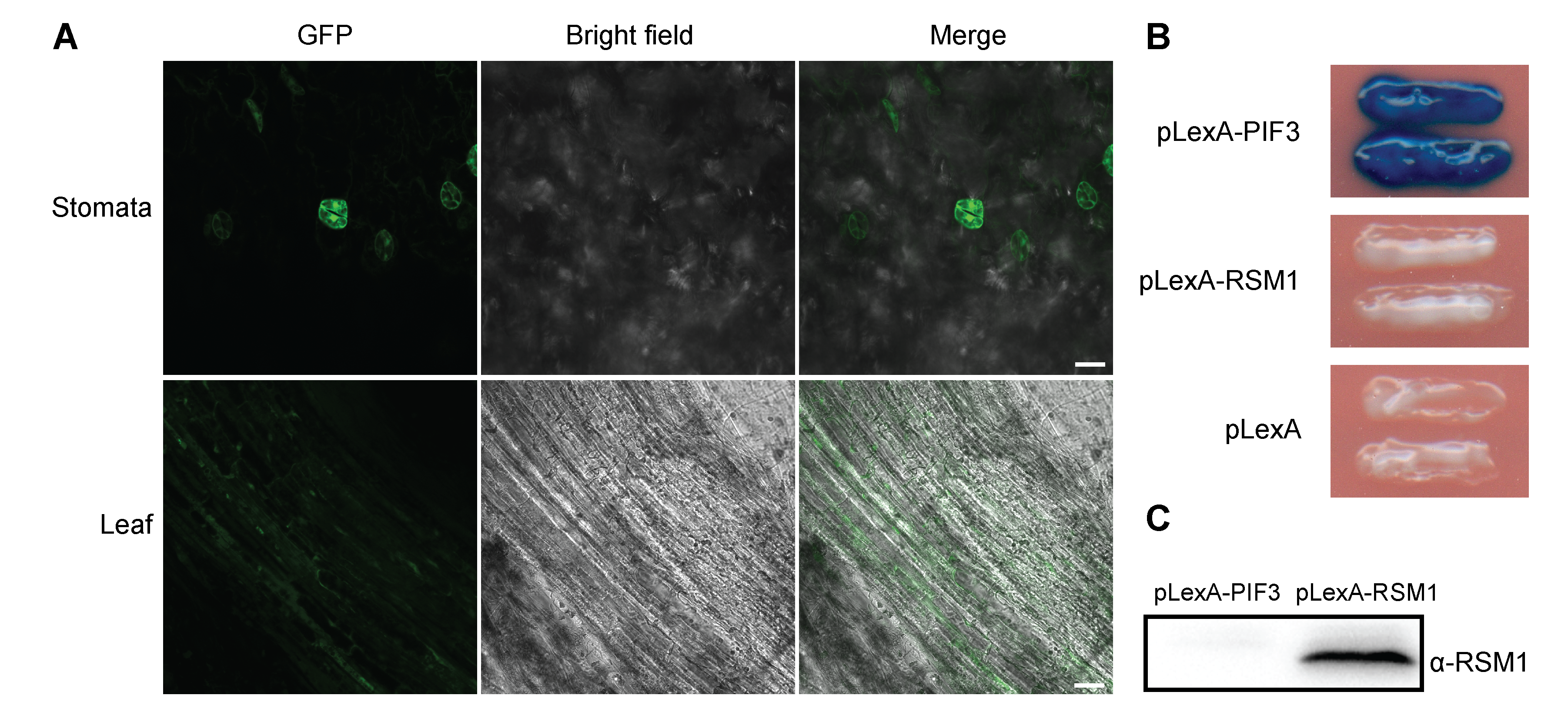

Supplement: S6 Fig — (A) RSM1 localization in the leaves of plants. RSM1 localization assays were performed with 1-month-old 35S:GFP-RSM1 plants under a confocal microscope. The sale bar indicates 20 μm. (B) Assay of RSM1 transactivation activity in yeast cells. pLexA-PIF3 was used as a positive control and pLexA was used as a negative control. The RSM1 CDS and PIF3 CDS were constructed separately into the pLexA plasmid. The plasmids were introduced into yeast strain EGY48[p8op-lacZ]. RSM1 transactivation activity was assessed on SD/gal/raf-trp-ura medium containing 5-bromo-4-chloro-3-indolyl-β-D-galactopyranoside (X-GAL) and BU salts. Images were taken using a digital camera (Nikon). (C) RSM1 expression in yeast cells. Yeast cells were cultured for 16–18 h and harvested for isolation of total proteins for immunoblot analysis. Anti-RSM1 was used to immunoblot RSM1. (TIF) [file pgen.1007839.s006.tif]

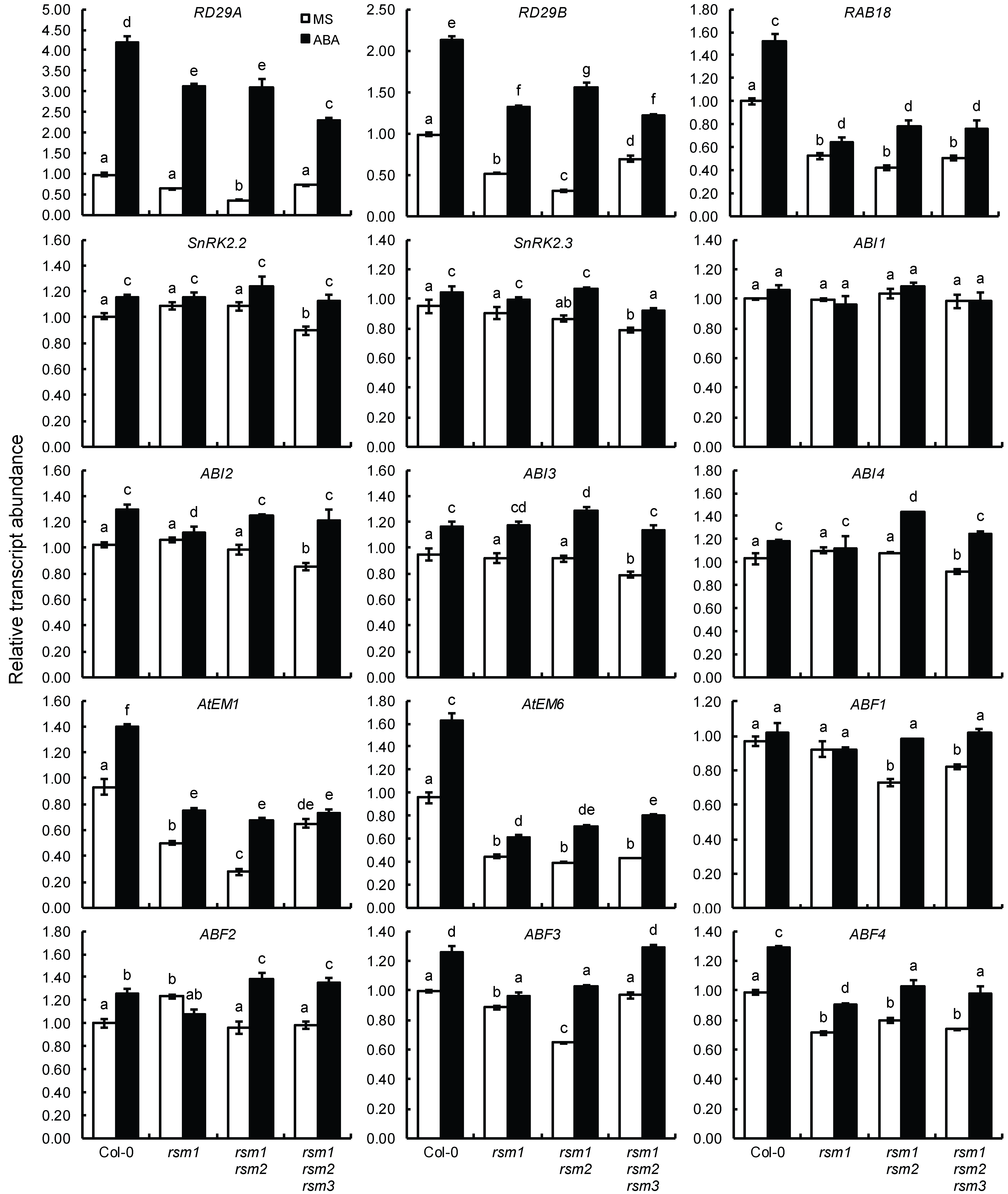

Supplement: S7 Fig — Total RNA was isolated from 1-day-old germinating seeds grown on MS medium supplemented with or without 0.2 μM ABA before qRT-PCR analyses. The data are normalized to the reference gene ACT2. The data are shown as the mean ± SD from three replicate measurements (n = 3). Different letters on the top of each column represent significant differences (p<0.01) between any pair of data according to Student’s t test. (TIF) [file pgen.1007839.s007.tif]

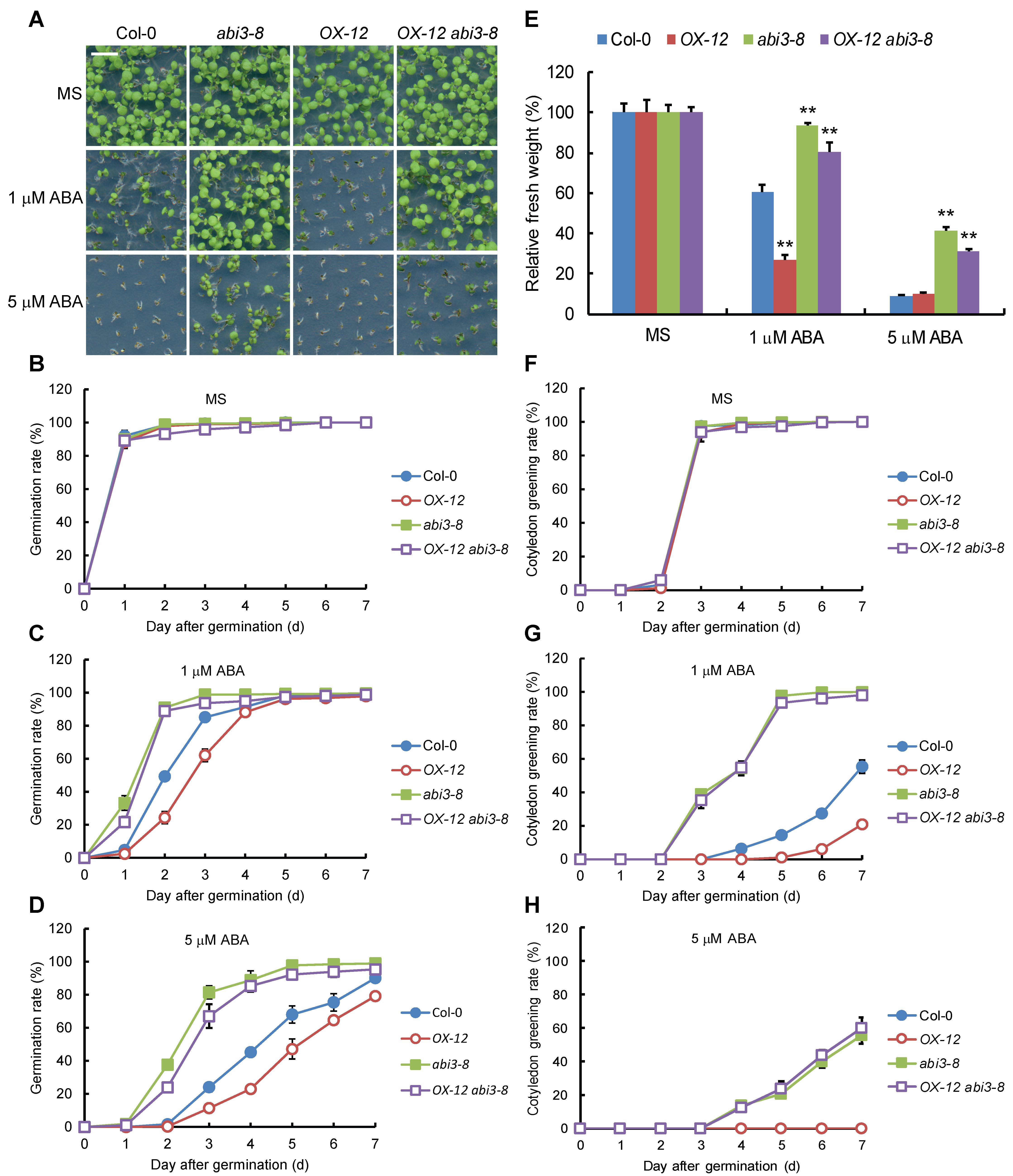

Supplement: S8 Fig — (A) Morphology of 7-day-old seedlings (Col-0, abi3-8, OX-12 and OX-12 abi3-8) grown on MS medium with or without 1 or 5 μM ABA. The scale bar indicates 0.5 cm. (B) to (D) Germination rates of Col-0, abi3-8, OX-12 and OX-12 abi3-8 seeds grown on MS medium with or without different concentrations of ABA (0, 1 and 5 μM). Germination rates were determined at the indicated time. (E) Relative fresh weights of 7-day-old seedlings as illustrated in (A). Relative fresh weights were determined at the indicated time. The data are shown as the mean ± SD (n = 3) from three independent replicate experiments. The fresh weights of 25 seedlings were weighed for genotype replicate. ** indicates p<0.01 for the significance of the difference between each genotype and Col-0. (F) to (H) Cotyledon greening rates of Col-0, abi3-8, OX-12 and OX-12 abi3-8 seedlings. Cotyledon greening rates were scored and calculated at the indicated time. The data are shown as the mean ± SD from three independent replicate experiments (n = 3). Approximately 100 seeds were used per genotype replicate. (TIF) [file pgen.1007839.s008.tif]

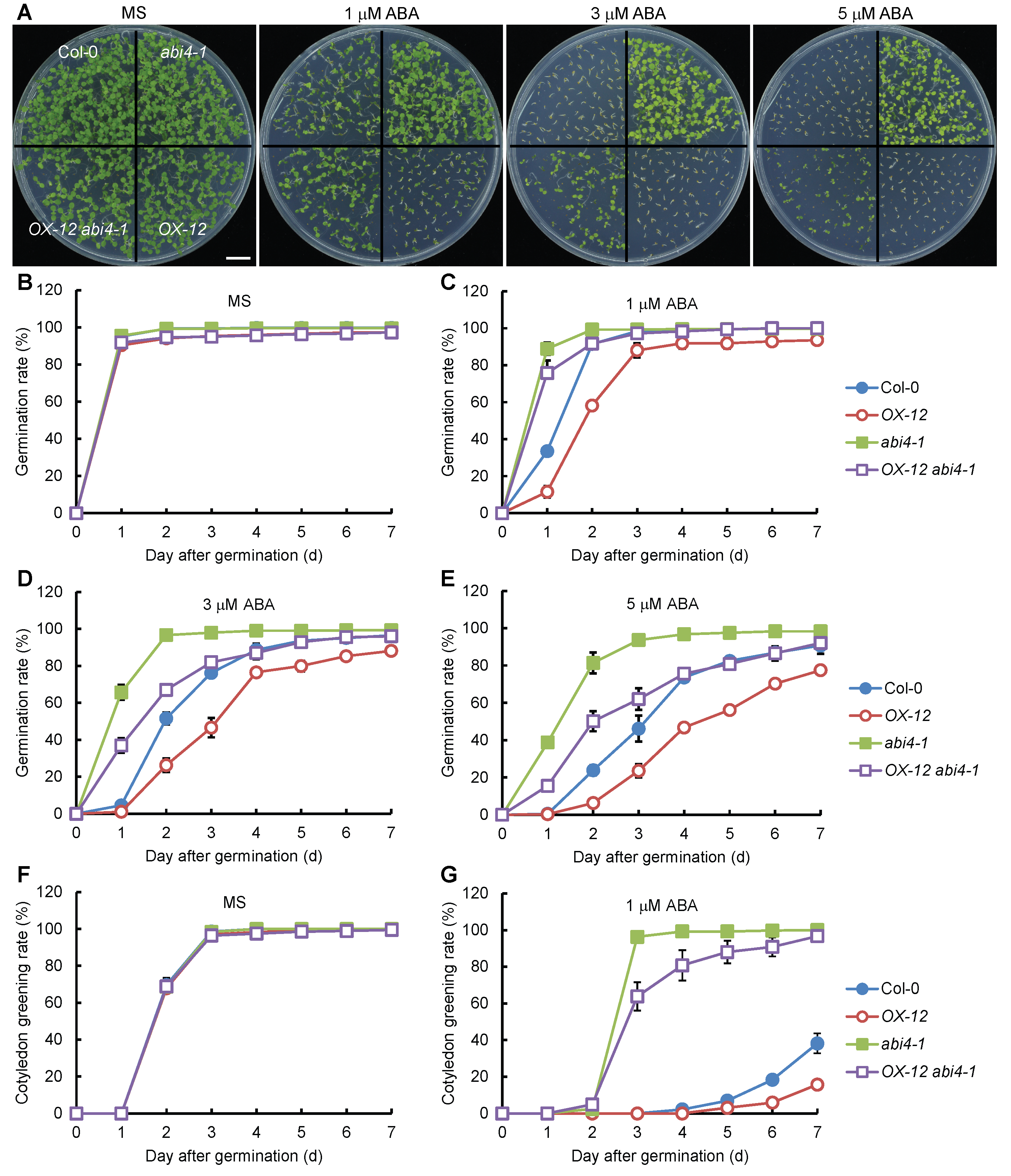

Supplement: S9 Fig — (A) Morphology of 7-d-old seedlings (Col-0, abi4-1, OX-12 and OX-12 abi4-1) grown on MS medium with or without 1, 3 or 5 μM ABA. The scale bar indicates 1 cm. (B) to (E) Germination rates of Col-0, abi4-1, OX-12 and OX-12 abi4-1 seedlings grown on MS medium with or without different concentrations of ABA (0, 1, 3 and 5 μM). (F), (G) Cotyledon greening rates of Col-0, abi4-1, OX-12 and OX-12 abi4-1 seedlings grown on MS medium with or without different concentrations of ABA (0, 1 μM). Germination rates and cotyledon greening rates were scored and calculated at the indicated time. The data are shown as the mean ± SD from three independent replicate experiments (n = 3). Approximately 100 seeds were used per genotype replicate. (TIF) [file pgen.1007839.s009.tif]

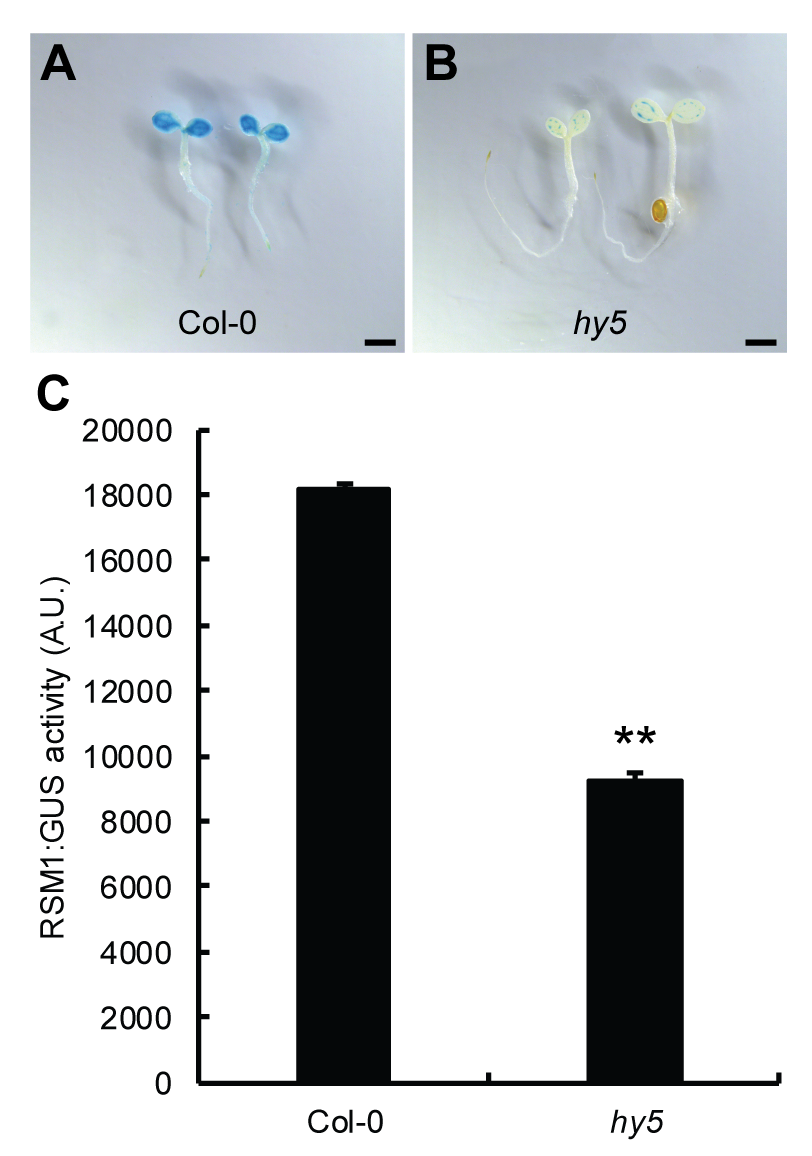

Supplement: S10 Fig — (A), (B) Histochemical analysis of proRSM1:GUS in Col-0 (A) and hy5 plants (B). Seedlings grown on MS medium for 3 days in constant white light before GUS staining and photographing. The scale bar indicates 1 mm. (C) GUS activity measurement of 3-day-old Col-0 and hy5 seedlings. The data are shown as the mean ± SD (n = 3). ** indicates p<0.01 for the significance of the difference between hy5 and Col-0. (TIF) [file pgen.1007839.s010.tif]

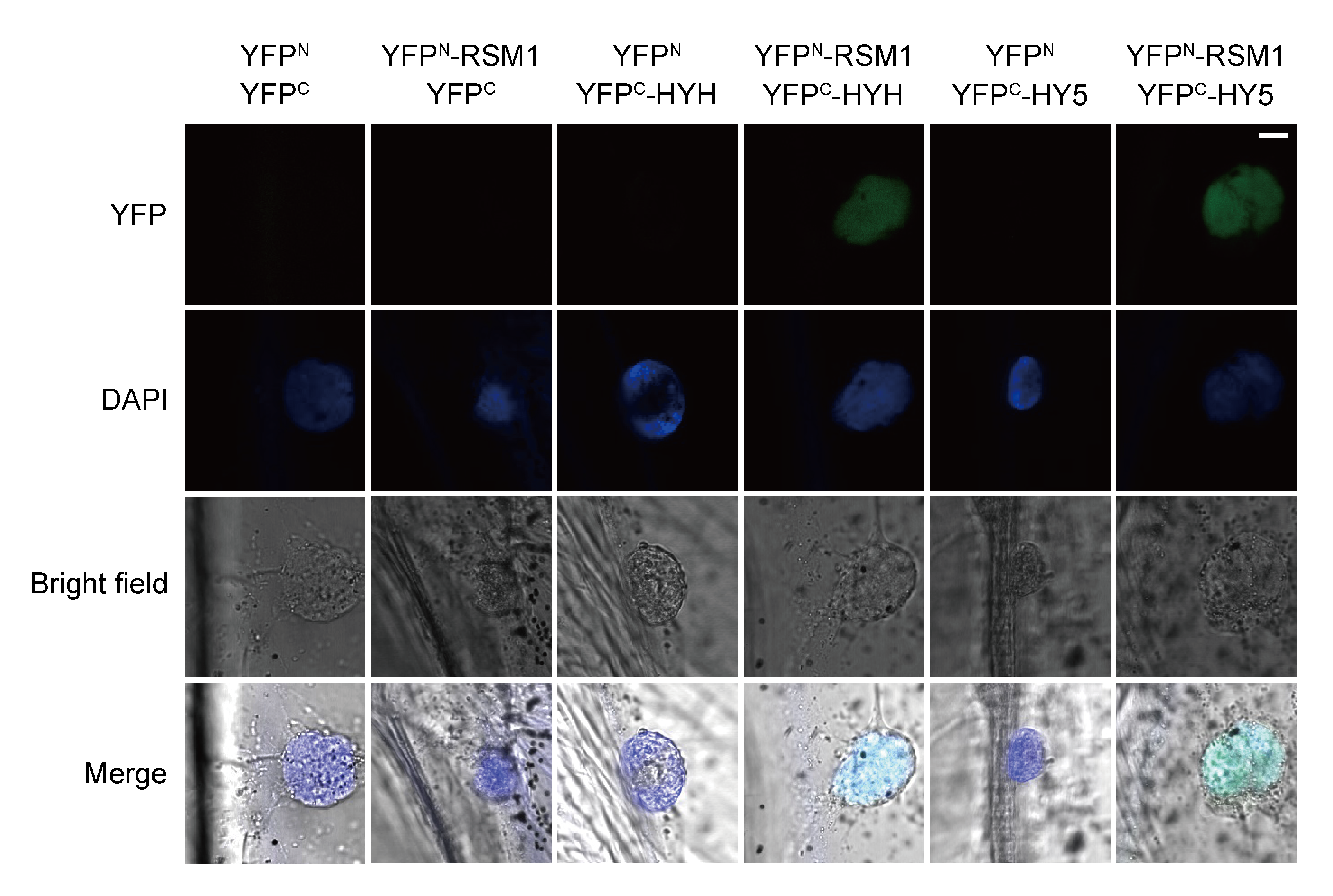

Supplement: S11 Fig — YFPN-RSM1 and YFPC-HY5/HYH were transiently co-transformed into onion epidermal cells by particle bombardment. After overnight incubation in the dark, the YFP signal was detected by confocal microscopy. DAPI was used to label nuclei. Pairs of empty vectors (YFPN and YFPC) or pairs of either YFPN or YFPC and another YFPC or YFPN-fused vector were used as negative controls. The scale bar stands for 10 μm. (TIF) [file pgen.1007839.s011.tif]

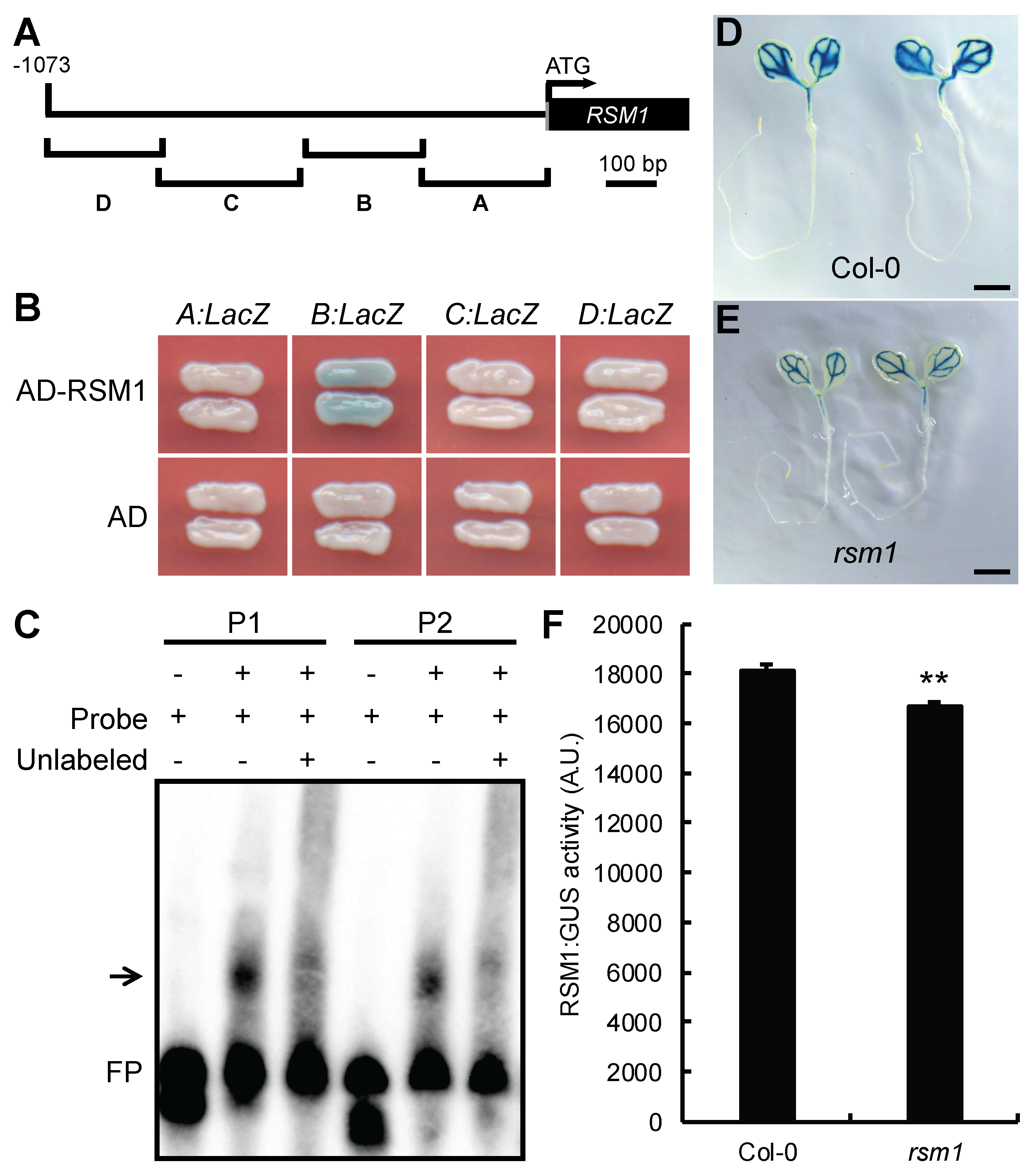

Supplement: S12 Fig — (A) Diagram of the RSM1 promoter fragments used to drive LacZ reporter gene expression in yeast one-hybrid assays (B). (B) Yeast one-hybrid assays to RSM1 binding to the RSM1 promoter. EGY48 cells were co-transformed with pB42AD-RSM1 or pB42AD and the pLacZ2U-RSM1 promoter. pB42AD was used as a control. (C) EMSAs to assess RSM1 binding to the RSM1 promoter. (D), (E) Histochemical analysis of proRSM1:GUS in Col-0 (D) and rsm1 (E). Seedlings were grown on MS medium for 5 days in constant white light before GUS staining and photographing. Scale bar indicates 1 mm. (F) proRSM1:GUS activity in Col-0 and rsm1 seedlings. The data are shown as the mean ± SD (n = 3). ** indicates p<0.01 for the significance of the difference between rsm1 and Col-0. (TIF) [file pgen.1007839.s012.tif]
