# Supplementary material for: RSM1, an Arabidopsis MYB protein, interacts with HY5/HYH to modulate seed germination and seedling development in response to abscisic acid and salinity
Source: PLoS Genet. 2018 Dec 19;14(12):e1007839. doi: 10.1371/journal.pgen.1007839 (PMC6317822; doi:10.1371/journal.pgen.1007839)
Supplement: S1 Table — (DOCX) [file pgen.1007839.s013.docx]

**S1 Table. *P*-values of comparisons between each genotype and Col-0 in terms of seed germination rates in Fig 2B-G. The data were tested by one-way ANOVA, followed by LSD test using IBM SPSS Statistics Version 20.0.**

| Fig 2B: MS | | | | | | | |
| --- | --- | --- | --- | --- | --- | --- | --- |
|  | Day 1 | Day 2 | Day 3 | Day 4 | Day 5 | Day 6 | Day 7 |
| *rsm1* | .483 | .513 | .647 | .539 | .583 | .344 | .344 |
| *rsm2* | .196 | .864 | .545 | .064 | .210 | .133 | .133 |
| *rsm3* | .238 | .421 | .796 | 1.000 | 1.000 | 1.000 | 1.000 |
| *rsm1 rsm2* | .419 | .970 | .470 | .018 | .084 | 1.000 | 1.000 |
| *rsm1 rsm2 rsm3* | .692 | .182 | .633 | 1.000 | 1.000 | 1.000 | 1.000 |
| *OX-9* | .044 | .097 | .242 | .014 | .167 | .471 | .471 |
| *OX-12* | .079 | .029 | .142 | .019 | .237 | 1.000 | 1.000 |
|  |  |  |  |  |  |  |  |
| Fig 2C: 1 μM ABA | | | | | | | |
|  | Day 1 | Day 2 | Day 3 | Day 4 | Day 5 | Day 6 | Day 7 |
| *rsm1* | .001 | .007 | .395 | .354 | .562 | .692 | .694 |
| *rsm2* | .952 | .423 | .372 | .649 | .791 | .805 | .907 |
| *rsm3* | .048 | .305 | .488 | .691 | .565 | .610 | .613 |
| *rsm1 rsm2* | .012 | .137 | .556 | .721 | .736 | .815 | .886 |
| *rsm1 rsm2 rsm3* | .000 | .024 | .681 | .568 | .861 | .809 | .811 |
| *OX-9* | .640 | .023 | .001 | .000 | .002 | .021 | .028 |
| *OX-12* | .058 | .000 | .000 | .000 | .000 | .000 | .001 |
|  |  |  |  |  |  |  |  |
| Fig 2D: 5 μM ABA | | | | | | | |
|  | Day 1 | Day 2 | Day 3 | Day 4 | Day 5 | Day 6 | Day 7 |
| *rsm1* | .376 | .080 | .031 | .008 | .002 | .016 | .034 |
| *rsm2* | .422 | .990 | .505 | .485 | .251 | .547 | .801 |
| *rsm3* | .680 | .378 | .080 | .219 | .223 | .511 | .925 |
| *rsm1 rsm2* | .422 | .746 | .293 | .523 | .529 | .650 | .623 |
| *rsm1 rsm2 rsm3* | .917 | .376 | .053 | .080 | .063 | .974 | .111 |
| *OX-9* | .952 | .679 | .228 | .146 | .015 | .000 | .000 |
| *OX-12* | .628 | .215 | .018 | .002 | .000 | .000 | .000 |
|  |  |  |  |  |  |  |  |
| Fig 2E: 100 mM NaCl | | | | | | | |
|  | Day 1 | Day 2 | Day 3 | Day 4 | Day 5 | Day 6 | Day 7 |
| *rsm1* | .315 | .376 | .027 | .560 | .835 | .698 | .376 |
| *rsm2* | .034 | .103 | .731 | .979 | .629 | .627 | .396 |
| *rsm3* | .006 | .006 | .329 | .659 | .610 | .980 | .964 |
| *rsm1 rsm2* | .507 | .438 | .042 | .324 | .551 | .839 | .721 |
| *rsm1 rsm2 rsm3* | .316 | .025 | .013 | .114 | .298 | .612 | .376 |
| *OX-9* | .000 | .000 | .000 | .000 | .029 | .674 | .881 |
| *OX-12* | .001 | .000 | .000 | .000 | .000 | .000 | .000 |
|  |  |  |  |  |  |  |  |
| Fig 2F: 200 mM NaCl | | | | | | | |
|  | Day 1 | Day 2 | Day 3 | Day 4 | Day 5 | Day 6 | Day 7 |
| *rsm1* |  | .001 | .001 | .031 | .120 | .047 | .190 |
| *rsm2* |  | .002 | .030 | .011 | .023 | .009 | .042 |
| *rsm3* |  | .002 | .050 | .038 | .054 | .039 | .141 |
| *rsm1 rsm2* |  | .891 | .006 | .060 | .094 | .050 | .098 |
| *rsm1 rsm2 rsm3* |  | .225 | .000 | .006 | .012 | .006 | .025 |
| *OX-9* |  | .000 | .000 | .000 | .000 | .000 | .000 |
| *OX-12* |  | .000 | .000 | .000 | .000 | .000 | .000 |
|  |  |  |  |  |  |  |  |
| Fig 2G: 200 mM Mannitol | | | | | | | |
|  | Day 1 | Day 2 | Day 3 | Day 4 | Day 5 | Day 6 | Day 7 |
| *rsm1* | .658 | .767 | .920 | .925 | .915 | .971 | .964 |
| *rsm2* | .160 | .616 | .945 | .842 | .804 | .804 | .761 |
| *rsm3* | .081 | .190 | .287 | .412 | .322 | .114 | .079 |
| *rsm1 rsm2* | .711 | .812 | .816 | .916 | .828 | .842 | .807 |
| *rsm1 rsm2 rsm3* | .426 | .977 | .831 | .954 | .873 | .794 | .749 |
| *OX-9* | .016 | .003 | .001 | .004 | .002 | .000 | .000 |
| *OX-12* | .057 | .000 | .000 | .000 | .000 | .000 | .000 |
