# Supplementary material for: RSM1, an Arabidopsis MYB protein, interacts with HY5/HYH to modulate seed germination and seedling development in response to abscisic acid and salinity
Source: PLoS Genet. 2018 Dec 19;14(12):e1007839. doi: 10.1371/journal.pgen.1007839 (PMC6317822; doi:10.1371/journal.pgen.1007839)
Supplement: S2 Table — (DOCX) [file pgen.1007839.s014.docx]

**S2 Table. *P*-values from comparisons between each genotype and Col-0 in terms of cotyledon greening rates in S2A-D Fig. The data were tested by one-way ANOVA, followed by LSD test using IBM SPSS Statistics Version 20.0.**

| S2A Fig: MS | | | | | | | | | | | | | |
| --- | --- | --- | --- | --- | --- | --- | --- | --- | --- | --- | --- | --- | --- |
|  | Day1 | | Day2 | | Day3 | | Day4 | | Day5 | | Day6 | | Day7 |
| *rsm1* | .583 | | .725 | | .346 | | .508 | | .704 | |  | |  |
| *rsm2* | .053 | | .380 | | .891 | | .504 | | .749 | |  | |  |
| *rsm3* | .053 | | .948 | | .953 | | .508 | | .704 | |  | |  |
| *rsm1 rsm2* | .116 | | .925 | | .466 | | .259 | | .223 | |  | |  |
| *rsm1 rsm2 rsm3* | .119 | | .618 | | .507 | | .508 | | .704 | |  | |  |
| *OX-9* | .656 | | .226 | | .089 | | .221 | | .166 | |  | |  |
| *OX-12* | .791 | | .797 | | .181 | | .124 | | .486 | |  | |  |
|  |  |  | |  | |  | |  | |  | |  | |
| S2B Fig: 1 μM ABA | | | | | | | | | | | | | |
|  | Day1 | | Day2 | | Day3 | | Day4 | | Day5 | | Day6 | | Day7 |
| *rsm1* |  | |  | |  | | .104 | | .264 | | .558 | | .923 |
| *rsm2* |  | |  | |  | | .322 | | .009 | | .015 | | .040 |
| *rsm3* |  | |  | |  | | .255 | | .183 | | .585 | | .882 |
| *rsm1 rsm2* |  | |  | |  | | .988 | | .060 | | .120 | | .110 |
| *rsm1 rsm2 rsm3* |  | |  | |  | | .793 | | .054 | | .001 | | .036 |
| *OX-9* |  | |  | |  | | .016 | | .000 | | .000 | | .000 |
| *OX-12* |  | |  | |  | | .016 | | .000 | | .000 | | .000 |
|  |  |  | |  | |  | |  | |  | |  | |
| S2C Fig: 100 mM NaCl | | | | | | | | | | | | | |
|  | Day1 | | Day2 | | Day3 | | Day4 | | Day5 | | Day6 | | Day7 |
| *rsm1* |  | | .430 | | .425 | | .791 | | .952 | | .758 | | .763 |
| *rsm2* |  | | .006 | | .000 | | .381 | | .158 | | .341 | | .065 |
| *rsm3* |  | | .005 | | .000 | | .119 | | .019 | | .325 | | .302 |
| *rsm1 rsm2* |  | | .166 | | .983 | | .717 | | .706 | | .876 | | 1.000 |
| *rsm1 rsm2 rsm3* |  | | .061 | | .843 | | .932 | | .993 | | .865 | | 1.000 |
| *OX-9* |  | | .016 | | .000 | | .000 | | .000 | | .081 | | .026 |
| *OX-12* |  | | .054 | | .000 | | .000 | | .000 | | .000 | | .016 |
|  |  |  | |  | |  | |  | |  | |  | |
| S2D Fig: 200 mM Mannitol | | | | | | | | | | | | | |
|  | Day1 | | Day2 | | Day3 | | Day4 | | Day5 | | Day6 | | Day7 |
| *rsm1* |  | | .247 | | .046 | | .827 | | .403 | | .984 | | .968 |
| *rsm2* |  | | .057 | | .001 | | .404 | | .411 | | .777 | | .782 |
| *rsm3* |  | | .045 | | .000 | | .052 | | .001 | | .486 | | .410 |
| *rsm1 rsm2* |  | | .767 | | .144 | | .899 | | .150 | | .734 | | .695 |
| *rsm1 rsm2 rsm3* |  | | .992 | | .013 | | .528 | | .802 | | .993 | | .987 |
| *OX-9* |  | | .045 | | .000 | | .000 | | .000 | | .001 | | .001 |
| *OX-12* |  | | .082 | | .000 | | .000 | | .000 | | .000 | | .023 |
