# Supplementary material for: RSM1, an Arabidopsis MYB protein, interacts with HY5/HYH to modulate seed germination and seedling development in response to abscisic acid and salinity
Source: PLoS Genet. 2018 Dec 19;14(12):e1007839. doi: 10.1371/journal.pgen.1007839 (PMC6317822; doi:10.1371/journal.pgen.1007839)
Supplement: S3 Table — (DOCX) [file pgen.1007839.s015.docx]

**S3 Table. *P*-values from comparisons between each genotype and Col-0 in terms of germination rates or cotyledon greening rates in Fig 5B-G. The data were tested by one-way ANOVA, followed by LSD test using IBM SPSS Statistics Version 20.0.**

| Fig 5B: MS-germination rate | | | | | | | |
| --- | --- | --- | --- | --- | --- | --- | --- |
|  | Day 1 | Day 2 | Day 3 | Day 4 | Day 5 | Day 6 | Day 7 |
| *OX-12* | .844 | .890 | .907 | .930 | .919 | .902 | .656 |
| *abi5-7* | .062 | .001 | .000 | .000 | .001 | .001 | .003 |
| *OX-12 abi5-7* | .134 | .003 | .002 | .003 | .001 | .002 | .014 |

| Fig 5C: 1 μM ABA-germination rate | | | | | | | |
| --- | --- | --- | --- | --- | --- | --- | --- |
|  | Day 1 | Day 2 | Day 3 | Day 4 | Day 5 | Day 6 | Day 7 |
| *OX-12* | .000 | .000 | .008 | .036 | .264 | .480 | .223 |
| *abi5-7* | .069 | .000 | .034 | .021 | .000 | .000 | .000 |
| *OX-12 abi5-7* | .000 | .012 | .528 | .325 | .470 | .510 | .263 |

| Fig 5D: 3 μM ABA-germination rate | | | | | | | |
| --- | --- | --- | --- | --- | --- | --- | --- |
|  | Day 1 | Day 2 | Day 3 | Day 4 | Day 5 | Day 6 | Day 7 |
| *OX-12* | .000 | .000 | .000 | .000 | .000 | .002 | .063 |
| *abi5-7* | .502 | .008 | .000 | .002 | .000 | .001 | .000 |
| *OX-12 abi5-7* | .000 | .000 | .000 | .000 | .000 | .006 | .941 |

| Fig 5E: 5 μM ABA-germination rate | | | | | | | |
| --- | --- | --- | --- | --- | --- | --- | --- |
|  | Day 1 | Day 2 | Day 3 | Day 4 | Day 5 | Day 6 | Day 7 |
| *OX-12* | .020 | .000 | .000 | .000 | .000 | .000 | .001 |
| *abi5-7* | .725 | .377 | .010 | .000 | .000 | .000 | .000 |
| *OX-12 abi5-7* | .002 | .000 | .000 | .000 | .001 | .000 | .052 |

| Fig 5F: MS-cotyledon greening rate | | | | | | | |
| --- | --- | --- | --- | --- | --- | --- | --- |
|  | Day 1 | Day 2 | Day 3 | Day 4 | Day 5 | Day 6 | Day 7 |
| *OX-12* |  | .473 | .686 | .407 | .332 | .285 | 1.000 |
| *abi5-7* |  | .886 | .005 | .004 | .006 | .058 | .566 |
| *OX-12 abi5-7* |  | .316 | .144 | .044 | .057 | .039 | .236 |

| Fig 5G: 1 μM ABA-cotyledon greening rate | | | | | | | |
| --- | --- | --- | --- | --- | --- | --- | --- |
|  | Day 1 | Day 2 | Day 3 | Day 4 | Day 5 | Day 6 | Day 7 |
| *OX-12* |  |  | .000 | .000 | .000 | .000 | .000 |
| *abi5-7* |  |  | 1.000 | .310 | .001 | .000 | .000 |
| *OX-12 abi5-7* |  |  | .000 | .000 | .000 | .000 | .000 |
