# Supplementary material for: RSM1, an Arabidopsis MYB protein, interacts with HY5/HYH to modulate seed germination and seedling development in response to abscisic acid and salinity
Source: PLoS Genet. 2018 Dec 19;14(12):e1007839. doi: 10.1371/journal.pgen.1007839 (PMC6317822; doi:10.1371/journal.pgen.1007839)
Supplement: S4 Table — (DOCX) [file pgen.1007839.s016.docx]

**S4 Table. *P*-values from comparisons between each genotype and Col-0 in terms of germination rates or cotyledon greening rates in S8B-D, F-H Fig. The data were tested by one-way ANOVA, followed by LSD test using IBM SPSS Statistics Version 20.0.**

| S8B Fig: MS-germination rate | | | | | | | |
| --- | --- | --- | --- | --- | --- | --- | --- |
|  | Day 1 | Day 2 | Day 3 | Day 4 | Day 5 | Day 6 | Day 7 |
| *OX-12* | .245 | .503 | .666 | .595 | .373 |  |  |
| *abi3-8* | .517 | .705 | .908 | .828 | .576 |  |  |
| *OX-12 abi3-8* | .376 | .000 | .001 | .005 | .003 |  |  |

| S8C Fig: 1 μM ABA-germination rate | | | | | | | |
| --- | --- | --- | --- | --- | --- | --- | --- |
|  | Day 1 | Day 2 | Day 3 | Day 4 | Day 5 | Day 6 | Day 7 |
| *OX-12* | .354 | .000 | .000 | .001 | .095 | .047 | .211 |
| *abi3-8* | .000 | .000 | .000 | .000 | .407 | .598 | .348 |
| *OX-12 abi3-8* | .000 | .000 | .002 | .000 | .460 | .430 | .926 |
|  |  |  |  |  |  |  |  |
| S8D Fig: 5 μM ABA-germination rate | | | | | | | |
|  | Day 1 | Day 2 | Day 3 | Day 4 | Day 5 | Day 6 |  |
| *OX-12* | 1.000 | .373 | .009 | .000 | .000 | .005 | .000 |
| *abi3-8* | .031 | .000 | .000 | .000 | .000 | .000 | .000 |
| *OX-12 abi3-8* | .162 | .000 | .000 | .000 | .000 | .000 | .006 |
|  |  |  |  |  |  |  |  |
| S8F Fig: MS-cotyledon greening rate | | | | | | | |
|  | Day 1 | Day 2 | Day 3 | Day 4 | Day 5 | Day 6 | Day 7 |
| *OX-12* |  | .068 | .132 | .436 | .609 | 1.000 |  |
| *abi3-8* |  | .021 | .943 | .132 | .473 | 1.000 |  |
| *OX-12 abi3-8* |  | .015 | .167 | .180 | .015 | .195 |  |
|  |  |  |  |  |  |  |  |
| S8G Fig: 1 μM ABA-cotyledon greening rate | | | | | | | |
|  | Day 1 | Day 2 | Day 3 | Day 4 | Day 5 | Day 6 | Day 7 |
| *OX-12* |  |  | 1.000 | .014 | .000 | .000 | .000 |
| *abi3-8* |  |  | .000 | .000 | .000 | .000 | .000 |
| *OX-12 abi3-8* |  |  | .000 | .000 | .000 | .000 | .000 |

| S8H Fig: 5 μM ABA-cotyledon greening rate | | | | | | | |
| --- | --- | --- | --- | --- | --- | --- | --- |
|  | Day 1 | Day 2 | Day 3 | Day 4 | Day 5 | Day 6 | Day 7 |
| *OX-12* |  |  |  | 1.000 | 1.000 | 1.000 | 1.000 |
| *abi3-8* |  |  |  | .000 | .000 | .000 | .000 |
| *OX-12 abi3-8* |  |  |  | .000 | .000 | .000 | .000 |
