# Supplementary material for: RSM1, an Arabidopsis MYB protein, interacts with HY5/HYH to modulate seed germination and seedling development in response to abscisic acid and salinity
Source: PLoS Genet. 2018 Dec 19;14(12):e1007839. doi: 10.1371/journal.pgen.1007839 (PMC6317822; doi:10.1371/journal.pgen.1007839)
Supplement: S5 Table — (DOCX) [file pgen.1007839.s017.docx]

**S5 Table. *P*-values from comparisons between each genotype and Col-0 in terms of germination rates or cotyledon greening rates in S9B-G Fig. The data were tested by one-way ANOVA, followed by LSD test using IBM SPSS Statistics Version 20.0.**

| S9B Fig: MS-germination rate | | | | | | | |
| --- | --- | --- | --- | --- | --- | --- | --- |
|  | Day 1 | Day 2 | Day 3 | Day 4 | Day 5 | Day 6 | Day 7 |
| *OX-12* | .001 | .000 | .001 | .001 | .003 | .006 | .042 |
| *abi4-1* | .690 | .925 | .911 | .744 | .765 | .739 | .829 |
| *OX-12 abi4-1* | .006 | .001 | .001 | .000 | .002 | .002 | .047 |

| S9C Fig: 1 μM ABA-germination rate | | | | | | | |
| --- | --- | --- | --- | --- | --- | --- | --- |
|  | Day 1 | Day 2 | Day 3 | Day 4 | Day 5 | Day 6 | Day 7 |
| *OX-12* | .001 | .000 | .001 | .003 | .001 | .000 | .000 |
| *abi4-1* | .000 | .289 | .029 | .697 | .949 | .773 | .667 |
| *OX-12 abi4-1* | .000 | .925 | .578 | .697 | .949 | .773 | .667 |
|  |  |  |  |  |  |  |  |
| S9D Fig: 3 μM ABA-germination rate | | | | | | | |
|  | Day 1 | Day 2 | Day 3 | Day 4 | Day 5 | Day 6 | Day 7 |
| *OX-12* | .166 | .000 | .000 | .000 | .000 | .000 | .000 |
| *abi4-1* | .000 | .000 | .000 | .001 | .011 | .023 | .018 |
| *OX-12 abi4-1* | .000 | .000 | .055 | .452 | .644 | .791 | .661 |

| S9E Fig: 5 μM ABA-germination rate | | | | | | | |
| --- | --- | --- | --- | --- | --- | --- | --- |
|  | Day 1 | Day 2 | Day 3 | Day 4 | Day 5 | Day 6 | Day 7 |
| *OX-12* | .885 | .001 | .000 | .000 | .000 | .000 | .001 |
| *abi4-1* | .000 | .000 | .000 | .000 | .000 | .000 | .014 |
| *OX-12 abi4-1* | .000 | .000 | .004 | .035 | .325 | .866 | .586 |

| S9F Fig: MS-cotyledon greening rate | | | | | | | |
| --- | --- | --- | --- | --- | --- | --- | --- |
|  | Day 1 | Day 2 | Day 3 | Day 4 | Day 5 | Day 6 | Day 7 |
| *OX-12* |  | .462 | .200 | .036 | .050 | .116 | .421 |
| *abi4-1* |  | .688 | .848 | 1.000 | 1.000 | 1.000 | 1.000 |
| *OX-12 abi4-1* |  | .753 | .080 | .004 | .009 | .095 | .291 |

| S9G Fig: 1 μM ABA -cotyledon greening rate | | | | | | | |
| --- | --- | --- | --- | --- | --- | --- | --- |
|  | Day 1 | Day 2 | Day 3 | Day 4 | Day 5 | Day 6 | Day 7 |
| *OX-12* |  | 1.000 | 1.000 | .542 | .175 | .001 | .000 |
| *abi4-1* |  | .005 | .000 | .000 | .000 | .000 | .000 |
| *OX-12 abi4-1* |  | .000 | .000 | .000 | .000 | .000 | .000 |
