# Supplementary material for: RSM1, an Arabidopsis MYB protein, interacts with HY5/HYH to modulate seed germination and seedling development in response to abscisic acid and salinity
Source: PLoS Genet. 2018 Dec 19;14(12):e1007839. doi: 10.1371/journal.pgen.1007839 (PMC6317822; doi:10.1371/journal.pgen.1007839)
Supplement: S6 Table — (DOCX) [file pgen.1007839.s018.docx]

**S6 Table. *P*-values of comparisons between each genotype and Col-0 in terms of seed germination rates in Fig 7B-G. The data were tested by one-way ANOVA, followed by LSD test using IBM SPSS Statistics Version 20.0.**

| Fig 7B: MS-germination rate | | | | | | | |
| --- | --- | --- | --- | --- | --- | --- | --- |
|  | Day 1 | Day 2 | Day 3 | Day 4 | Day 5 | Day 6 | Day 7 |
| *OX-12* | .278 | .814 | .538 | .361 | 1.000 | 1.000 |  |
| *hy5* | .005 | .146 | .274 | .361 | 1.000 | 1.000 |  |
| *hyh* | .162 | .118 | .892 | .361 | 1.000 | 1.000 |  |
| *OX-12 hy5* | .005 | .017 | .026 | .002 | .109 | .109 |  |
| *OX-12 hyh* | .121 | .070 | .538 | .361 | 1.000 | 1.000 |  |

| Fig 7C: 1 μM ABA-germination rate | | | | | | | |
| --- | --- | --- | --- | --- | --- | --- | --- |
|  | Day 1 | Day 2 | Day 3 | Day 4 | Day 5 | Day 6 | Day 7 |
| *OX-12* | 1.000 | .002 | .000 | .000 | .000 | .151 | .383 |
| *hy5* | 1.000 | .005 | .184 | .326 | .340 | .672 | .814 |
| *hyh* | 1.000 | .001 | .752 | .199 | .308 | .400 | .473 |
| *OX-12 hy5* | .000 | .003 | .000 | .061 | .228 | .124 | .159 |
| *OX-12 hyh* | 1.000 | .000 | .000 | .001 | .000 | .006 | .055 |

| Fig 7D: 3 μM ABA-germination rate | | | | | | | |
| --- | --- | --- | --- | --- | --- | --- | --- |
|  | Day 1 | Day 2 | Day 3 | Day 4 | Day 5 | Day 6 | Day 7 |
| *OX-12* | 1.000 | .002 | .000 | .005 | .000 | .000 | .000 |
| *hy5* | 1.000 | .308 | .415 | .004 | .000 | .000 | .001 |
| *hyh* | 1.000 | .489 | .532 | .263 | .048 | .073 | .024 |
| *OX-12 hy5* | .001 | .101 | .002 | .153 | .001 | .112 | .378 |
| *OX-12 hyh* | 1.000 | .001 | .000 | .002 | .000 | .000 | .000 |

| Fig 7E: 5 μM ABA-germination rate | | | | | | | |
| --- | --- | --- | --- | --- | --- | --- | --- |
|  | Day 1 | Day 2 | Day 3 | Day 4 | Day 5 | Day 6 | Day 7 |
| *OX-12* |  | .054 | .184 | .000 | .000 | .000 | .000 |
| *hy5* |  | .602 | .000 | .000 | .000 | .001 | .002 |
| *hyh* |  | .060 | .000 | .000 | .001 | .197 | .049 |
| *OX-12 hy5* |  | .370 | .026 | .056 | .002 | .005 | .000 |
| *OX-12 hyh* |  | .054 | .002 | .000 | .000 | .000 | .000 |

| Fig 7F: 100 mM NaCl-germination rate | | | | | | | |
| --- | --- | --- | --- | --- | --- | --- | --- |
|  | Day 1 | Day 2 | Day 3 | Day 4 | Day 5 | Day 6 | Day 7 |
| *OX-12* | 1.000 | .000 | .163 | .076 | .014 | .028 | .201 |
| *hy5* | 1.000 | .005 | .902 | .723 | .414 | .407 | .566 |
| *hyh* | 1.000 | .004 | .922 | .903 | .690 | .685 | .686 |
| *OX-12 hy5* | .109 | .000 | .001 | .000 | .000 | .000 | .000 |
| *OX-12 hyh* | 1.000 | .000 | .093 | .024 | .064 | .306 | .496 |

| Fig 7G: 200 mM NaCl-germination rate | | | | | | | |
| --- | --- | --- | --- | --- | --- | --- | --- |
|  | Day 1 | Day 2 | Day 3 | Day 4 | Day 5 | Day 6 | Day 7 |
| *OX-12* |  | 1.000 | .006 | .000 | .000 | .004 | .007 |
| *hy5* |  | .164 | .000 | .021 | .944 | .895 | .549 |
| *hyh* |  | 1.000 | .013 | .285 | .786 | .789 | .847 |
| *OX-12 hy5* |  | .388 | .064 | .001 | .008 | .020 | .020 |
| *OX-12 hyh* |  | 1.000 | .006 | .000 | .001 | .002 | .006 |
