# Supplementary material for: RSM1, an Arabidopsis MYB protein, interacts with HY5/HYH to modulate seed germination and seedling development in response to abscisic acid and salinity
Source: PLoS Genet. 2018 Dec 19;14(12):e1007839. doi: 10.1371/journal.pgen.1007839 (PMC6317822; doi:10.1371/journal.pgen.1007839)
Supplement: S7 Table — (DOCX) [file pgen.1007839.s019.docx]

Table S7. List of primers used in this study.

| **Primer name** | Primer sequences (5’→3’)  (Note: The underlined nucleotides indicate the restriction endonuclease enzymes for cloning.) | | |
| --- | --- | --- | --- |
| **Genotyping** | | | |
| *cs876657* (F) | TGGATGGAAAAACGCAATCTTTGG | | |
| *cs876657* (R) | TCTCATCGATTTCGCTGGAATTTG | | |
| *cs876657* (LB) | TAGCATCTGAATTTCATAACCAATCTCGATACAC | | |
| *cs371942* (F) | AATTTAGTCACCCTGATGGGC | | |
| *cs371942* (R) | ATTTGCTACATCTGGCACCAC | | |
| pAC161-LB | ATATTGACCATCATACTCATTGC | | |
| *Salk_069941C* (F) | TCGATATGATCAATCGGTTCC | | |
| *Salk_069941C* (R) | TGTGTCAAAGTCATTGATGCTTC | | |
| *hy5-215* (F) | CTTCAGCCGCTTGTTCTCTTTC | | |
| *hy5-215* (R) | CAGGAACAAGCGACTAGCTC | | |
| *hyh* (F) | ACTCGCATAAGAACATGTGGG | | |
| *hyh* (R) | ACCCACACGCTCTGTGAATAC | | |
| *abi3-8* (F) | CGTTGTTCCGATGATGGAG | | |
| *abi3-8* (R) | TGTATCCATTGAAACCTGTTGG | | |
| *abi4-1* (F) | GAGATCCGAGAGCCACGTAA | | |
| *abi4-1* (F) | CTAACGCCACCTCATGATGA | | |
| *abi5-7* (F) | CGTGAATAGCTGAACAGGGAC | | |
| *abi5-7* (R) | CACTACTTTCTCCACTGGACCA | | |
| *OX-12* (F) | GGCAACATTTGACTGTGTGTG | | |
| *OX-12* (R) | ATGCCCTTGTCTCGTTCTTG | | |
| pBI121-SP3-LB | AATCAGCTGTTGCCCGTCTCAC | | |
| pDs-Lox-LB | AACGTCCGCAATGTGTTATTAAGTTGTC | | |
| SALK_LBb1.3 | ATTTTGCCGATTTCGGAAC | | |
| **Quantitative Real-time RT-PCR** | | | |
| *RSM1* (F) | CAGTATGACCTTCTAGTTCGTGACA | | |
| *RSM1* (R) | TGCAGCTTCATGCTTCTCAT | | |
| *RSM2* (F) | ACACTACGAACTCTTAGTGCAA | | |
| *RSM2* (R) | CTCCACTAGTCCTGTAGTTTGG | | |
| *RSM3* (F) | AAGCTGTCGGAGGGAAAACT | | |
| *RSM3* (R) | GAAGGTCTTGTAATTGGGCAAA | | |
| *RSM4* (F) | AACGTGGCTAAAGCAGTTGG | | |
| *RSM4* (R) | ACTTCCAACATCGACGGTTT | | |
| *HY5* (F) | TTCAGAACGAGAACCAGATGC | | |
| *HY5* (R) | AGAAGGAGATCAAAGGCTTGC | | |
| *HYH* (F) | AGAACAACAATGACCAGCTCG | | |
| *HYH* (R) | CAACACTGAACAATGGATTAAAGG | | |
| *ABI1* (F) | CGGCAAAACTGCACTTCCATT | | |
| *ABI1* (R) | CACGAGCTCCATTCCACTGAA | | |
| *ABI2* (F) | CTCGCAATGTCAAGATCCATTGGC | | |
| *ABI2* (R) | TTACTCGCCGCACTGAAGTCAC | | |
| *ABI3* (F) | GATTGAATCAGCGGCAAGAA | | |
| *ABI3* (R) | GTTGTTGTGGTGGTGGAGGA | | |
| *ABI4* (F) | ATCCTCAATCCGATTCCACC | | |
| *ABI4* (R) | ATTTGCCCCAGCTTCTTTGT | | |
| *ABI5* (F) | GAGATTGCGGACATTGATGA | | |
| *ABI5* (R) | CCTCGTTCTGTCTCCGACTT | | |
| *RD29A* (F) | GGTGCGACTGATGAGGTGAA | | |
| *RD29A* (R) | CGGAATGAGGCGATTTTGGC | | |
| *RD29B* (F) | AGAAGGAATGGTGGGGAAAG | | |
| *RD29B* (R) | CAACTCACTTCCACCGGAAT | | |
| *RAB18* (F) | GGTTGGATTTTGATCGATGG | | |
| *RAB18* (R) | CCAACACCAGAATCTCCGAT | | |
| *AtEM1* (F) | CCTGCTTGTAGGTTCCGTGT | | |
| *AtEM1* (R) | CGGTACAACACTGCCGTTAG | | |
| *AtEM6* (F) | GGAGCAGTTAGGAACTGAAGGAT | | |
| *AtEM6* (R) | TTTGGATTCGTCTATCTCGACTC | | |
| *SnRK2.2* (F) | TTGTTAGATGGAAGTCCGGCAC | | |
| *SnRK2.2* (R) | TTGGGAATGAAGAACAGAAGACT | | |
| *SnRK2.3* (F) | CATACATCGCTCCAGAGGTACT | | |
| *SnRK2.3* (R) | GATACGCTCCAACCAACATGAC | | |
| *ABF2* (F) | TTGGGGAATGAGCCACCAGGAG | | |
| *ABF2* (R) | GACCCAAAATCTTTCCCTACAC | | |
| *ABF3* (F) | CTTTGTTGATGGTGTGAGTGAG | | |
| *ABF3* (R) | GTGTTTCCACTATTACCATTGC | | |
| *ABF4* (F) | GCTCGAAAGCAGGCTTATACA | | |
| *ABF4* (R) | TCTGCATTTCCACCATTTCA | | |
| *PP2A* (F) | TATCGGATGACGATTCTTCGTGCAG | | |
| *PP2A* (R) | GCTTGGTCGACTATCGGAATGAGAG | | |
| *ACTIN2* (F) | GCACCCTGTTCTTCTTACCGAG | | |
| *ACTIN2* (R) | AGTAAGGTCACGTCCAGCAAGG | | |
| **ChIP-qPCR** |  | | |
| *ABI5*-A (F) | CGATGTGGACCGTTCTTCTT | | |
| *ABI5*-A (R) | GTTGTCCCTTATTCAACTATCACG | | |
| *ABI5*-B (F) | CGAGTGGGTTAAGATATTTTCCTC | | |
| *ABI5*-B (R) | ATCGTGACAGCGACAAACG | | |
| **EMSA** |  | | |
| *RSM1*-Probe  (HY5/ HYH binds p*RSM1*) | biotin-ATCTAGAGAATGACGTCATAACCACTAA | | |
| *ABI5*-Probe | biotin-CCTTATGCAGTGAATAGTCCACGTGCACTCCCAATGGAAGTTCGGAATC  biotin-GATTCCGAACTTCCATTGGGAGTGCACGTGGACTATTCACTGCATAAGG | | |
| *RSM1*-Probe P1 | biotin-GAGACTTGTCCCTAACTTTCAACCAAAGACTTGTAATTTTCCGCGAGCAC | | |
| *RSM1*-Probe P2 | biotin-GTGAGCTTTGTGCTCTGATTAGAGACCAGACTGTTCCATGAATTAAGTAC | | |
| **Yeast one-hybrid assay** | | |  |
| RSM1-EcoRI (F) | CCGGAATTCATGGCATCAGGCTCAATGTC | | pB42AD-RSM1 |
| RSM1-XhoI (R) | CCGCTCGAGTCACTGCAGCTTCATGCTTC | |  |
| HY5-EcoRI (F) | CCGGAATTCATGCAGGAACAAGCGACTAG | | pB42AD-HY5 |
| HY5-XhoI (R) | CCGCTCGAGTCAAAGGCTTGCATCAGCAT | |  |
| HYH-EcoRI (F) | CGGAATTCATGTCTCTCCAACGACCCA | | pB42AD-HYH |
| HYH-EcoRI (R) | CGGAATTCTTAGTGATTGTCATCAGTTTTAGGC | |  |
| *ABI5*pro-A-KpnI (F) | CCGGAATTCGATGTGGACCGTTCTTCTTT | | pLacZ2u-*ABI5*pro-A |
| *ABI5*pro-A-XhoI (R) | CCGCTCGAGTTAACAACTGCATCATATACACAAC | |  |
| *ABI5*pro-B-KpnI (F) | CCGGAATTCGTCTTCGTTTTCTGGTCCTTAC | | pLacZ2u-*ABI5*pro-B |
| *ABI5*pro-B-XhoI (R) | CCGCTCGAGGTGACAGCGACAAACGATAC | |  |
| *ABI5*pro-C-KpnI (F) | CCGGAATTCACCCATTTATCTCTCTCTTTCTCAA | | pLacZ2u-*ABI5*pro-C |
| *ABI5*pro-C-XhoI (R) | CCGCTCGAGAAGGACCAGAAAACGAAGACC | |  |
| *ABI5*pro-D-KpnI (F) | CCGGAATTCTGTGTAGCCGAAGTCACACG | | pLacZ2u-*ABI5*pro-D |
| *ABI5*pro-D-XhoI (R) | CCGCTCGAGAGGAGGCGGTAAAAGAGAGATT | |  |
| *ABI5*pro-E- KpnI (F) | CCGGAATTCTTTCACCAGCTAGAAGCTCAAC | | pLacZ2u-*ABI5*pro-E |
| *ABI5*pro-E- XhoI (R) | CCGCTCGAGCGTGTGACTTCGGCTACACA | |  |
| *ABI5*pro-F-KpnI (F) | CCGGAATTCTAGTTGCTGTAATCTTTAGGTCGC | | pLacZ2u-*ABI5*pro-F |
| *ABI5*pro-F-XhoI (R) | CCGCTCGAGGTTGAGCTTCTAGCTGGTGAAA | |  |
| *RSM1*pro-A- SmaI (F) | TCCCCCGGGCGCAATCTTTGGTTGATTAG | | pLacZ2u- *RSM1*pro-A |
| *RSM1*pro-A-XhoI (R) | TCGCTCGAGGCCTGATGCCATTGTTTAGT | |  |
| *RSM1*pro-B- SmaI (F) | TCCCCCGGGGAGACTTGTCCCTAACTTTCA | | pLacZ2u- *RSM1*pro-B |
| *RSM1*pro-B-XhoI (R) | TCGCTCGAGTTCCCAATTAGTGGTTATGAC | |  |
| *RSM1*pro-C- SmaI (F) | TCCCCCGGGAACACAGAAAAATGTCGATG | | pLacZ2u- *RSM1*pro-C |
| *RSM1*pro-C-XhoI (R) | TCGCTCGAGGTCTTTGGTTGAAAGTTAGG | |  |
| *RSM1*pro-D- SmaI (F) | TCCCCCGGGGTAATACGACAACTTTTGGTTC | | pLacZ2u- *RSM1*pro-D |
| *RSM1*pro-D-XhoI (R) | TCGCTCGAGCTGTGTTTTCTTGTTGTTGA | |  |
| **BiFC** |  |  | |
| RSM1-SpeI(F) | GGACTAGTATGGCATCAGGCTCAATGTC | pSY736-RSM1 | |
| RSM1-BamHI(R) | CGGGATCCTCACTGCAGCTTCATGCTTC |  |  |
| HY5-SacI(F) | CGAGCTCATGCAGGAACAAGCGACTAG | pSY735-HY5 | |
| HY5-SpeI(R) | GGACTAGTTCAAAGGCTTGCATCAGC |  |  |
| HYH-SacI(F) | CGAGCTCATGTCTCTCCAACGACCCA | pSY735-HYH | |
| HYH-SpeI(R) | GGACTAGTTTAGTGATTGTCATCAGTTTTAGGC |  |  |
| ABI5-SacI(F) | CGAGCTCATGGTAACTAGAGAAACGAAGTTG | pSY735-ABI5 | |
| ABI5-SpeI(R) | GGACTAGTTTAGAGTGGACAACTCGGGT |  |  |
| **Protein expression** |  |  | |
| RSM1-BamHI(F) | CGGGATCCATGGCATCAGGCTCAATGTC | pET28a-RSM1 | |
| RSM1-SacI(R) | CGAGCTCCTCACTGCAGCTTCATGCTT |  |  |
| HY5-BamHI(F) | CGCGGATCCATGCAGGAACAAGCGACT | pEGX-6P-1-HY5 | |
| HY5-SalI(R) | ACGCGTCGACTTATCATCAAAGGCTTGCATCAGC |  |  |
| HYH-EcoRI(F) | CGGAATTCATGTCTCTCCAACGACCCA | pEGX-4T-1-HYH | |
| HYH-XhoI(R) | CCGCTCGAGTTAGTGATTGTCATCAGTTTTAGGC |  |  |
| ABI5-EcoRI(F) | CCGGAATTCATGGTAACTAGAGAAACGAAGTTG | pEGX-4T-1-ABI5 | |
| ABI5-XhoI(R) | CCGCTCGAGTTAGAGTGGACAACTCGGGT |  |  |
